# How Permanent Are the Permanent Macrodipoles of Anthranilamide Bioinspired Molecular Electrets?

**DOI:** 10.1021/jacs.3c10525

**Published:** 2024-01-16

**Authors:** Moon Young Yang, Omar O’Mari, William A. Goddard, Valentine I. Vullev

**Affiliations:** # Materials and Process Simulation Center, California Institute of Technology, Pasadena, California 91125, United States; ¶ Department of Bioengineering, University of California, Riverside, California 92521, United States; ± Department of Chemistry, University of California, Riverside, California 92521, United States; $ Department of Biochemistry, University of California, Riverside, California 92521, United States; § Materials Science and Engineering Program, University of California, Riverside, California 92521, United States

## Abstract

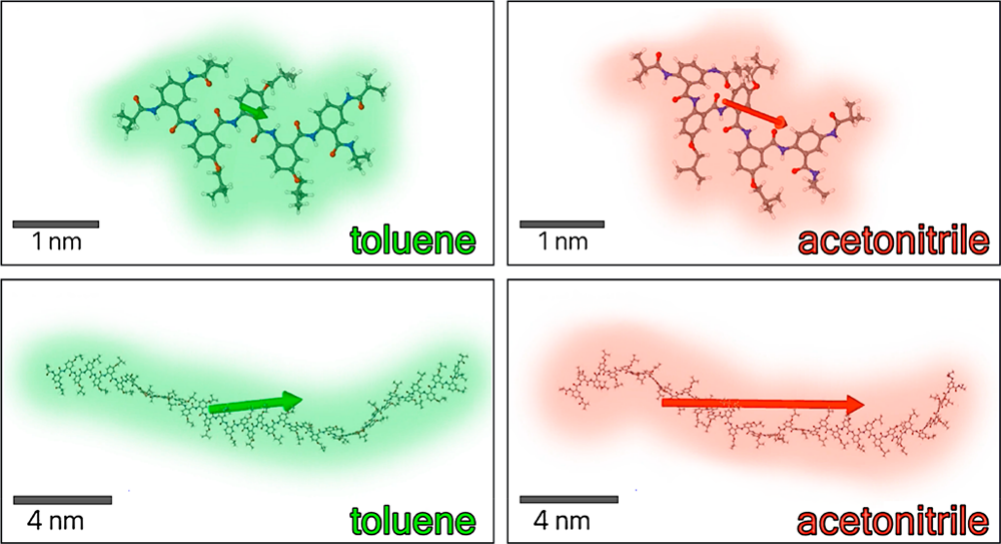

Dipoles are ubiquitous, and their impacts on materials and interfaces affect many aspects of daily life. Despite their importance, dipoles remain underutilized, often because of insufficient knowledge about the structures producing them. As electrostatic analogues of magnets, electrets possess ordered electric dipoles. Here, we characterize the structural dynamics of bioinspired electret oligomers based on anthranilamide motifs. We report dynamics simulations, employing a force field that allows dynamic polarization, in a variety of solvents. The results show a linear increase in macrodipoles with oligomer length that strongly depends on solvent polarity and hydrogen-bonding (HB) propensity, as well as on the anthranilamide side chains. An increase in solvent polarity increases the dipole moments of the electret structures while decreasing the dipole effects on the moieties outside the solvation cavities. The former is due to enhancement of the Onsager reaction field and the latter to screening of the dipole-generated fields. Solvent dynamics hugely contributes to the fluctuations and magnitude of the electret dipoles. HB with the solvent weakens electret macrodipoles without breaking the intramolecular HB that maintains their extended conformation. This study provides design principles for developing a new class of organic materials with controllable electronic properties. An animated version of the TOC graphic showing a sequence of the MD trajectories of short and long molecular electrets in three solvents with different polarities is available in the HTML version of this paper.

## Introduction

Originating from ordered polar moieties, the macrodipoles of molecular electrets offer key paradigms for implementation of electrostatics at nanometer scales, profoundly impacting phenomena, such as charge transfer (CT), molecular recognition, and interfacial interactions.^1−3^ Protein helices represent some of the best examples of molecular electrets.^4,5^ With macrodipoles of about 3–5 D per residue, protein α-helices can rectify CT and ensure the functionality of life-sustaining ion channels.^5−9^ The structural fragility of polypeptides composed of α-amino acids, however, limits their utility outside their native environment. Moreover, being susceptible to redox degradation, protein backbones mediate electron and hole transfer solely via tunneling, limiting the practical application of long-range CT to about 2 nm.^10,11^

**Figure 1:**
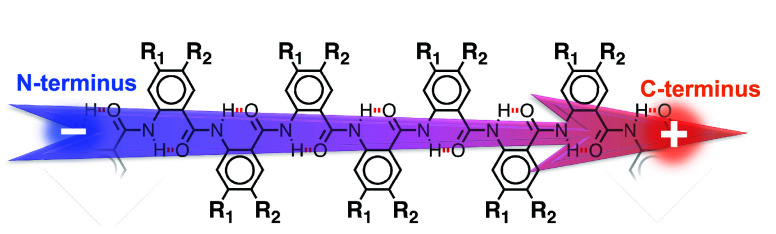
Schematic illustration of an AA bioinspired molecular electret with the macrodipole originating from ordered amide bonds and polarization induced by HB.

Similar to protein α- and 3_10_-helices,^12^ anthranilamide (AA) oligomers possess permanent macrodipoles originating from the ordered orientation of their amide and hydrogen bonds (HBs) (Figure 1).^13^ Unlike proteins, however, many AA structures exhibit reversible oxidation, and the aromatic moieties along their backbones provide sites for charge hopping important for long-range CT.^14^ Furthermore, the electric dipoles of AA residues strongly impact CT kinetics.^15,16^ As X-ray analysis shows, AA oligomers assume extended conformations,^17^ which was also supported by quantum mechanical (QM) calculations.^18^ Nevertheless, such QM analyses are applicable only to small oligomers, and X-ray crystallography provides only a “rigid” picture of the AA structures.

Using force fields (FFs) to describe structures and integrations as they evolve, molecular dynamics (MD) addresses these challenges facing experimental and QM-based assessment. Proven invaluable for chemistry, biology, and materials science, MD simulations go far beyond the time and length QM scales, provide fundamental insights into structural dynamics and physical properties, and produce important guidelines for experimental designs.^19^ The standard FFs, however, employ fixed point charges, imposing severe limitations in describing the fluctuating electrostatic environments present during the dynamics of polar conjugates, such as electrets.

In order to accurately describe dynamic electrostatic interactions involving charge and polarization fluctuations, we developed the polarizable charge equilibration (PQEq) method.^20^ PQEq implements Gaussian-shaped electron-density clouds on each atom and describes the charge and polarization fluctuations at the femtosecond time scale. Moreover, we used QM methods to develop a new generation of long-range nonbonded interactions, i.e., universal nonbonded (UNB) interactions, to describe van der Waals (vdW) attraction and Pauli repulsion interactions.^21^ These methods increase the accuracy of depicting the response to electric fields, providing a powerful tool for estimating the dipole dynamics of systems such as molecular electrets. For the MD simulations, we combine these UNB interactions with the valence bond, angle, and torsion characteristics of the universal force field (UFF), which has parameters for all atoms of the periodic table (up to Lr, *Z* = 103).^22^

Herein, MD simulations, combining this improved representation of electrostatic and nonbonded interactions, allow us to demonstrate the first dynamic description of the behavior of AA electrets immersed in explicitly introduced solvents with different properties. While the variations of the extended AA conformations are relatively small, our results show significant fluctuations of the AA permanent electric macrodipoles with a clear dependence on the oligomer length and solvent polarity. Moreover, solute–solvent HB interactions and the AA side chains emerge as important modifiers of the molecular dipoles, demonstrating the multifaceted nature of designing large polar systems, such as amide molecular electrets.

## Results and Discussion

### Initial Selection of Electrets and Solvents

**Figure 2:**
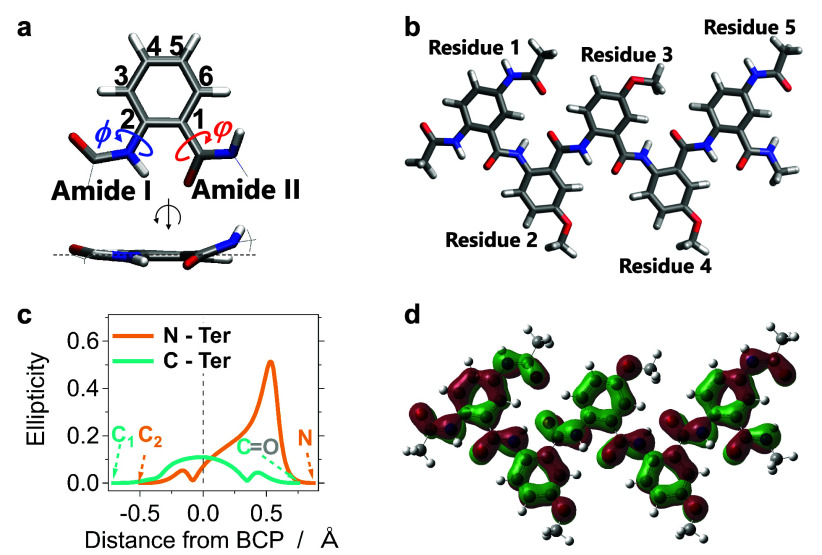
Structural and electronic DFT analyses of a Box AA pentamer. (a) DFT-optimized structure of a unit residue of the AA oligomer, where gray, white, red, and blue represent carbon, hydrogen, oxygen, and nitrogen, respectively. (b) DFT-optimized structure of the Aaa-capped Box oligomer. (c) Average *N*-terminus and *C*-terminus bond ellipticity for the Aaa-capped Box oligomer in the gas phase. (d) Localized molecular orbitals for a single resonance structure, showing the π-character of the bonds between the amides and the aromatic rings of the Aaa-capped Box oligomer (isovalue: 0.08).

Since AAs with ether substituents at position 5 manifest reversible electrochemical oxidation at relatively large positive potentials, making them feasible for transducing high-energy holes,^23^ our initial focus is on conjugates composed of Box residues possessing isobutyl ether groups as R_2_ side chains (Figures 1 and 2a,b). Conversely, an AA residue with an *N*-amide at position 5, denoted as Aaa, provides a means for covalent connectivity with favorable electronic coupling for hole injection by photoexcited electron acceptors.^24^ It motivates the selection of Aaa for capping the termini of the AA oligomers and polymers to form Aaa-Box_*l*–2_-Aaa, where the numbers of residues *l* = 5 for the density functional theory (DFT) analysis and up to 40 for the MD simulations. Since HB interactions along the backbone chain are important for maintaining the structural integrity of AA electrets, we employ solvents with various polarities that do not form HBs, i.e., toluene (Tol), dichloromethane (DCM), and acetonitrile (MeCN).

This study focuses on electret macrodipoles and their dynamics. Originating from displacement of positive and negative charges, dipoles depend on molecular geometry and electronic structure. Prior to diving into the MD analysis of the AA oligomers and their macrodipoles, therefore, it is paramount to discuss the electronic features of the bonding patterns along their backbones. Resorting to QM calculations, the next section demonstrates how the electronic structure of the bonds between the aromatic rings and the amides of the AA conjugates impacts their geometry.

### Are the AA Electrets Flat?

The common notion is that AA oligomers assume flat extended conformations.^17^ HBs between the amides at each residue and the π-conjugation with the aromatic rings favor planarity of these structures (Figure 1). Nonetheless, DFT calculations reveal close vdW contacts between (1) the AA hydrogen at position 3 and the oxygen of the *N*-terminal amide, i.e., amide I, as well as (2) the AA hydrogen at position 6 and the hydrogen of the *C*-terminal amide, i.e., amide II (Figures 2a and S2). Despite the HB and the π-conjugation along the AA backbone, this steric hindrance twists the amides slightly off the plane of the aromatic ring (Figure 2b and Table S9). Based on DFT calculations for Box-containing oligomers with five residues (*l* = 5), we find that an increase in solvent polarity shifts the dihedral angles between the amides and the aromatic rings away from 180°. That is, nonpolar solvents enhance the planarity of the oligomers (Table S9).

Amide I tends to be less twisted out of the aromatic ring plane than amide II; i.e., ϕ is closer to 180° than φ (Figure 2a and Table S9). In contrast, the vdW steric hindrance of amide I with the aromatic ring is larger than that of amide II (Figure 2a), considering the sizes of carbonyl oxygens and amide hydrogens and the lengths of C=O and N–H bonds. Differences in the π-conjugation of the two amides with the aromatic ring can elucidate the reason for this conundrum.

Analyzing the topology of the total charge density (ρ(**r**)) obtained from atoms-in-molecules (AIM) theory^25^ provides insight into the effects of the solvent environment on the flexibility of bonds between π-conjugated moieties. Defined in terms of the cylindrical asymmetry of ρ(**r**) around the axis connecting two atoms, the ellipticity (ϵ) of a bond reveals the extent of π-conjugation along it.^25^ A single σ-bond has a symmetric ρ(**r**) distribution and ϵ = 0. Adding a single π-bond between the atoms increases ϵ. Our results show a larger electricity of N^I^–C^2^ than that of the C^II^–C^1^ bonds (Figure 2c,d), where the superscripts indicate the atomic positions (Figure 2a). That is, π-conjugation along the *N*-terminal N^I^–C^2^ bonds is considerably more pronounced than that along the *C*-terminal C^II^–C^1^ ones. The asymmetric ϵ along the N^I^–C^2^ bond indicates polarization with enhancement of electron density at the nitrogen. Conversely, the C^II^–C^1^ bond is not as polar as the N^I^–C^2^ bond. Furthermore, an increase in solvent polarity decreases the ellipticity of N^I^–C^2^ and C^II^–C^1^ (Figure S3), which is consistent with reducing the partial double-bond character between the amides and the aromatic rings, favoring enhanced twisting between the residues.

The difference between the π-bond character of N^I^–C^2^ and C^II^–C^1^, as revealed by their ellipticities, reflects the larger twists of the *C*-terminal than the *N*-terminal amides off the planes of the aromatic rings, i.e., |φ| < |ϕ| (Figure 2a and Table S9). This increased deviation of φ from 180° concurs with the weakened bonds between the carbonyls and the aromatic rings, which is consistent with the π_nb_-orbital nodes through the amide carbons.^26^ Conversely, the enhanced electron density on the amide nitrogens strengthens π-conjugation with the aromatic rings, keeping ϕ closer to 180° than φ. Empirical characteristics, such as the Swain and Lupton resonance (*R*_SL_) and field (*F*_SL_) parameters, accounting for π-conjugation and inductive effects of aromatic substituents, respectively,^27^ reflect well this difference between the bonding patterns of amide substituents. Both *N*-acylamides and *C*-acylamides exert electron-withdrawing inductive effects with *F*_SL_ ≈ 0.3. Nevertheless, *N*-acylamides are mesomerically electron-donating with *R*_SL_ ≈ −0.3, while *R*_SL_ ≈ 0 for *C*-acylamides suggests negligible π-conjugation.^27^

The HBs between the amides and their π-conjugation with aromatic rings counter the steric hindrance with hydrogens 3 and 6, favoring planarity of the AA oligomers. Nonetheless, these structures are not truly planar. The dihedral angles between the amides and the aromatic rings deviate from 180° by less than 30° (Table S9). These relatively small deviations, however, do not appear to compromise the extended conformation of short AA oligomers of less than about 5 or 10 residues (Figure S4a,b), as X-ray crystallography and NMR analysis reveal.^17,28^ Adding the multiple deviations of ϕ and φ from 180° upon expansion of the oligomer length beyond 10 residues, however, leads to the emergence of curvatures in the AA backbones (Figure S4c,d). The electret macrodipoles rely on codirectional alignment of the polar functional groups, such as the amides, along the oligomer backbones. Structural deviations from linearity of the AA conjugates thus impact the magnitude of their macrodipoles.

### Macrodipoles of the AA Electrets

**Figure 3:**
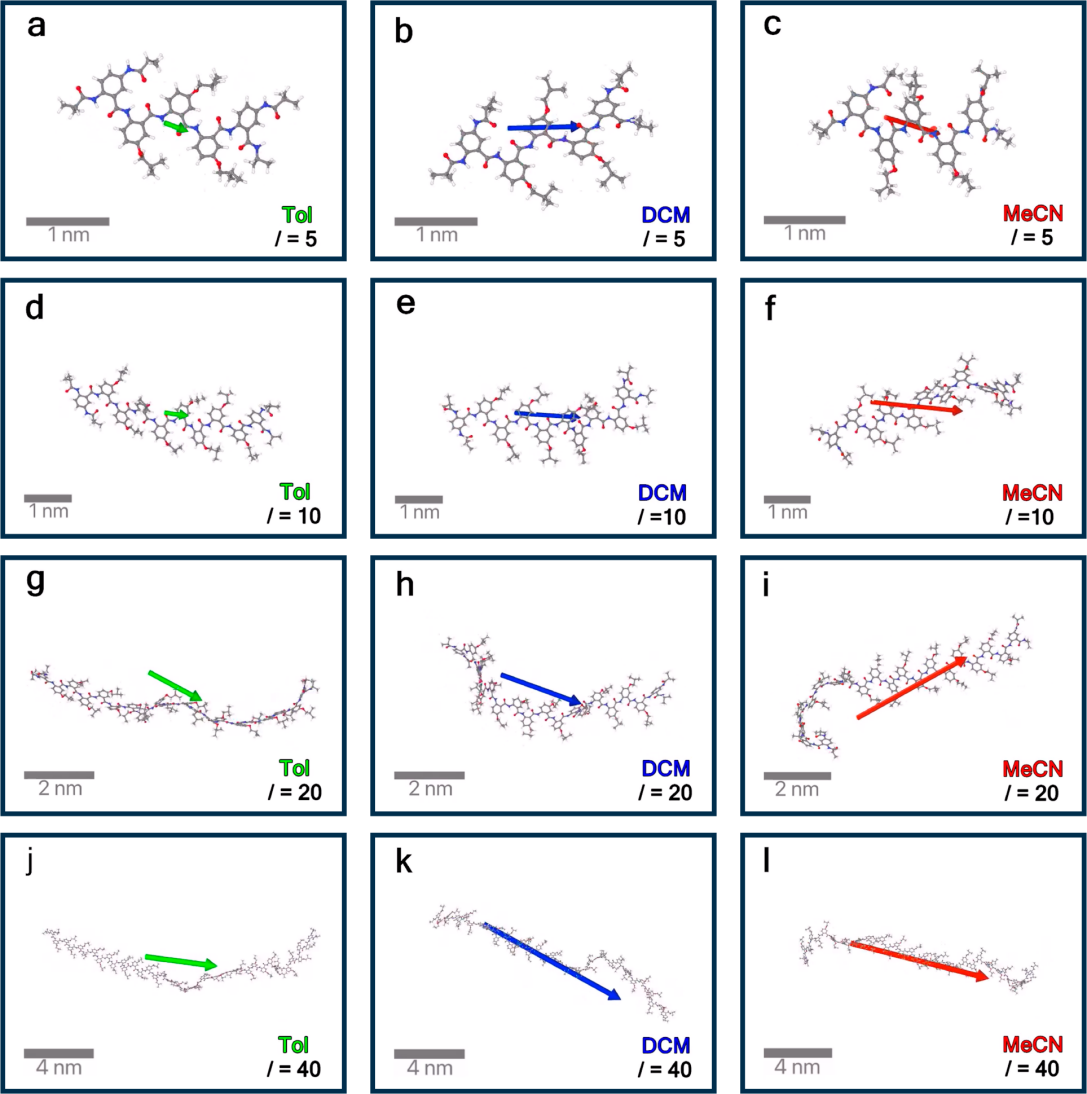
Representative frames from trajectories of 1-ns PQEq-MD simulations of Aaa-(Box)_*l*−2_-Aaa, along with their electric macrodipoles, in different explicitly introduced solvents. The number of residues, *l*, varies from 5 to 40, i.e., (a–c) *l* = 5; (d–f) *l* = 10; (g–i) *l* = 20; and (j–l) *l* = 40. (a, d, g, j) For toluene, green arrows represent the macrodipoles; (b, e, h, k) for dichloromethane, blue arrows; and (c, f, i, l) for acetonitrile, red arrows. The solvent molecules are hidden for improved visualization of the conformational dynamics of the electret oligomers. Movies showing the 1-ns trajectories timed to be the same length with still images (pausing) at the end are available in the HTML version of the article for (a) Tol, *l* = 5; (b) DCM, *l* = 5; (c) MeCN, *l* = 5; (d) Tol, *l* = 10; (e) DCM, *l* = 10; (f) MeCN, *l* = 10; (g) Tol, *l* = 20; (h) DCM, *l* = 20; (i) MeCN, *l* = 20; (j) Tol, *l* = 40; (k) DCM, *l* = 40; and (l) MeCN, *l* = 50.

**Figure 4:**
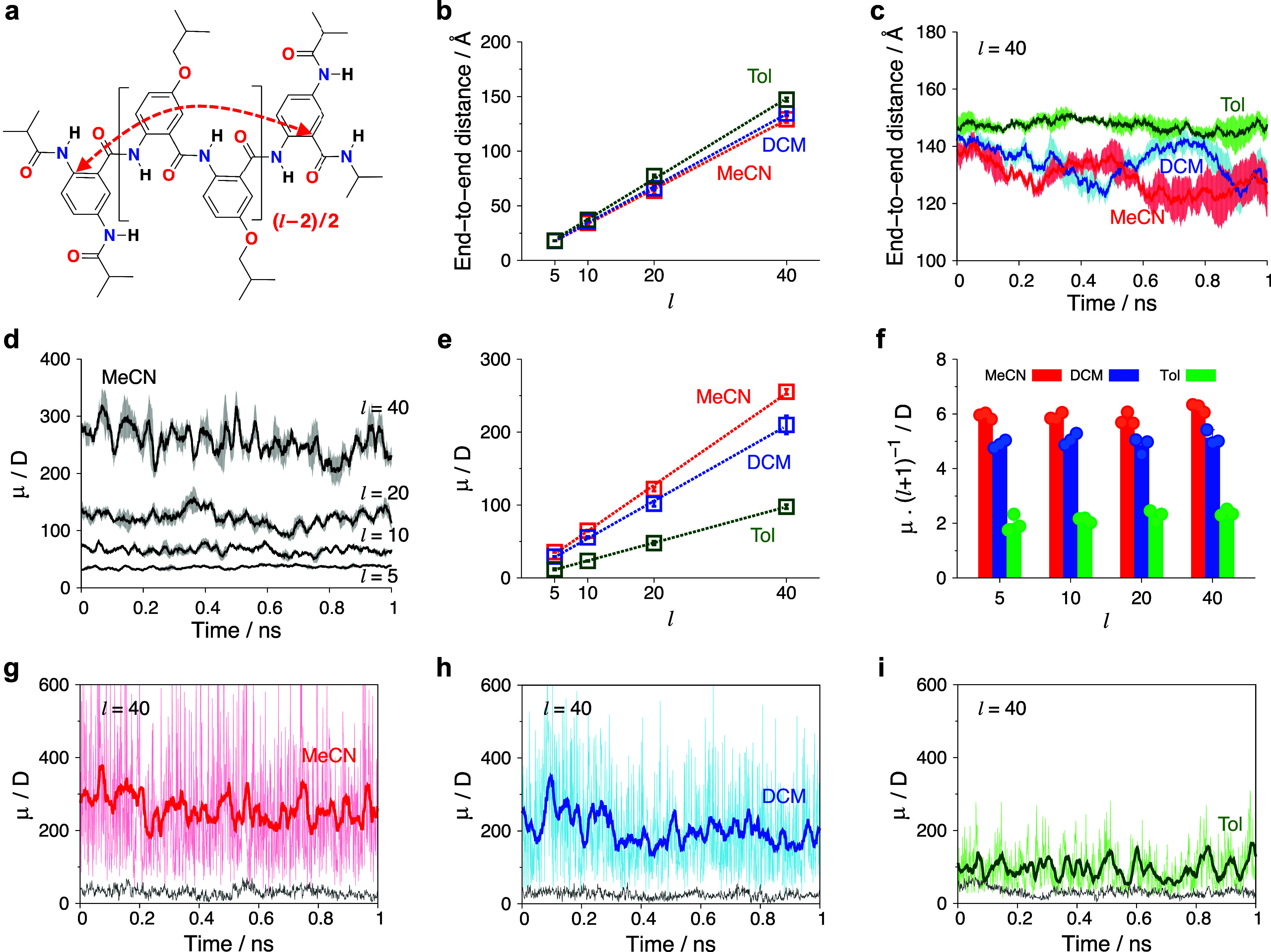
Structural and dipole analyses of Box AA oligomers with different lengths from 1-ns MD simulations. (a) Chemical structure of the Aaa-capped Box oligomer, where we consider residue lengths of *l* = 5, 10, 20, and 40. (b) Average end-to-end distances for the AA oligomers (as indicated by a red arrow in panel a) in three solvents with differing polarity: MeCN, DCM, and Tol. (c) Average end-to-end distances for the AA oligomers (*l* = 40) in three solvents over time, where shaded areas represent standard error from three replicas. (d) Average dipoles for the AA oligomers in MeCN, where the average was calculated from moving averages of three replicas with a window size of 20 ps and the shaded areas represent standard error. (e) Calculated dipoles in the three solvents with different polarity. (f) Dipoles per residue, estimated from the total dipoles divided by *l* + 1, which is the number of backbone amides. Dipole fluctuations of the AA oligomer (*l* = 40) in (g) MeCN, (h) DCM, (i) and Tol solvents over time, where the thin pink, sky-blue, and light-green lines show the dipole of the AA oligomer at each picosecond, and the thick red, blue, and green lines indicate moving averages with a window size of 20 ps. The gray lines show the dipoles of the AA oligomers in the gas phase calculated by removing solvents from the trajectories.

Although the DFT results are informative, they describe single optimized structures in implicit solvents as continuum media characterized by dielectric constants. To elucidate the dynamics of the AA oligomers immersed in explicit solvents with defined molecular structures, we perform MD simulations for Aaa-Box_*l*–2_-Aaa oligomers with *l* = 5, 10, 20, and 40 (Figures 3, 4a, and S4).

In order to accurately describe the dynamic behavior of these molecular structures, including the dynamics of atomic charge fluctuations and polarization, we implement (1) modified UFF^22^ for the bonded interactions combined with (2) PQEq (electrostatics),^20^ UNB (vdW),^21^ and HB^29^ for nonbonded interactions. We validate this computational methodology with MD simulations of small aliphatic amides that we previously studied employing NMR, impedance spectroscopy, and DFT calculations.^30^ The MD simulations reproduce the results from the experimental and DFT analyses (see Supporting Information), giving us confidence in applying this methodology to exploring the structural dynamics of other amide conjugates, such as AA electrets.

The 1-ns MD simulations show an overall extended conformation of the AA oligomers, even for the longest structure, with 40 residues (Figures 3 and S5). The average end-to-end distances increase linearly with increasing oligomer length (Figure 4a,b). For the nonpolar solvent Tol, the AA oligomers exhibit the longest end-to-end distances, which agrees with the DFT result that nonpolar solvents enhance the planarity of the residues. In comparison, the polar solvents MeCN and DCM shorten the average end-to-end distances of the AA oligomers, particularly for the long oligomers (Figures 4b,c and S6). The fluctuations of the end-to-end distance of the pentamer do not exceed 15% (Figures 3a–c and S5a). For the oligomers with *l* ≥ 10, temporary formation of bends along their backbones emerges, which is more pronounced for DCM and MeCN than for toluene (Figure 3d–i). The amplitudes of end-to-end distance fluctuations of the 20-mer and 40-mer increase from about 10 to 30 Å when the oligomers are transferred from Tol to MeCN (Figure 4c). These temporary bends are over several residues and do not lead to π–π-stacking interactions between the aromatic moieties (Figure 3).

The planarity of the AA oligomers results from HB interactions between the amides at each residue along the backbone and their π-conjugation with the aromatic rings. The calculated HB energy is about 0.2 eV per residue for Tol and ∼10% smaller for DCM and MeCN, with little dependence on oligomer length (Figure S7). As mentioned above, the AA oligomers intrinsically exhibit slightly twisted dihedral angles due to steric hindrance between the amide backbone and the aromatic ring. The MD simulations show a higher rigidity for the ϕ dihedral angles compared with φ, which agrees with the DFT findings (Figure S8).

Amides possess sizable intrinsic permanent electric dipoles.^30^ Hence, molecules with long backbones containing codirectionally ordered amides should exhibit large dipole moments. Indeed, the calculated dipoles from the MD simulations show a linear increase with the length of the oligomers for all solvent (Figures 4d,e and S9). That is, the average magnitude of the macrodipole is proportional to the number of residues in the oligomer. The dipole magnitude, however, depends significantly on the polarity of the solvents, i.e., |**μ**_MeCN_| > |**μ**_DCM_| > |**μ**_Tol_|, and the predicted dipoles per residue are about 6.0 D for MeCN, 5.0 D for DCM, and 2.2 D for Tol (Figure 4e,f). This trend is consistent with the Onsager reaction field that polar media induces in the solvation cavity.^30,31^ For the explicit solvent description, the medium polarization involves (1) alignment of the polar solvent molecules along the localized electric fields generated by the solute dipoles, i.e., orientational polarization leading to electrofreeze,^32^ and (2) shifts in the nuclear coordinates and the electron density of the solvent molecules, i.e., vibrational and electronic polarizations, respectively. Entropic randomization of the solvent balances the electrofreeze from prevailing as the distance from the solvation cavity increases.

Our MD simulations reveal an intriguing dynamic phenomenon: the macrodipoles exhibit large rapid fluctuations which intensify with increasing solvent polarity and oligomer length (Figures 3, 4g–i, and S10). These fluctuations are considerably more drastic than the structural dynamics illustrated by the variations in end-to-end distance of the AA oligomers (Figures S6 and S10). This finding suggests that transient arrangements of solvent molecules surrounding the AA oligomer play a pivotal role in generating these huge, short-lived transient dipoles. It is worth noting that the estimated dipoles of the AA oligomer without the solvents remain small and exhibit minimal fluctuations regardless of the solvent polarity (gray lines in Figures 4g–i and S10). The dipole of the DFT-optimized pentamer also shows contributions from the implicit solvents, but not as large as those from the MD analysis (Table S10). The MD simulations reveal both substantial contributions from the explicitly described solvents to the dipoles of the solvated oligomers and the emergence of transient macrodipoles with large magnitudes which cannot be explained solely by the ordered arrangement of the functional groups of the AA conjugates. These findings are consistent with fluctuations of the medium-induced Onsager reaction field in the solvation cavities.

### Is HB with the Solvation Media Important?

**Figure 5:**
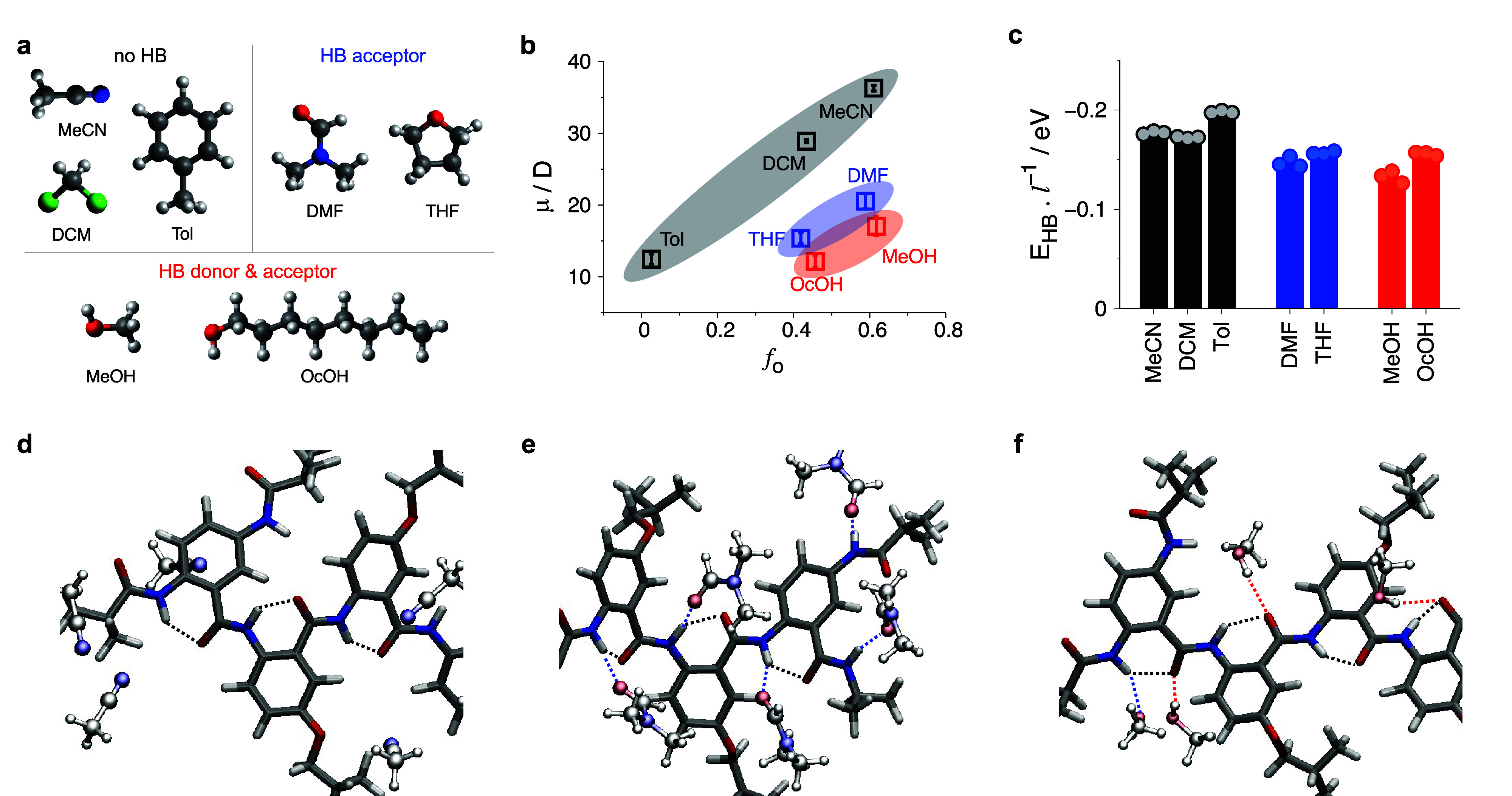
Effects of solvents with different HB capabilities revealed by MD calculations. (a) Structures of the seven solvents used in this study. (b) Calculated average dipole moments of Aaa-Box3-Aaa as a function of Onsager solvent polarity (*f*_0_):^33^*f*_0_(*x*) = 2(*x* – 1)(2*x* + 1)^−1^ and *f*_0_ = *f*_0_(ε) – *f*_0_(*n*^2^), where ε is the relative static dielectric constant and *n*^2^, i.e., the square of the refractive index, represents the dynamic dielectric constant at optical frequencies.^34^ (c) Estimated intramolecular HB energy, *E*_HB_, per residue. Representative HB interactions between the electret molecule and solvent molecules for (d) MeCN, (e) DCM, and (f) MeOH. Black (intramolecular HB), blue (HB acceptor), and red (HB donor) dotted lines show the different types of HB interactions, and gray, white, red, and blue represent carbon, hydrogen, oxygen, and nitrogen atoms, respectively.

The results showing enhancement of the macrodipoles with increasing medium polarity and oligomer length are for solvents that lack specific intermolecular interactions with the AA conjugates (Figure 4). The power of MD simulation to introduce solvents explicitly provides the means for exploring the effects of specific intermolecular interactions, such as HB, on the properties of the solvated oligomers. To examine how HB between the AA electrets and the solvent affects the structural integrity of these oligomers, we resort to MD simulations on the pentamer, Aaa-Box_3_-Aaa (due to its conformational stability, Figures 3a–c and S5a), with various solvents capable of HB interactions, i.e., dimethylformamide (DMF), tetrahydrofuran (THF), methanol (MeOH), and 1-octanol (OcOH) (Figure 5a). DMF is a broadly used organic solvent with polarity similar to that of MeCN, and THF has polarity similar to that of DCM. These two solvents are HB acceptors but not HB donors. While MeOH and OcOH have polarities similar to those of MeCN and DCM, respectively, they can act as both HB donors and acceptors.

The 1-ns MD simulations for Aaa-Box_3_-Aaa in solvents that form HBs do not show breaks in its intramolecular HB network. Nevertheless, the AA dipole shows a dependence on the HB capability of the solvents, even though the fundamental trend remains the same; i.e., an increase in solvent polarity increases the oligomer macrodipole (Figure 5b). The oligomer in DMF and THF, which are only HB acceptors, exhibits significantly lower dipoles compared to those in MeCN and DCM, despite the similar solvent polarities. When placed in MeOH and OcOH that act as both HB donors and acceptors, the AA conjugate shows even smaller dipoles than when in DMF and THF. These results indicate that the HB capability of the solvent significantly affects the dipoles of the AA electrets. This finding is consistent with the trends of the estimated intramolecular HB energies of the AA oligomer, showing a decrease with enhanced HB capability of the solvent (Figures 5c and S15). Hence, HB interactions with the solvation media do not necessarily break the HB of the AA structures. Nevertheless, they weaken the intramolecular HB network by intermolecular HB interactions (Figure 5d–f).

These MD simulations also reveal that these solute–solvent HB interactions can induce conformational changes in the amide bond. Typically, the *trans* conformation of the amide is about 0.2 eV more stable than the *cis*.^35^ We observe, however, 8.1% *cis* amides for MeOH that is polar and acts as HB donor and acceptor (Figure S13). Furthermore, the HB solvents induce large fluctuations in the dihedral angles of the electret molecules (Figure S14). These results demonstrate that solvation media with HB propensity compromise the structural integrity and the macrodipoles of molecular electret systems.

### Do the Side Chains of the AA Electrets Matter?

Although the ordered amide orientation and the HB network along the backbone of the electrets are principally responsible for maintaining their extended conformation and macrodipoles, the side chain substituents, i.e., R_1_ and R_2_ at positions 4 and 5 (Figures 1 and 2a), also affect the AA properties. For example, not only the type of substituent but also its position, i.e., 4 vs 5, affect the electrochemical potentials of the AA residues and their susceptibility to oxidative degradation.^14^

**Figure 6:**
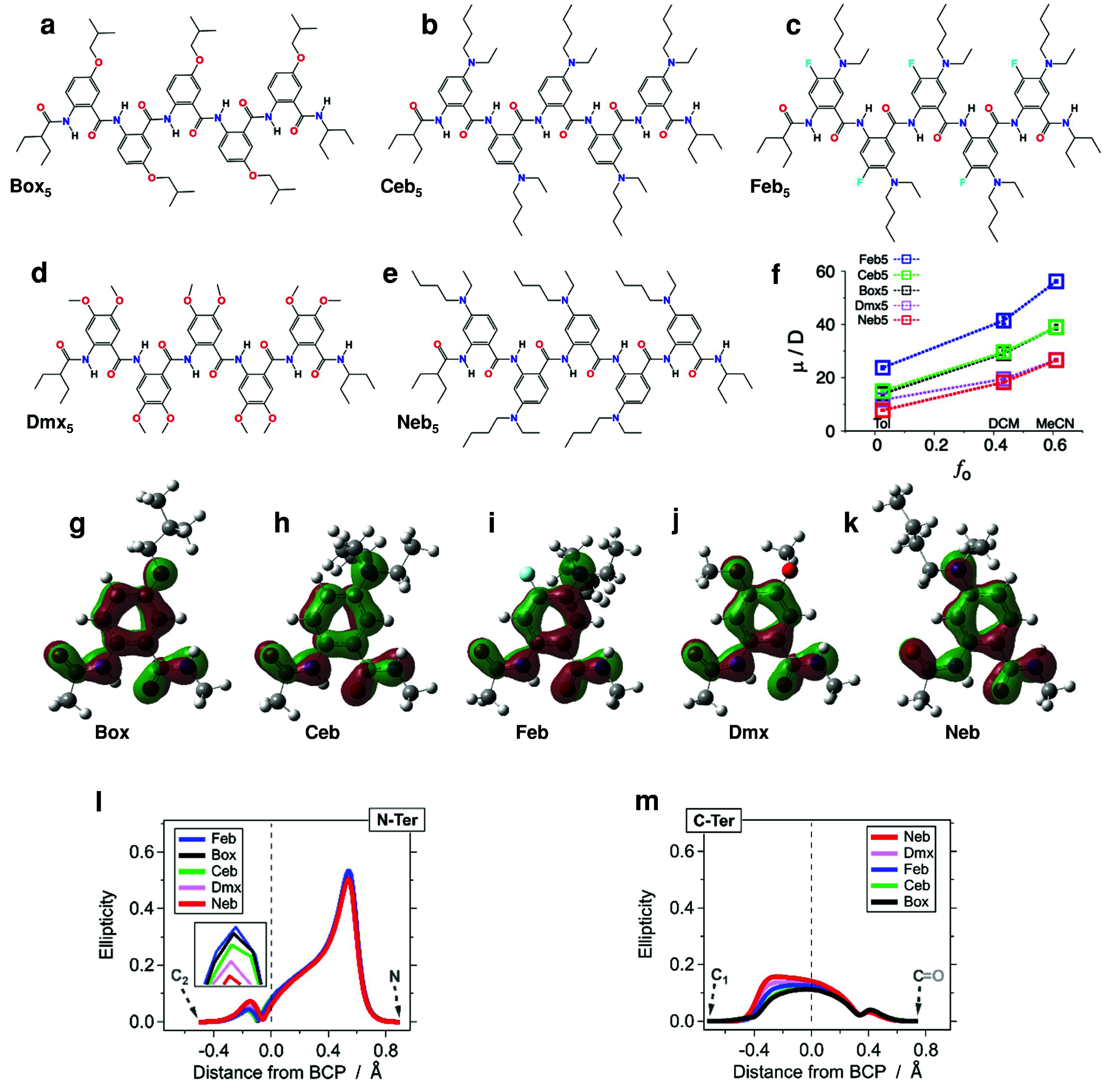
Effects of side chains of the AA electret residues. (a–e)Chemical structures of the AA pentamers for *l* = 5, with different side chains. (f) Calculated average dipole moments as a function of Onsager solvent polarity for three solvents from 1-ns MD simulations. (g–k) Localized π-orbitals of the monomeric residues of each of the pentamers, obtained from DFT calculations for the gas phase (isovalue: 0.08). Bond ellipticities of the (l) *N*-terminus and (m) *C*-terminus amides to the phenyl ring of the five oligomers from DFT calculations for the gas phase.

To examine the effects of the side chains on the macrodipoles, we perform DFT calculations and MD simulations on AA pentamers, each composed of the same electron-rich residue with various R_1_ and R_2_ substituents (Figure 6a–e). Placing an electron-donating substituent as R_2_ at position 5, such as in Box and Ceb, polarizes the aromatic rings in the direction of the macrodipole of the AA electrets, which point from their *N*- to their *C*-termini (Figure 1). That is, electron-donating R_2_ groups enhance the electret macrodipoles. Attaching an electronegative substituent as R_1_ at position 4, such as fluorine in Feb,^36^ further enhances this polarization of the aromatic ring The MD simulations, indeed, show average macrodipoles of Feb_5_ in solvents with various polarities that are substantially larger than those of Ceb_5_ and Box_5_ (Figure 6f).

Conversely, electron-donating R_1_ groups exert an opposite polarization effect, and the macrodipole of Neb_5_ is the smallest for all solvents (Figure 6f). In Dmx, these effects from the two ethers, R_1_ and R_2_, should cancel each other. Nevertheless, the average dipole of Dmx_5_ in Tol is similar to that of Box_5_ (Figure 6f). The solvent polarity, therefore, affects to different extents the polarization that side chains R_1_ and R_2_ induce on the aromatic residues.

Counterintuitively, our results show that it is the position of the substituents, rather than their electron-donating capability, that affects the electret macrodipole. Despite the difference between the electron-donating strengths of amines and alkyloxyls^27^ and the drastically different potentials for oxidizing the residues that contain them,^14^ their effect on the electret macrodipoles are quite similar, as the overlapping trends for Box_5_ and Ceb_5_ reveal (Figure 6f).

Bond ellipticity analysis from DFT calculations of the residues with different side chains elucidates the effects of the R_1_ and R_2_ substituents on the backbone structure and concurs with the MD findings about the oligomer macrodipoles. Electron-donating substituents at the R_2_ position para to the N^I^–C^2^ bond increase its ellipticity, as in Feb, Box, and Ceb (Figure 6l). Conversely, using an electron-donating R_1_ group at the *meta* position, as in Neb, redirects the electron density and lowers the N^I^–C^1^ ellipticity.

The side chains have a stronger effect on the C^II^–C^1^ bonds than the N^I^–C^2^ bonds between the amides and the aromatic rings (Figure 6g–m). Electron-donating R_2_ substituents at the position *meta* to the *C*-terminal amides reduce the π-character of the C^II^–C^1^ bonds, as in Box and Ceb (Figure 6g,h,m). Conversely, an electron-donating R1, i.e., *para* to C^1^, not only enhances the π-character of the C^II^–C^1^ bonds but also increases its asymmetry, with electron density drawn toward the aromatic ring, as in Neb (Figure 6k,m). With two identical electron-donating substituents, the C^II^–C^1^ bond of Dmx is similar to that of Neb rather than Box (Figure 6j,m), indicating that the R_1_ group *para* to C^1^ has a stronger effect on the π-character of the C^II^–C^1^ bond than the *meta* R_2_ side chain. While strongly electron-withdrawing along the σ-skeleton, fluorine is slightly electron-donating along the π-bonds,^37^ and placing it as R_1_ next to an R_2_ amine indicates some increase in ellipticity and asymmetry of the C^II^–C^1^ bond of Feb (Figure 6i,m).

The side chains affect the polarization and rigidity of the bonds between the backbone amides and the aromatic moieties. An increase in the double-bond C^II^–C^1^ character, as well as pulling the π-electron density toward the aromatic ring along C^II^–C^1^ and N^I^–C^2^ bonds, correlates with decreasing the electret macrodipoles.

### Do the Macrodipoles Matter?

The short answer is “yes, they do”. Nevertheless, the inherently strong nature of electrostatic interactions warrants revisiting this question. Polar molecules, indeed, tend to have a propensity for aggregating with opposing orientations of their dipoles. The cancellation of the macrodipoles in such aggregates appears to question the need to pursue and optimize the designs of electret structures. Even AA molecular electrets without side chains R_1_ and R_2_—needed for improving solubility and suppressing π-stacking—form aggregates exhibiting macrodipole cancelation.^28^

Conversely, numerous examples demonstrate the need for macromolecular structures with large electric dipole moments. Technologies employing liquid crystals, comprising assemblies of linear polar molecular structures that improve their order under external electric fields, are an inherent component of everyday life.^38,39^ Macrodipoles strongly affect CT thermodynamics and kinetics and can play crucial roles in enhancing the rates of desired processes while suppressing undesired ones.^3,40^ The intrinsic dipoles of protein α-helices are responsible for the functioning of transmembrane ion channels that maintain living cells alive.^6,41^

With macrodipoles reaching 5 D per residue, polypeptide helices are among the most polar linear molecular structures known.^12^ The amino acids sequence can control the state of aggregation of these biomolecules,^42−44^ and designed polypeptide helices without propensity for aggregation at sub-millimolar concentrations have allowed demonstrating dipole effects on CT kinetics.^7,45,46^

Interfacing such macromolecular electrets with solid conductors and semiconductors is essential for device designs and technology developments.^3^ The amino acid side chains and the method of self-assembly govern the structures of monolayers of polypeptide α-helices formed at liquid–air interfaces or physisorbed on metal surfaces.^47^ Resorting to strong chemisorption involving, for example, the formation of sulfur–gold bonds, along with sequence designs favoring codirectional orientation, allows self-assembly of polypeptide α-helices on conductive surfaces with their dipoles pointing in the same direction, to or from the solid substrate. The codirectionally oriented dipoles of such self-assembled monolayers of polypeptide helices induce rectification of photocurrents and charge transport.^8,48^

In addition to the advances that demonstrate the importance of molecular electrets, however, it is important to consider the implementation of their macrodipoles. The orientation of polypeptide α-helices can appear to have no effect on CT between charged electron donors and acceptors attached to them.^49^ The counterions of the charged moieties and polar solvating media screen dipole-generated fields and suppress or completely eliminate their effects on CT.^15,16^

The polarization of the media around solvation cavities damps the localized fields originating from solvated dipoles. Concurrently, the same medium polarization enhances the magnitudes of such solvated dipoles. Dipole-generated fields force orientation, along with nuclear and electronic polarization, of the surrounding molecules of polar solvents. The dipoles and the induced dipoles of such polarized media generate a reaction electric field inside the cavity that is codirectional with the field of the solvated (macro)dipole. That is, an increase in medium polarity has two opposing effects on solvated dipoles: (1) it suppresses the propagation of the dipole-generated fields outside the solvation cavities, diminishing the dipole effects on the surrounding species, and (2) it enhances the magnitudes of the solvated dipoles by inducing Onsager reaction fields inside the solvation cavities.^31^ Increases not only in solvent polarity but also in solvent polarizability induce sizable enhancement of solvated dipoles, as impedance spectroscopy and QM calculations reveal.^30^

In addition to these two opposing solvent effects on molecular dipoles, an increase in the medium polarity compromises the planarity of the AA electrets. These multifaceted solvent effects on macrodipoles warrant careful approaches to not only the design but also the implementation of molecular electrets.

## Conclusions

The MD-PQEq methodology allows interrogating the structural dynamics and dipole properties of large polar systems, such as bioinspired molecular electrets with length exceeding 100 Å. Such MD simulations reveal unexpected external and internal effects on the dipole dynamics. Specific interactions with the solvents and polarization from the residue side chains strongly affect the oligomer macrodipoles. An increase in solvent polarity enhances not only the electret dipoles but also the amplitude of their fluctuations. Decreased rigidity of the oligomer backbones accenuates the latter, as DFT calculations demonstrate. When averaged over tens of picoseconds, the macrodipoles appear to be quite permanent. Corollary mostly to the solvent dynamics and the reaction-field fluctuations, however, the dipoles manifest huge picosecond transient jumps, making them not so permanent at such fast time scales. Therefore, such macromolecular dipoles should impact differently processes with different rates, providing key guidelines for implementing these bioinspired structures for crafting localized electric fields in charge-transfer and energy-conversion systems. Beyond the AA structures, our findings provide design principles for developing a class of organic materials with novel electronic properties. Furthermore, this study demonstrates the power of the PQEq-MD methodology, in synergy with QM calculations, for multifaceted characterization of the dynamic complexity of large dipolar systems in condensed media.

## Experimental Section

### MD Simulations

All MD simulations were performed using the RexPoN-integrated version of the LAMMPS^21,50^ molecular dynamics package. The time step was set to 1 fs, and a Nosé–Hoover thermostat (100 fs damping constant) was employed for NVT (constant particles, volume, and temperature) simulations. After minimization, the systems were first heated from 10 to 300 K over 100 ps. Next, NVT simulations were performed for 1 ns at 300 K. We used the modified universal force field (UFF)^22^ for the bonded interactions and PQEq (electrostatic),^20^ UNB (vdW),^21^ and UHB (HB) for nonbonded interactions. More detailed information is provided in the Supporting Information.

The electret oligomers were placed in 40 × 30 × 30 Å^3^, 70 × 40 × 40 Å^3^, 100 × 53 × 53 Å^3^, and 165 × 53 × 53 Å^3^ boxes for residue lengths *l* = 5, 10, 20, and 40, respectively. The solvent molecules were placed within each box to match the experimental densities: 1.03 (DO), 1.48 (Chl), 1.33 (DCM), 0.79 (MeCN), 0.86 (Tol), 0.94 (DMF), 0.88 (THF), 0.79 (MeOH), and 0.83 (OcOH) g cm^–3^.^51^ For each system, we performed three independent MD simulations with different initial structures (*n* = 3 runs) and calculated averages with standard errors from the three replicates. All initial structures were generated by packmol,^52^ while VMD^53^ was used for visualization and analysis of the MD trajectories. Movies were generated by OVITO.^54^

### Density Functional Theory (DFT) Calculations

We used the B3LYP functional within the DFT framework along with the Grimme dispersion DFT-D3 correction.^55^ We employed the 6-31G(d) basis set.^56,57^ Our convergence criteria were 10^–4^ au for the average residual forces for geometry optimization and 10^–8^ au for the self-consistent field energy. Solvation effects were included using the integral equation formalism variant of the polarizable continuum model (IEFPCM).^58^ All molecular structures of the monomers and pentamers were optimized using the Gaussian 09 program package.^59^

For Box oligomer calculations, we truncated the alkyl chains to methyl groups, as the conformations of the flexible alkyl chains were often improperly optimized, trapping the entire structure in a local minimum.

## Supporting Information Available

Supplementary computational results and simulation details (PDF)

## Notes

An animated version of the TOC graphic showing a sequence of the MD trajectories of short and long molecular electrets in three solvents with different polarities and movies showing the 1-ns trajectories represented in Figure 3 are available as video files (AVI) in the HTML version of the paper.
