# Supplementary material for: How Permanent Are the Permanent Macrodipoles of Anthranilamide Bioinspired Molecular Electrets?
Source: J Am Chem Soc. 2024 Jan 16;146(8):5162–72. doi: 10.1021/jacs.3c10525 (PMC10916682; doi:10.1021/jacs.3c10525)
Supplement: Supplementary file 1 — ja3c10525_si_001.pdf [file ja3c10525_si_001.pdf]

How Permanent are the Permanent Macrodipoles of Anthranilamide Bioinspired Molecular Electrets?

Supporting Information

Moon Young Yang[#,‡], *Omar O'Mari*[¶,‡], *William A. Goddard III*[#,*], *Valentine I. Vullev*[¶,±,$,§,*]

[#]Materials and Process Simulation Center, California Institute of Technology, Pasadena, California 91125, United States

[¶]Department of Bioengineering, University of California, Riverside, California 92521, United States

[±]Department of Chemistry, University of California, Riverside, California 92521, United States

[$]Department of Biochemistry, University of California, Riverside, California 92521, United States

[§]Materials Science and Engineering Program, University of California, Riverside, California 92521, United States

[*]To whom correspondence should be addressed. E-mail: wag@caltech.edu or vullev@ucr.edu

[‡]These authors contributed equally.

This PDF file includes:

Computational details

Supplementary Figures S1 to S15

Supplementary Tables S1 to S10

XYZ for optimized structures in DFT

Supplementary References

Computational Details

Dipole calculation. The dipole moments of the electret molecules were calculated at different levels of theory as follows.

i) UFF/QEq: The UFF is constructed based on atomic parameters and their combinatorial rules. The dipoles are calculated using atomic partial charges obtained from employing the charge equilibration (QEq) method. The QEq uses Gaussian shaped charges the size of the atom (not point charges) so that shielding of charges on adjacent atoms is properly described. QEq can be viewed as a semi-empirical (orbital-free) DFT, where the partial charges computed for the atoms are derived from the geometry as inputs with three key parameters for the isolated systems: ionization potential, electron affinity, and atomic radius. The first two are assumed not to vary between the bonded and non-bonded atoms. Hence, the dipole moment is calculated as point charges based on these parameters.

ii) HF/STO-3G: In the *ab initio* Hartree-Fock (HF) method, the Born-Oppenheimer approximation allows the dipole moments to be calculated from the charges of the nuclei using their fixed spatial positions, and from electron-density distribution according to the Slater-Condon rules.

iii) AM1: In semi-empirical methods, such as in AM1, there is an assumption of a zero-differential overlap. It neglects the diatomic differential overlap and employs the valence-only minimal basis, disregarding many of the two-electron integrals. Therefore, the charges represent the *net electric charges*, and the dipole moment is derived from the atomic charges only.

iv) DFT (B3LYP-D3): In DFT, the dipole moments are defined as the first moment of the total electric charge density. Usually, the first moment and the electronic density can be approximated as concentrated at individual atomic sites where each atom has an associated partial charge resulting from the difference between the nuclear charge and the portioned electron density. Thus, the approximated total charge density can give the total dipole moment as the sum of point charges for the electroneutral system.

v) UFF/PQEq: The PQEq method predicts dynamically the atomic charges and polarization underlying the electrostatic interactions, where the molecular dipole moment by this method is calculated using distributed PQEq (Gaussian) charges within a molecule. Detailed information about this method is provided in the following subsection.

Electrostatic Interactions. For an accurate description of dynamic electrostatic and polarization interactions, we developed the polarizable charge equilibration (PQEq) model.[1] PQEq uses an atomic-sized Gaussian-shaped electron density that can polarize away from the atomic-sized Gaussian-shaped core in response to internal and external electric fields. The atomic charges are updated dynamically each fs to achieve a constant chemical potential across all atoms in the system. Each atom is considered to have (1) a core including all the mass of the atom and (2) a massless electron shell. The core and shell are described by 1s Gaussian-shaped electron densities (ρ) distributed over a finite region based on the size of each atom. This includes the dynamic impact of dynamic polarization ignored in the standard fixed point charge models used by most FFs. The core has a variable total charge (q_i) and a fixed total charge ($+Z_i$) part, where the shell only has a fixed total charge of $-Z_i$. The dynamic position of the shell is determined by balancing the external forces coming from other atoms and applied fields with the spring force constant (K_s) that connects the shell and core of the atom. The electrostatic interaction energy between two Gaussian charges, the electron densities (ρ) of atoms i and j, is given by

$$C_{ik,jl}(r) = \frac{1}{r}\,\mathrm{erf}\left(\sqrt{\frac{a_{ik}a_{jl}}{a_{ik}+a_{jl}}}\,r\right)$$

where k and l denote the core (c) and shell (s), respectively. The width of the Gaussian distribution (α_{ik} and α_{ik}) is equal to $0.2341/R^2$, where R is the core (R_c) or shell (R_s) radius. Then, the electrostatic energy (E_{elect}) is defined by

$$E_{elect}(\{r_{ic}, r_{is}, q_i\}) = \sum_{i}^{N}\left[\chi_i^0 q_i + \frac{1}{2}J_{ii}^0 q_i^2 + \frac{1}{2}K_s r_{ic,is}^2\right] + \sum_{ik>jl} T(r_{ik,jl})\, C_{ik,jl}(r_{ik,jl}) q_{ik,jl}$$

where χ_i^0 is the Mulliken electronegativity (χ_i^0 = (IP + EA)/2), J_{ii}^0 is the idempotential or hardness (J_{ii}^0 = IP – EA), and $r_{ik,jl}$ is the interatomic distance. Here, IP and EA are averaged atomic ionization potential and atomic electron affinity, respectively. *T(r)* is the 7th order taper function that makes the potential energy go smoothly to zero at a finite distance. The second sum computes the electrostatic energy between the core and shells of all atoms in the system. The charge on the core (q_i) is updated every time step *via* efficient schemes. The complete description and validation of the PQEq model are provided in previous publications,[1–3] where we used the same PQEq1 parameters (H, C, N, O, F) from the original paper for this study.[1]

Table S1. The PQEq parameters, χ_i^0, J_{ii}^0, R_c (R_s), and K_s, used in this study.

Atom	χ_i^0 (eV)	J_{ii}^0 (eV)	$R_c = R_s$ (Å)	K_s (kcal mol^{-1} Å^{-2})
H	4.72484	15.57338	0.371	2037.20061
C	5.50813	9.81186	0.759	198.84054
N	7.78778	10.80315	0.715	301.87609
O	8.7410	13.3640	0.8028	814.0445
F	8.70340	17.27715	0.706	596.16463

For acetonitrile (MeCN) and dichloromethane (DCM) solvents, however, we reoptimized the PQEq parameters to ensure that the charge distribution on the molecule remains in a reasonable range. Since the original parameters for C-H-N and C-H-Cl systems were determined using $C_3H_3N_3$, $C_6H_{13}N$, C_6H_7N, and $C_5H_{12}N$, and $C_6H_{11}Cl$ molecules, respectively, they disagreed with the atomic charges from QM at the B3LYP/6-31(d,p) level. Thus, we reoptimized χ_i^0 values for C, N, and Cl in MeCN and DCM molecules, which now show good agreement with the QM results.

Table S2. The PQEq parameters, χ_i^0, J_{ii}^0, R_c (R_s), and K_s, for MeCN.

Atom	χ_i^0 (eV)	J_{ii}^0 (eV)	$R_c = R_s$ (Å)	K_s (kcal mol^{-1} Å^{-2})
H	4.72484	15.57338	0.371	2037.20061
C (sp^3)	6.70813	9.81186	0.759	198.84054
C (sp^1)	5.00813	9.81186	0.759	198.84054
N	8.28778	10.80315	0.715	301.87609

Table S3. The PQEq parameters, χ_i^0, J_{ii}^0, R_c (R_s), and K_s, for DCM.

Atom	χ_i^0 (eV)	J_{ii}^0 (eV)	$R_c = R_s$ (Å)	K_s (kcal mol^{-1} Å^{-2})
H	4.72484	15.57338	0.371	2037.20061
C	6.90813	9.81186	0.759	198.84054
Cl	7.00651	9.738908	0.994	152.32280

Table S4. The comparison of atomic charges of MeCN calculated by QM and FF.

	QM (B3LYP/6-31(d,p))		FF	
	Mulliken	ESP	original	this work
H	0.186	0.191	0.119	0.192
C (sp^3)	-0.379	-0.515	-0.036	-0.462
C (sp^1)	0.346	0.491	0.167	0.449
N	-0.524	-0.561	-0.496	-0.579

Table S5. The comparison of atomic charges of DCM calculated by QM and FF.

	QM (B3LYP/6-31(d,p))		FF	
	Mulliken	ESP	original	this work
H	0.226	0.272	0.215	0.269
C	-0.360	-0.390	0.239	-0.332
Cl	-0.046	-0.077	-0.352	-0.109

Nonbonded Interaction. Most FFs distinguish between short range valence interactions, *i.e.*, bonds, angles, torsion, and inversion terms, and long-range nonbonded interactions, *i.e.*, electrostatics, polarization, and van der Waals (vdW) terms (which account for London dispersion and Pauli repulsion). In addition, hydrogen bond (HB) corrections are often needed to describe short-range corrections to the nonbonded interactions. In our previous work, we obtained the pure 2-body vdW nonbonded potentials from the equation of state (EOS) for single element solid systems (H, C, N, O, F, Cl, Br, I, P, He, Ne, Ar, Kr, Xe, and Rn) using DFT with dispersion corrections.[3] We discovered that scaling the nonbonded potential curves of the noble gases by normalizing the well depth energy (D_e), bond distance (R_e), and curvature using a scaling length (L) led to nearly identical scaled potentials. This universal nonbonded potential requires only three parameters for each element, and the universal nonbonded potential (E_{UNB}) function is given by

$$E_{UNB}(r_{ij}) = -D_e \exp\left[-\beta\left(\frac{r_{ij}-R_e}{L}\right)\right] T(r_{ij}) \sum_{n=0}^{5} s_n \left(\frac{r_{ij}-R_e}{L}\right)^n$$

where r_{ij} is the interatomic distance, and β and α_n are the universal parameters. The $T(r_{ij})$ is a 7th order taper function with a finite range which is used to screen the nonbonded interactions. The detailed information is provided in our previous publication,[3] where we used the same UNB parameters for this study.

Table S6. The R_e, D_e, and L atomic parameters obtained from two-body universal nonbonded potential energy curves.

Atoms	R_e (Å)	D_e (kcal mol^{-1})	L (Å)
H	3.2541	0.0528	0.5241
C	3.9162	0.0971	0.5396
N	3.8281	0.1456	0.5416
O	3.4249	0.1498	0.4349
F	3.5018	0.1873	0.5199
Cl	4.0748	0.3500	0.5654

For MeCN and DCM solvents, however, we slightly optimized the R_e parameter because the changes in the PQEq parameters for MeCN and DCM solvents affected on the density.

Table S7. The R_e, D_e, and L atomic parameters obtained from two-body universal nonbonded potential energy curves.

Atoms	R_e (Å)	D_e (kcal mol^{-1})	L (Å)
H	3.2541	0.0528	0.5241
C (sp^3)	3.9162	0.0971	0.5396
C (sp^1)	3.5246	0.0971	0.5396
N	3.4453	0.1456	0.5416

Table S8. The R_e, D_e, and L atomic parameters obtained from two-body universal nonbonded potential energy curves.

Atoms	R_e (Å)	D_e (kcal mol^{-1})	L (Å)
H	3.2541	0.0528	0.5241
C	3.9162	0.0971	0.5396
Cl	3.4636	0.3500	0.5654

Hydrogen Bond Interaction. It is well known that biomolecules such as proteins need special HB corrections to account for the polar nature of the amide bonds. Thus, we considered an explicit term for the short-range HB interactions, where a Morse-type HB term as in Dreiding FF was used.[4]

$$E_{HB}(r) = D_{hb}\{\exp[-2\alpha_{hb}(r - r_{hb})] - 2\exp[-\alpha_{hb}(r - r_{hb})]\}\cos^{n_{hb}}\theta$$

where D_{hb}, α_{hb}, r_{hb}, and n_{hb} are the parameters to be determined. In our previous study, we determined one set of global HB parameters that can work well for any kind of HB interactions. The optimized parameters are D_{hb} = 8.0 kcal/mol, α_{hb} = 1.72, r_{hb} = 2.92 Å, and n_{hb} = 2, respectively, which were used for this study. More detailed information is provided in our previous publication.[3]

Method validation. To validate our computational methodology, we performed MD simulations for small aliphatic amides that we had previously studied employing NMR, impedance spectroscopy, and density functional theory (DFT) calculations.[5] Our focus here is on monoethylated (EHH and HHE), diethylated (EHE), and triethylated (EEE) amides in baths of explicitly described solvents: 1,4-dioxane (DO), chloroform (Chl), and dichloromethane (DCM) (Figure S1). The predicted dipoles for these aliphatic amides from the MD simulations are in good agreement with those estimated from impedance spectroscopy measurements and with those calculated from the DFT-optimized geometries using an implicit solvent representation. Since the π-delocalization expanding between the oxygen and the nitrogen of AA ensures a certain level of rigidity of *N*-acylamides, we find that the thermal fluctuations in experiment and in the MD simulations lead only to small deviations from the optimal planar geometries, as reflected by the similarities between the dipoles obtained from the three different methods.

In agreement with the other methods, the MD simulations show an increase in the estimated dipoles with an increase in the solvent polarity (Figure S1). This trend is consistent with the Onsager reaction field that polar media induces in the solvation cavity.[5–7] The Onsager solvation theory models dipolar species, such as amides with various complexity, as residing inside a cavity and exerting polarization on the surrounding solvent. These amides immersed in a dielectric continuum polarize the surrounding media. In turn, the polarized solvent induces an electric field within the cavity occupied by the solute. We find that extending

the formalism of this theory from a dielectric continuum to explicit solvent representation does not compromise its quantitative predictability.

This agreement of the MD results with the experimental and DFT findings gives us confidence in applying this methodology to exploring the structural dynamics of other amide conjugates, such as AA electrets. These MD simulations enable investigations of complex structures that are far too large for QM-based calculations, offering access to high-fidelity information that is readily lost in the ensemble-averaging inherent to "bulk" experimental techniques.

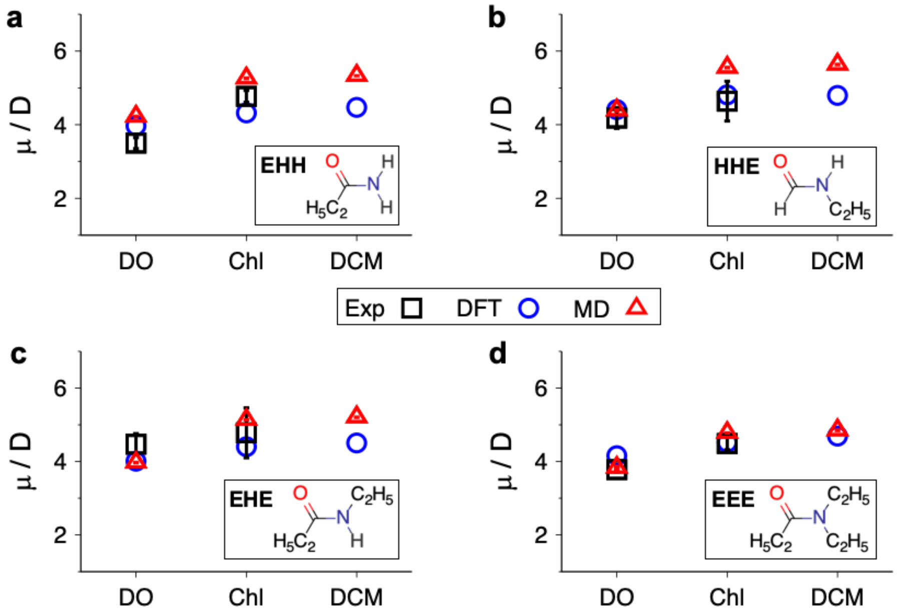

Figure S1. MD predicted electric dipole moments for aliphatic carboxyamides compared with previous experimental and DFT results.[5] The estimated electric dipole moments for (a) *C*-monoethylated, EHH, (b) *N*-monoethylated, HHE, (c) *C*,*N*-diethylated, EHE, and (d) triethylated, EEE, in three different solvents: DO, Chl, and DCM.

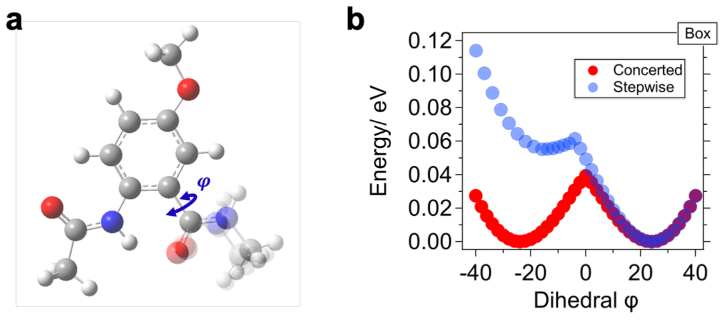

Figure S2. DFT energies of single-residue Box conformations. (a) Chemical structure of Box with the dihedral angle φ is changing across the benzene ring. (b) Gas phase - Concerted scan: the two amides at positions 1 and 2 are pointing to the same side of the aromatic ring and allowed to relax in concerted action vs. - Stepwise scan: dihedral φ is allowed to relax and flip in stepwise action while the N-terminus is held frozen.

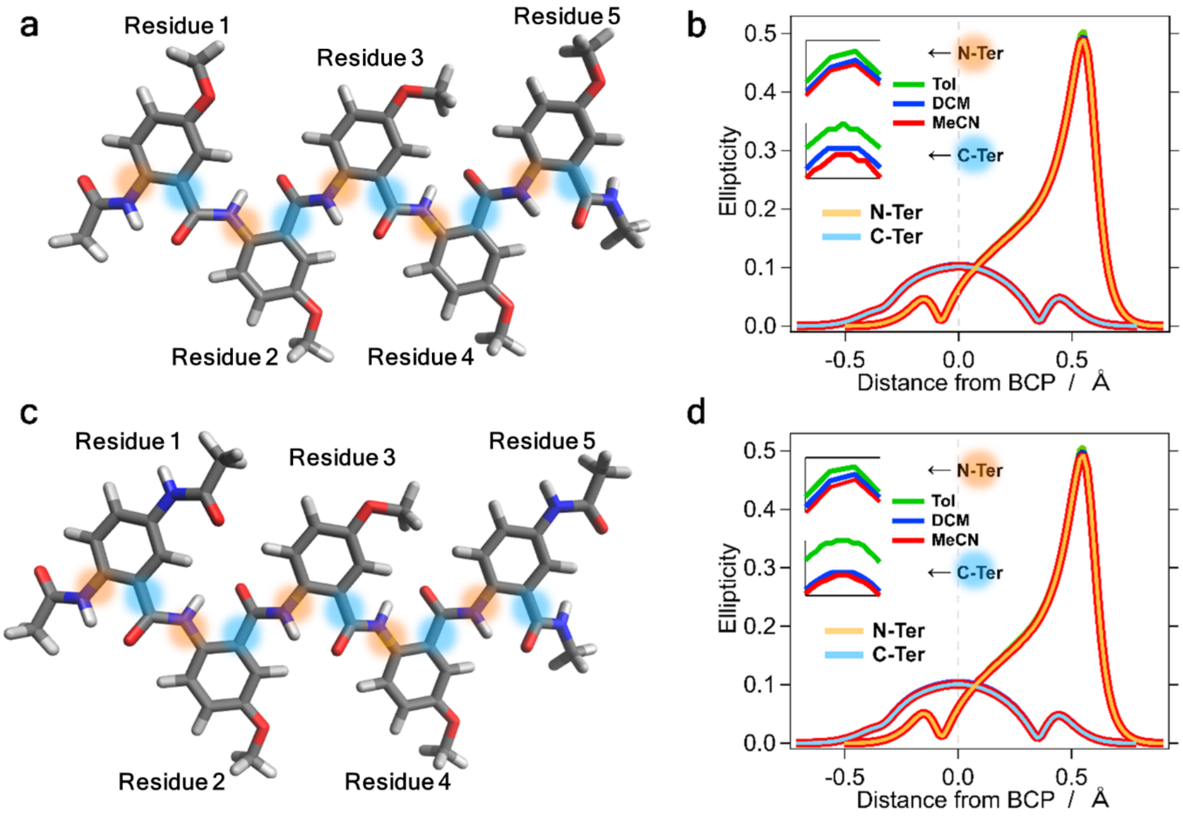

Figure S3. Solvent dependence of the ellipticities of the bonds between the aromatic rings of AA residues and the C- and N-terminal amides attached to them. The ellipticity of such bonds illustrates their π character. All solvents for these DFT calculations are implemented implicitly. (a) DFT-optimized structure of the ground state of Box$_5$ in DCM. (b) Dependence of the average ellipticities for Box$_5$ on the solvents, Tol, DCM, and MeCN. (c) DFT-optimized structure of the ground-state of Aaa$_z$-Box$_3$-Aaa$_z$ in DCM. (d) Dependence of the average ellipticities for Aaa$_z$-Box$_3$-Aaa$_z$ on the solvents, Tol, DCM, and MeCN. (b and d) For each residue, the ellipticities of the bonds of its aromatic ring with the C- and N-terminal amides attached to it is calculated. For each of the two pentamers, the obtained values for the ellipticities of the C-terminal amide-ring bonds of the five resides are averaged and designated as “C-ter.” In the same manner, the values for the ellipticities of the N-terminal amide-ring bonds of the five residues are averaged and designated as “N-ter.”

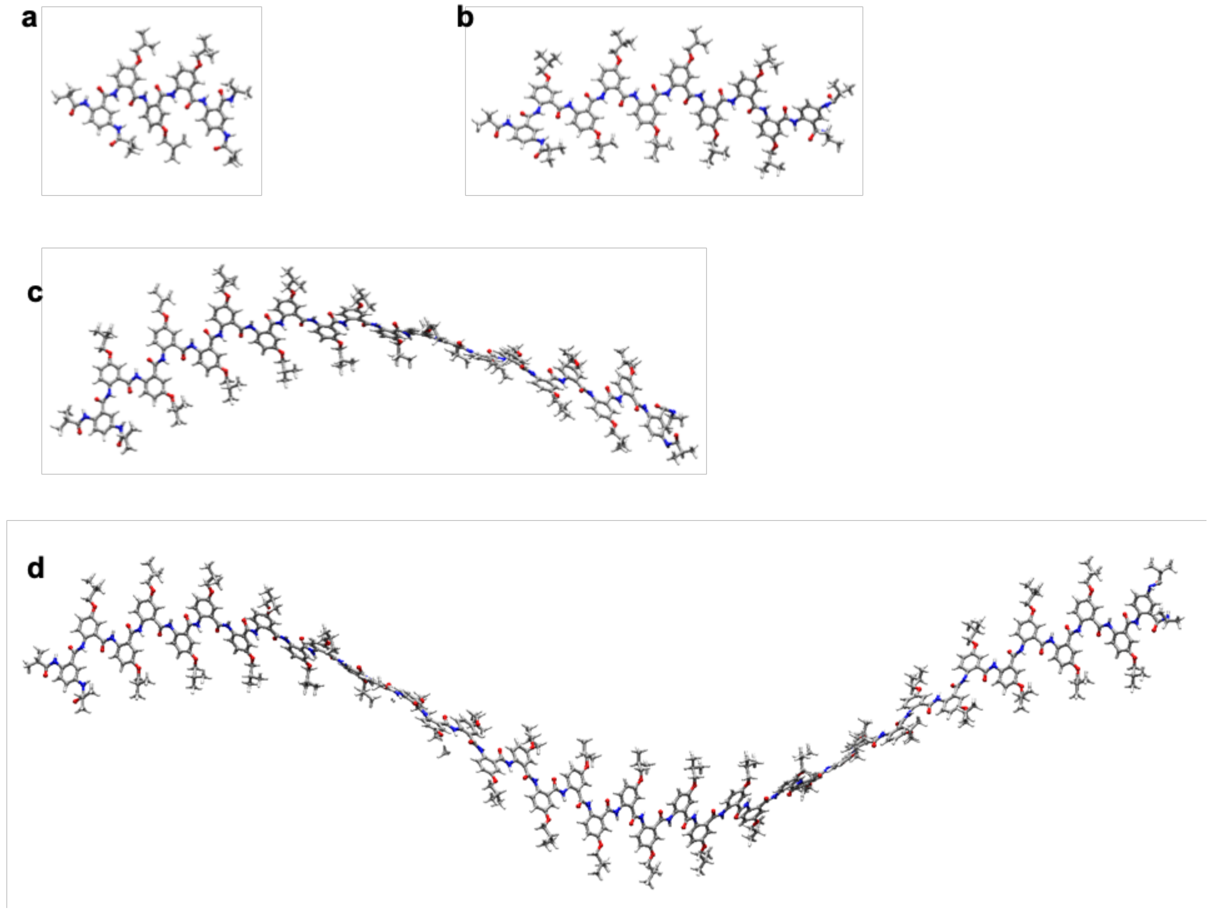

Figure S4. Initial structures of Aaa-capped Box oligomers with a different number of residues, (a) *l*=5, (b) *l*=10, (c) *l*=20, and (d) *l*=40, respectively. Gray, white, red, and blue represent carbon, hydrogen, oxygen, and nitrogen, respectively.

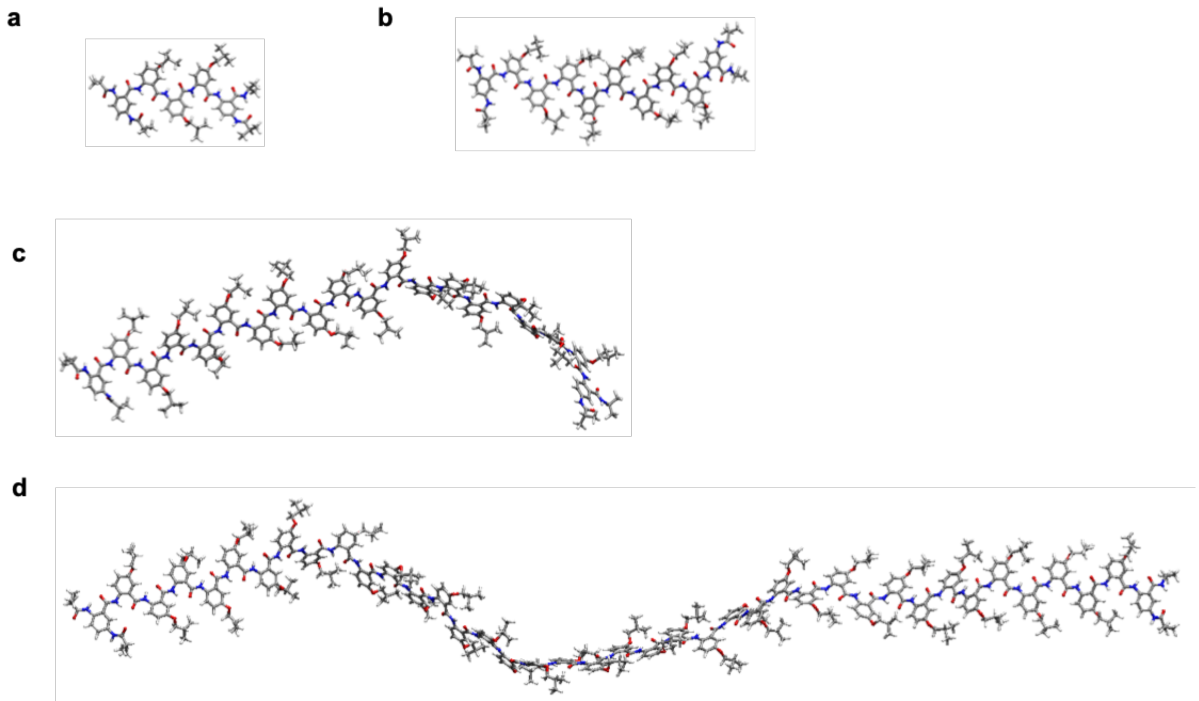

Figure S5. Snapshot structures after 1 ns MD simulations for Aaa-capped Box oligomers with a different number of residues, (a) *l*=5, (b) *l*=10, (c) *l*=20, and (d) *l*=40, respectively. Gray, white, red, and blue represent carbon, hydrogen, oxygen, and nitrogen, respectively.

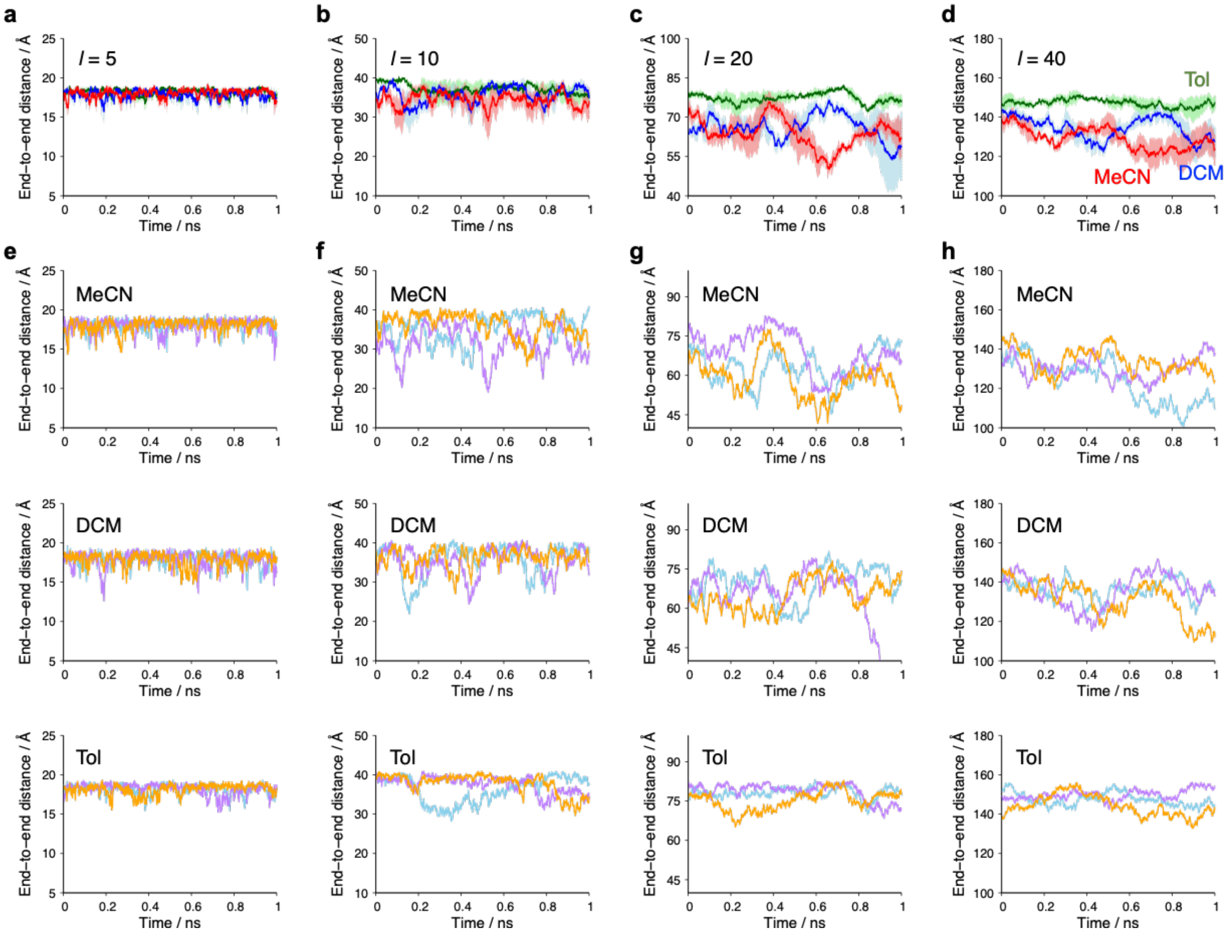

Figure S6. Average end-to-end distances for the AA oligomers, (a) *l*=5, (b) *l*=10, (c) *l*=20, and (d) *l*=40, respectively, in three solvents over time, where shaded areas represent standard error from three replicas. End-to-end distances of each replica for the AA oligomers, (e) *l*=5, (f) *l*=10, (g) *l*=20, and (h) *l*=40, respectively, in three solvents over time (first row: MeCN, second row: DCM, and third row: Tol).

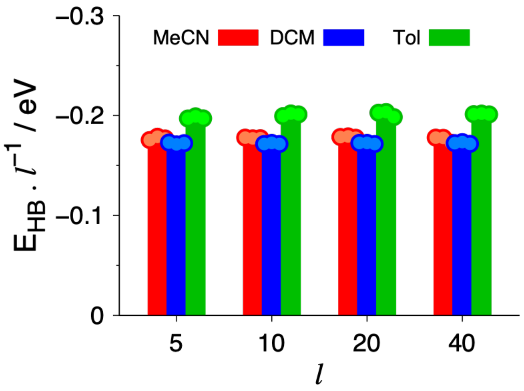

Figure S7. Average hydrogen-bond energy per residue during the 1 ns MD simulations.

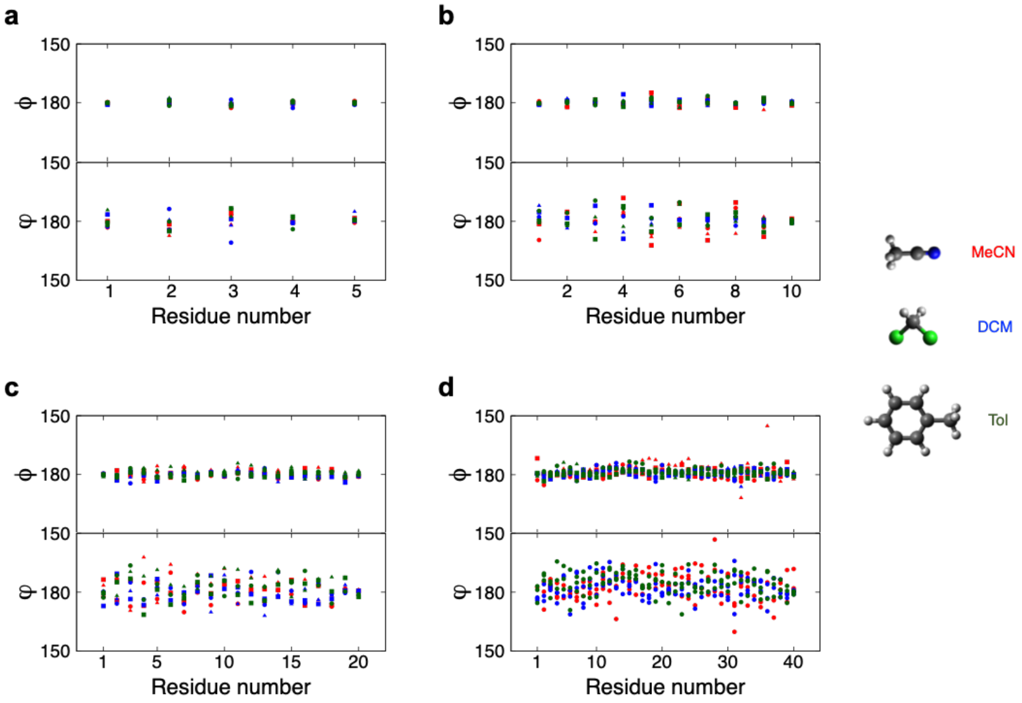

Figure S8. Average dihedral angles, ϕ and φ, during the 1 ns MD simulations for Aaa-capped Box oligomers with various numbers of residues, (a) l=5, (b) l=10, (c) l=20, and (d) l=40, respectively. Red, blue, and green points represent MeCN, DCM, and Tol solvents, respectively, and different point shapes (circle, square, and triangle) indicate three replicas.

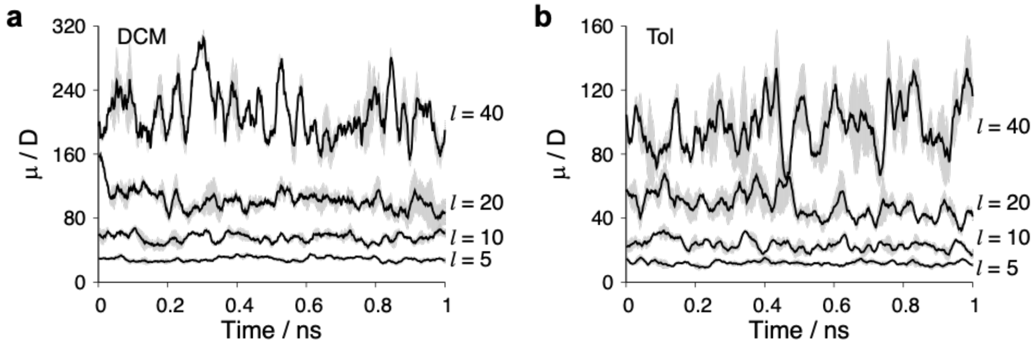

Figure S9. Average dipoles of three replicas from moving averages with a window size of 20 ps for the AA oligomers in (a) DCM and (b) Tol, respectively, where shaded areas represent standard error from three replicas.

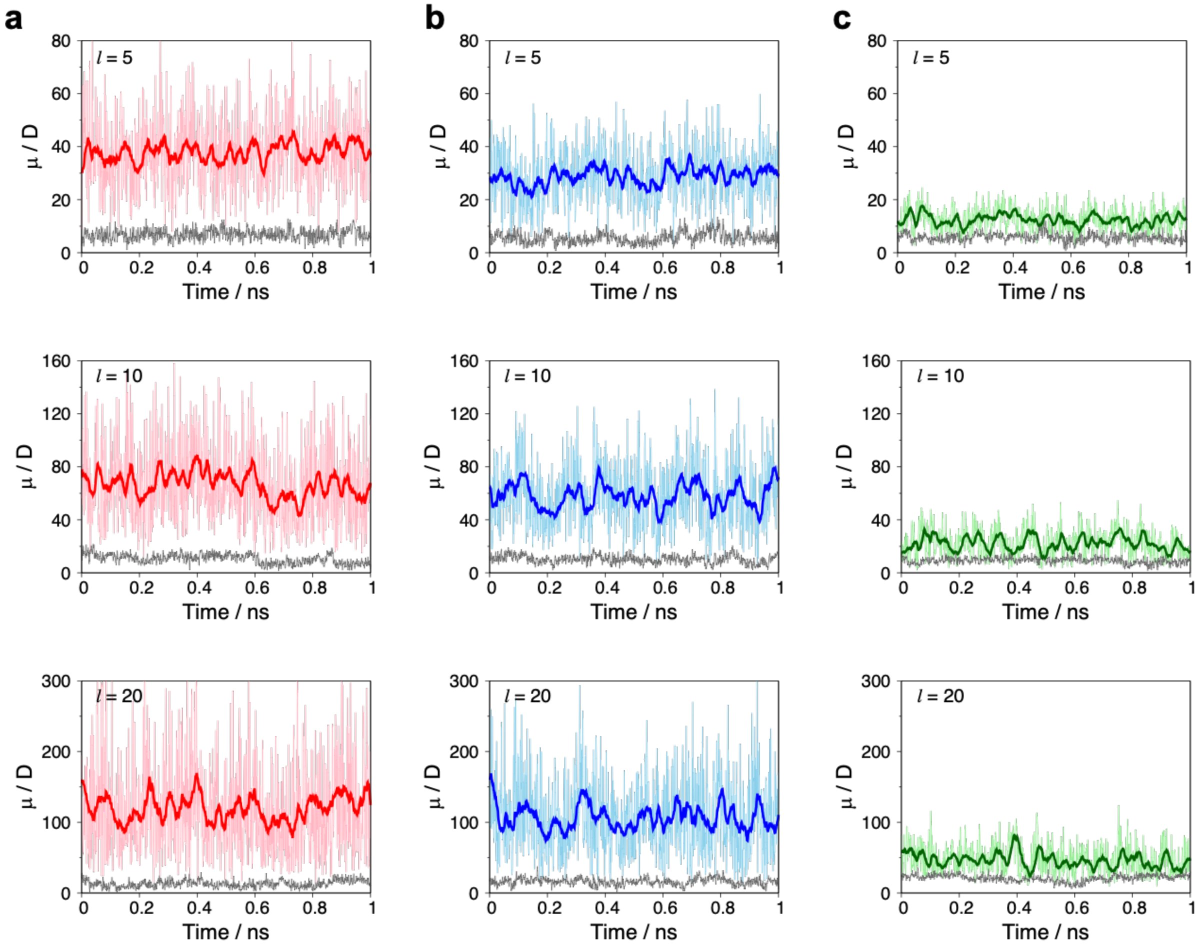

Figure S10. Dipole fluctuations of the AA oligomers in (a) MeCN, (b) DCM, and (c) Tol solvents over time, where the thin pink, sky-blue, and light-green lines show the dipole of the AA oligomer at each picosecond (ps), and the thick red, blue, and green lines indicate moving averages with a window size of 20 ps. The gray lines show the dipoles of the AA oligomers in the gas phase calculated by removing solvents from the trajectories.

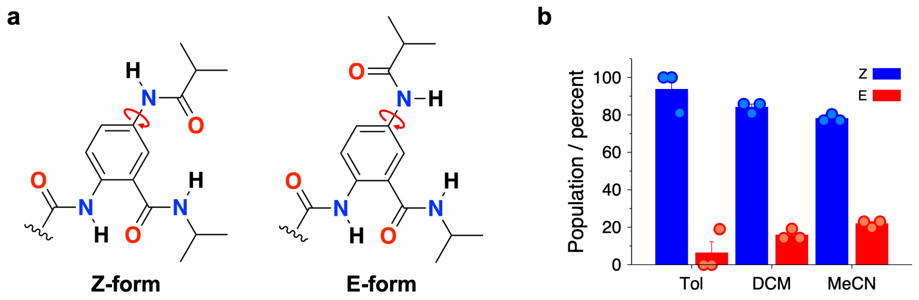

Figure S11. (a) *Z*- and *E*-conformations of terminal AA residue. For the amide bonds at the side chains of the terminal Aaa residue, the planar conformation is favorable due to electronic stabilization from π-conjugation with the aromatic ring. Nevertheless, the side chain amides can point either orthogonal to the backbone axes (*E*-conformation) or rotate to be parallel to the backbone (*Z*-conformation). (b) The population of *Z*-conformation for C-terminus of five oligomers during the 1 ns MD simulations. The result shows that the increased solvent polarity enhances the population of the *E*-conformation (6% in Tol, 16% in DCM, and 22% in MeCN, respectively), which is in good agreement with the DFT calculations (Figure S12). The DFT calculations in vacuum using the monomer Aaa residue and Aaa-Box show that the *Z*-conformation is energetically more stable by ~0.2 eV than the *E*-conformation, while the *E*-conformation exhibits a larger dipole moment than the *Z*-conformation.[8]

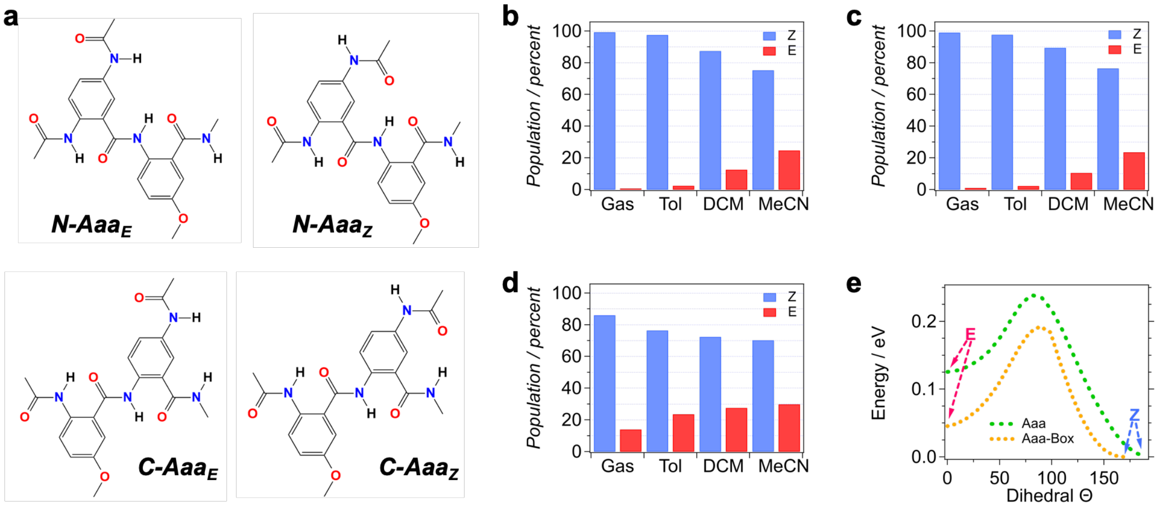

Figure S12. Population of *E* vs. *Z* for Aaa-Box and Box-Aaa from DFT. (a) The chemical structures of *E*- (left) and *Z*- (right) conformations of N-terminus (upper) and C-terminus (bottom) Aaa-Box dimer, respectively. (b) Population of relative equilibrium ratios of Aaa_Z and Aaa_E. (c) Population of relative equilibrium ratios of C-Aaa_Z vs. C-Aaa_E showing that addition of Box at the N-terminus has no effect on the Aaa-*Z* vs -*E* relative populations. (d) Population of relative equilibrium ratios of N-Aaa_Z vs. N-Aaa_E showing that addition of Box at the C-terminus has a substantial effect on the Aaa-*Z* vs -*E* relative populations. (e) The energy barrier of the *E* to *Z* flip of Aaa amide as part of the N-Aaa_X and Aaa alone in gas phase.

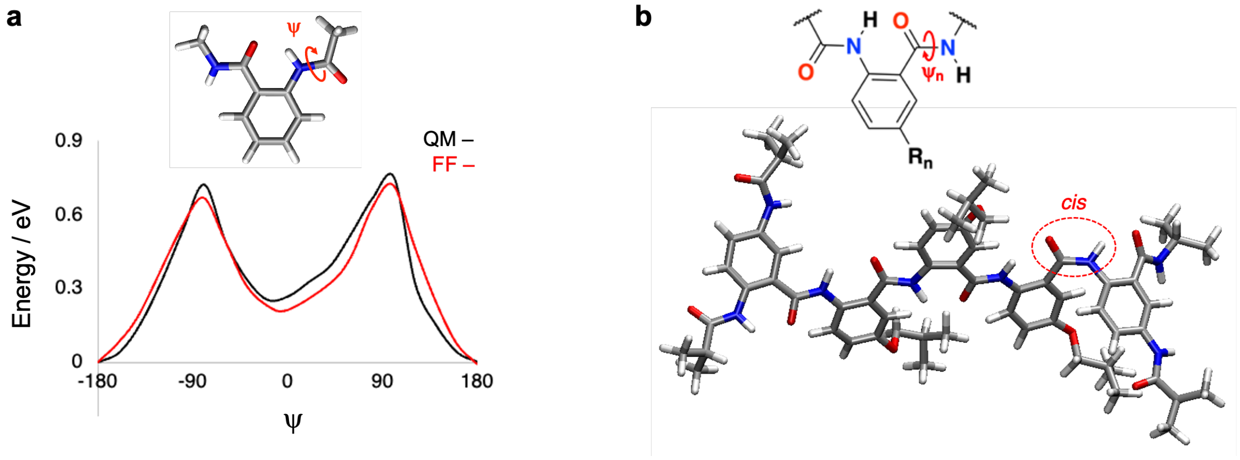

Figure S13. (a) The energy profiles along peptide dihedral angle by QM (black) and UFF/PQEq FF (red), and (b) a snapshot structure of the Aaa-capped Box oligomer structure solvated by MeOH in which a peptide bond is in a *cis*-conformation (red circle). The solvent molecules are omitted for clarity.

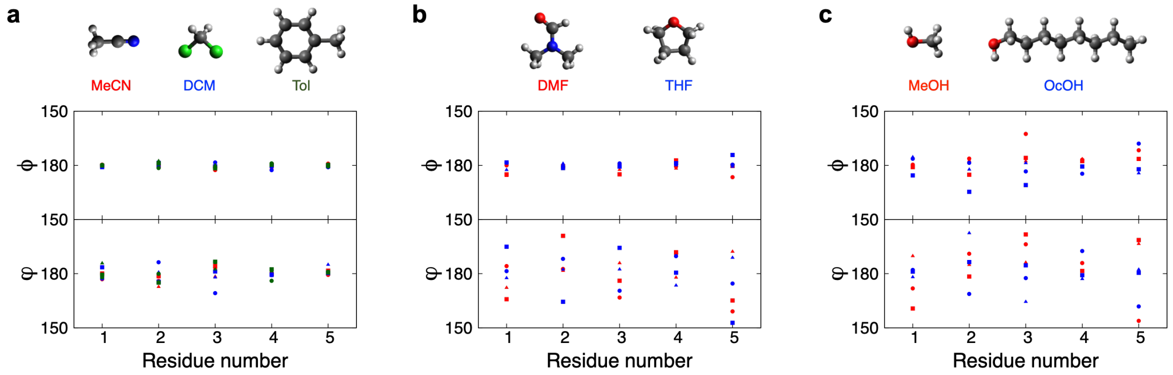

Figure S14. Average dihedral angles, ϕ and φ, during the 1 ns MD simulations for Aaa-capped Box oligomers in different types of solvents in terms of the HB capability, (a) no HB (MeCN, DCM, and Tol), (b) HB acceptor (DMF and THF), and (c) HB donor and acceptor (MeOH and OcOH), respectively, where different point shapes (circle, square, and triangle) indicate three replicas.

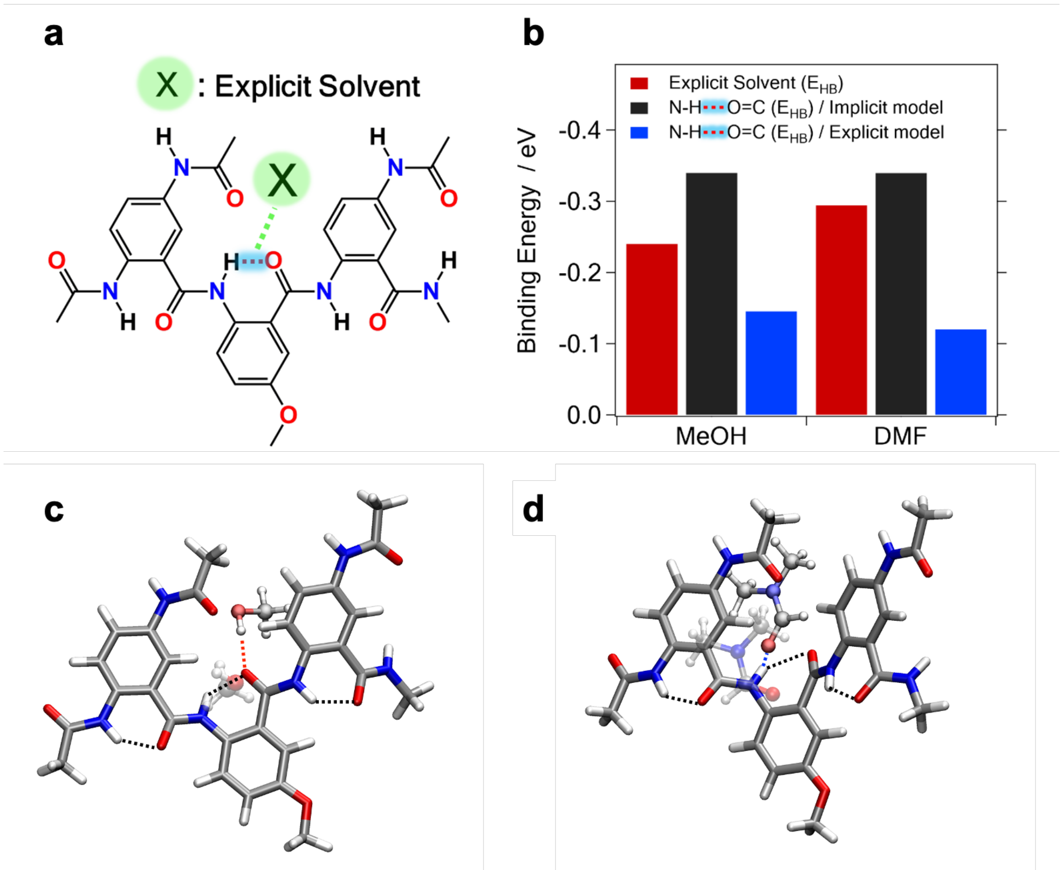

Figure S15. DFT analysis of HB between an AA trimer and solvents capable of acting as HB donors and acceptors. The structure of Aaa-Box-Aaa by itself and of Aaa-Box-Aaa with explicitly introduced molecules of MeOH or DMF are optimized in the corresponding implicitly introduced solvent media at the B3LYP/6-31G(d) level of theory. (a) Chemical structure of Aaa-Box-Aaa and the interaction of its backbone amides with an explicitly introduced solvent molecule, X. (b) HB binding energy (E_{HB}) calculated from the electron density, $\rho(r)$, at the bond critical point (BCP) of each HB using parameters from the regression analysis of neutral complexes.[9] Red bars: E_{HB} of the intermolecular HB between the explicitly introduced solvent molecules and the AA trimer in the implicit solvent dielectric media. Black bars: E_{HB} of the middle intramolecular HB (at the Box residue, highlighted in blue, Figure S15a) of the AA trimer in an implicit solvent with no explicit solvent molecules. Blue bars: E_{HB} of the middle intramolecular HB (at the Box residue, highlighted in blue, Figure S15a) of the AA trimer in an implicit solvent medium plus an explicit solvent molecule bound to it. (c,d) The DFT-optimized structures with explicit solvent molecules from this analysis show (c) MeOH acting as an HB donor and binding to the amide carbonyl; and (d) DMF acting as and HB acceptor and binding to the amide proton. In each system, two explicit solvent molecules are included, where the one that does not form an HB is depicted with transparency. The DFT values of the intramolecular HB energies in the absence of explicit solvents (black bars) are more negative than those obtained from the MD structures for a non-HB solvent, MeCN, with similar polarity (Figure 5c). This difference is consistent with the fact that the DFT analysis estimates E_{HB} from only optimized structures, while the E_{HB} from the MD analysis is an average from all geometries in the trajectory. Concurrently, the reduced DFT values of the intramolecular HB energies in the presence of explicit HB solvents (blue bars), while similar to those obtained from the MD simulations in MeOH and DMF, are not quite as negative (Figure 5c). Again, the DFT estimates of E_{HB} are for optimized structures and they may overestimate the destabilization of the AA intramolecular HBs by intermolecular HB with solvents.

Table S9. Dihedral angles of the backbone for Aaa-Box_3-Aaa and Box_5 from DFT optimizations in different implicit solvent environments.

	Residue 1[a]		Residue 2[a,b]		Residue 3[a]		Residue 4[a,b]		Residue 5[a]	
	ϕ	φ	ϕ	φ	ϕ	φ	ϕ	φ	ϕ	φ
					**Aaa-Box_3-Aaa**					
Gas	173.0	155.4	-165.1	-152.0	151.7	155.5	-157.1	-154.4	167.2	157.1
Tol	171.1	153.7	-162.5	-151.3	148.5	154.3	-154.5	-153.5	162.0	154.8
DCM	169.6	152.3	-160.9	-150.8	146.8	153.1	-153.4	-152.9	158.6	152.9
MeCN	168.8	151.8	-160.0	-150.6	146.4	152.6	-152.8	-152.5	157.5	152.3
					**Box_5**					
Gas	172.0	157.5	-163.5	-155.4	163.3	156.7	-158.2	-154.3	163.0	153.5
Tol	170.0	155.6	-160.0	-154.1	160.5	155.5	-154.8	-153.3	160.0	152.5
DCM	167.6	154.3	-157.3	-153.1	158.1	154.2	-153.6	-152.5	156.3	151.2
MeCN	166.8	153.7	-156.4	-152.7	157.0	153.5	-153.3	-152.3	154.8	150.6

[a] For residue assignments, see Figures S3a and S3c; and for designations of the dihedral angles ϕ and φ, see Figure 2a.
[b] Negative sign indicates the twisted amide bonds laying below the plane of the AA backbone, whilst no sign indicates the twisted amide bonds laying above the AA backbone.

Table S10. Comparison between Aaa-Box$_3$-Aaa dipoles obtained from different computational methods using the same DFT geometries optimized for various solvents.[a]

Method [b]	Solvent implementation	μ / D [c]				
		MeCN	DCM	Tol	Gas	Gas [d]
UFF/QEq	— [e]	1.66	1.61	1.60	1.60	1.66 (MeCN)<br>1.61 (DCM)<br>1.60 (Tol)
HF/STO-3G	Implicit	8.74	8.62	8.25	7.49	7.96 (MeCN)<br>7.92 (DCM)<br>7.83 (Tol)
AM1	Implicit	10.74	10.51	9.82	8.64	9.02 (MeCN)<br>8.99 (DCM)<br>8.92 (Tol)
DFT/6-31G(d)	Implicit	14.85	14.61	13.83	12.51	12.89 (MeCN)<br>12.85 (DCM)<br>12.75 (Tol)
UFF/PQEq	— [e]	1.59	1.51	1.48	1.62	1.59 (MeCN)<br>1.51 (DCM)<br>1.48 (Tol)
**UFF/PQEq [f]**	**Explicit**	**35.59 [g]**	**29.35 [g]**	**11.97 [g]**		**6.30 (MeCN) [h]**<br>**5.57 (DCM) [h]**<br>**5.74 (Tol) [h]**

[a] The geometry of Aaa-Box$_3$-Aaa is optimized using DFT at the B3LYP/6-31G(d) level including the Grimme dispersion DFT-D3 correction for the gas phase and for implicitly implemented MeCN, DCM and Tol. These four DFT-optimized structures of Aaa-Box$_3$-Aaa are used for single-point calculations of the permanent electric dipoles of the pentamer employing five different methods.

[b] Methods used for calculating the dipoles, μ, from the DFT-optimized structures of Aaa-Box$_3$-Aaa: UFF/QEq – universal force field (UFF) with the charge-equilibrium model (QEq); HF/STO-3G – *ab initio* Hartree-Fock (HF) with Slater type orbitals (STO) approximated using three Gaussian (3G) functions; AM1 – a semi-empirical method employing Austin model 1 (AM1); DFT/6-31G(d) – the DFT method used for computing the optimized structures for this comparative analysis; and UFF/PQEq – UFF with polarizable charge equilibrium (PQEq) model used for the MD simulations in this study.

[c] Magnitudes of the electric dipoles of the ground electronic state of Aaa-Box$_3$-Aaa.

[d] Single-point calculations for the gas phase of the DFT geometries optimized for each of the three implicitly implemented solvents.

[e] No solvating medium implemented. Indeed, the results for MeCN, DCM and Tol depict the dipoles of the DFT optimized structures optimized in these solvents (introduced implicitly). But these particular dipoles are calculated for the gas phase.

[f] Average dipole values of the structures from 1-ns MD simulations with explicitly implemented solvents.

[g] Average dipoles of Aaa-Box$_3$-Aaa calculated including all solvent molecules in the simulation box.

[h] Average dipoles of Aaa-Box$_3$-Aaa only, *i.e.*, calculated without including solvent molecules.

XYZ for optimized structures in DFT: Molecular structures of all the Aaa oligomers and monomers are optimized using the Gaussian 09 program package[10] within the density functional theory (DFT) framework. B3LYP functional[11,12] and the 6-31G(d,p) basis set are used for all DFT calculations. Solvation effects are studied using the integral equation formalism coupled with the polarizable continuum model (IEF-PCM).[13] The localized molecular orbitals are visualized using the Gaussian 09 program package, their π orbitals and bond ellipticities were identified.[14-16]

**Figure S2. Box Gas**	**Figure S12. Aaa$_E$-Box DCM**
C-1.46407100 -0.67784100 0.02488700	N 2.91680300 -3.26600600 -0.19769800
N-2.83713600 -0.93403800 0.14940000	H 3.75358200 -3.14517700 -0.74875100
C-0.51341800 -1.70134200 -0.10981800	C 2.29215100 -2.18042000 0.30399600
C-3.50330500 -2.12805000 0.01127500	C 3.00210700 -0.86214100 0.18285800
H-3.36998100 -0.10418400 0.40732500	O 1.16736200 -2.28009000 0.84096000
C-1.01096900 0.67095400 0.04021800	C 2.26997000 0.35262700 0.07585800
C 0.84796100 -1.41859900 -0.18840900	C 2.97427500 1.55366700 -0.07375500
H-0.86132000 -2.72326600 -0.15171800	C 4.37020400 1.57700700 -0.09717700
C-4.99838500 -2.03115700 0.28157800	C 5.09321600 0.38687000 0.04202100
O-2.97586600 -3.19622400 -0.27680900	C 4.39951500 -0.82016400 0.18171900
C 0.36352500 0.93083300 -0.01115500	N 0.86350300 0.29392100 0.15017400
C-1.98460200 1.80876400 0.10914600	H 0.53817000 -0.58056100 0.56097100
C 1.30043500 -0.09620300 -0.12442000	C-0.05593200 1.18628300 -0.30674200
H 1.54447900 -2.24261800 -0.28454700	O 0.25486300 2.29500700 -0.78342600
H-5.39102900 -1.01288500 0.21703200	C-1.48857200 0.74788800 -0.19031800
H-5.20145100 -2.41988800 1.28498600	C-1.80415300 -0.61763100 -0.19793600
H-5.51870000 -2.67202100 -0.43258800	C-4.15167100 -0.13501000 0.04293600
H 0.74326000 1.94313200 0.07450300	C-3.85931300 1.22628800 0.03333500
N-1.55631800 3.00858200 -0.37635500	C-2.54294100 1.69810600 -0.09303400
O-3.12867500 1.69474000 0.57532000	O 6.45508400 0.29518000 0.06023800
C-2.44447000 4.15925700 -0.40469600	N-2.23489800 3.06896100 -0.13795300
H-0.73036000 3.02557300 -0.95339000	H-1.28121000 3.24993700 -0.44809800
H-1.85070600 5.06708800 -0.53095200	C-3.01314000 4.14378200 0.20017200
H-3.17823600 4.09257900 -1.21741900	O-4.17163500 4.05960600 0.61941400
H-2.99132800 4.21110400 0.53779600	H 2.42309600 2.47786300 -0.16891100
O 2.60939800 0.29291300 -0.16219900	H 4.87185700 2.53041800 -0.20729400
C 3.62106700 -0.71158900 -0.21919200	H 4.98348300 -1.72393500 0.31850200
H 3.51426200 -1.30039700 -1.14221900	H-1.01164600 -1.35066100 -0.31552300
H 3.52007000 -1.39926500 0.63505300	H-5.17604100 -0.46560100 0.13277200
C 4.98165600 -0.01939600 -0.17864500	H-4.66288000 1.94213000 0.12487600
H 5.00897500 0.67979400 -1.02547700	C 7.21939100 1.49902500 -0.06057600
C 5.15841600 0.77830200 1.11905300	H 8.26402700 1.19126100 -0.01687000
H 4.35136500 1.50414800 1.24422800	H 7.02415000 1.99687900 -1.01753800
H 5.14954300 0.10827900 1.98765000	H 7.00517300 2.18747100 0.76516500
H 6.11252000 1.31510200 1.12071400	C-2.34283200 5.49302300 0.01803000
C 6.09685500 -1.05707800 -0.36072500	H-1.30392900 5.42866800 -0.31378400
H 5.99243400 -1.60526500 -1.30340600	H-2.91622000 6.06897800 -0.71454100
H 7.07835800 -0.57337800 -0.36078300	H-2.38452100 6.03225400 0.96848100
H 6.08899400 -1.78800500 0.45682800	C-3.11981400 -1.07757000 -0.08015300
	N-3.31899800 -2.47571800 -0.09091000

Figure S12. Aaa$_E$-Box MeCN

N 2.88235000 -3.28637800 -0.05574700
H 3.71552200 -3.20006300 -0.61875700
C 2.27561400 -2.17387900 0.40273300
C 2.99392100 -0.86862300 0.20810800
O 1.15858500 -2.23526800 0.96289600
C 2.26951800 0.34403500 0.04200100
C 2.98121300 1.53191800 -0.16580500
C 4.37752100 1.54508000 -0.19134200
C 5.09269000 0.35812300 0.00347200
C 4.39120900 -0.83659200 0.20188600
N 0.86202200 0.29586200 0.11605100
H 0.53018200 -0.56038800 0.56035000
C-0.05235600 1.18133900 -0.35998100
O 0.25756600 2.27758700 -0.86580000
C-1.48637300 0.75179200 -0.22755000
C-1.81163300 -0.61020500 -0.26177000
C-4.15125900 -0.11321900 0.02503600
C-3.84724300 1.24601900 0.04304400
C-2.52934300 1.70872700 -0.09315600
O 6.45380400 0.25861900 0.02444700
N-2.20830200 3.07814800 -0.11538500
H-1.26643100 3.26006400 -0.45800400
C-2.95845600 4.14839600 0.28951800
O-4.09738400 4.05898600 0.76154400
H 2.43661800 2.45448900 -0.30446400
H 4.88512600 2.48878100 -0.34767300
H 4.96852600 -1.73762100 0.37920600
H-1.02437100 -1.34376100 -0.40784200
H-5.17713600 -0.43661600 0.12233200
H-4.64395400 1.96561900 0.16213500
C 7.22620800 1.45103500 -0.15503700
H 8.26877200 1.13877900 -0.09752600
H 7.03262800 1.90319600 -1.13451000
H 7.01705900 2.17946500 0.63677500
C-2.28686300 5.49734700 0.11613800
H-1.27248400 5.43705500 -0.28459300
H-2.89987500 6.10634300 -0.55491100
H-2.25995300 5.99950300 1.08734700
C-3.13043500 -1.06186600 -0.13717500
N-3.34332600 -2.45673100 -0.18658600
C-4.51915200 -3.15821300 -0.14053000
H-2.50326400 -3.01249600 -0.27600100
O-5.62811000 -2.62402900 -0.03920800
C-4.37655500 -4.66553100 -0.22294800
H-3.34396300 -5.01171800 -0.30768700
H-4.82732300 -5.10477800 0.67152500
H-4.94363100 -5.01928600 -1.08870000
C-4.49166400 -3.18565700 -0.07302000
H-2.47116200 -3.02578900 -0.11977700
O-5.60695200 -2.65914400 -0.04512900
C-4.33320100 -4.69426100 -0.08788500
H-3.29534000 -5.03394700 -0.12467000
H-4.80889500 -5.10006100 0.80951700
H-4.86975600 -5.08938800 -0.95502900
C 2.32019700 -4.59461300 -0.12379400
H 1.41812100 -4.65670800 -0.74167500
H 3.05060300 -5.32274400 -0.47741200
H 2.04793500 -4.82645700 0.90860600

Figure S12. Aaa$_E$-Box Tol

N 2.97317800 -3.21106100 -0.46019000
H 3.80197000 -3.02350400 -1.00410100
C 2.32285700 -2.18066500 0.12901400
C 3.01678300 -0.84947000 0.13561500
O 1.19415300 -2.34857200 0.63424700
C 2.27059600 0.36173000 0.13246000
C 2.96251200 1.57859400 0.08369400
C 4.35763800 1.61821000 0.06542500
C 5.09445500 0.42916600 0.10867800
C 4.41432300 -0.79198900 0.14422700
N 0.86687300 0.28342500 0.20466600
H 0.55177500 -0.62094100 0.54865400
C-0.06295100 1.19619400 -0.19960900
O 0.25038900 2.33250800 -0.60105700
C-1.49223000 0.74230500 -0.11734400
C-1.79335300 -0.62779400 -0.08817900
C-4.15153300 -0.17243900 0.08532600
C-3.87802400 1.19074300 0.03414200
C-2.56463300 1.67895300 -0.07906600
O 6.45788500 0.35082400 0.12476800
N-2.27794600 3.05016900 -0.16117100
H-1.30283500 3.23387000 -0.39627000
C-3.10903800 4.12684500 0.02195200
O-4.30339000 4.04685900 0.31142500
H 2.39967800 2.50041700 0.06172000
H 4.84905500 2.58288000 0.03545400
H 5.00944600 -1.69643400 0.21063900
H-0.99435900 -1.35850300 -0.16767300
H-5.17264600 -0.51653600 0.16430100
H-4.69135800 1.89982700 0.08287400
C 7.20767900 1.56643700 0.11539500
H 8.25584000 1.26781200 0.14004400
H 7.01446700 2.14549900 -0.79589300
H 6.98109900 2.17895100 0.99654500
C-2.43703100 5.47673900 -0.16390600

C 2.27810000 -4.60556000 0.09184200
H 1.34901000 -4.67814000 -0.48292000
H 2.98562000 -5.35214400 -0.26913200
H 2.05047400 -4.80046600 1.14279400

Figure S12. Aaa$_E$ Gas

C-0.95767100 -0.65315600 -0.06638600
N-2.32988000 -0.93131900 -0.13911900
C 0.01660500 -1.66028900 0.04967000
C-2.98355800 -2.12085700 0.09304400
H-2.88644200 -0.12587900 -0.41973900
C-0.52555800 0.69984600 -0.11951800
C 1.37583800 -1.36361300 0.06360100
H-0.30700300 -2.68852100 0.12481600
O-2.43052900 -3.16187000 0.43360400
C 0.84874600 0.97781100 -0.12784400
C-1.51456800 1.82372200 -0.16866700
C 1.81060800 -0.03291600 -0.04482500
H 2.10249300 -2.15968800 0.14033900
H 1.17546200 2.00844000 -0.24366200
N-1.11682300 3.01913100 0.35460900
O-2.64926700 1.70043700 -0.65794900
H-0.31120300 3.03379800 0.96120400
C 4.29850500 -0.43675000 -0.00387500
N 3.17060100 0.35144300 -0.08804600
H 3.33122700 1.34129500 -0.20615100
O 4.27107400 -1.65307300 0.13825400
C-4.48871700 -2.05816400 -0.11229200
H-4.97528700 -2.41100800 0.80110000
H-4.86250000 -1.06125800 -0.36081000
H-4.75582700 -2.75191400 -0.91503600
C-2.03036600 4.15444000 0.39119300
H-2.46389800 4.30503700 -0.59940700
H-2.85128100 3.99007300 1.09886400
H-1.47186400 5.04643600 0.68199700
C 5.61578300 0.31730700 -0.10252800
H 6.16696200 -0.06616700 -0.96583100
H 5.51108700 1.40202900 -0.20047100
H 6.20667500 0.09430100 0.78996100

Figure S12. Aaa$_E$ DCM

C 0.94856700 -0.66278200 0.09592200
N 2.32458400 -0.94217500 0.18178400
C-0.02387600 -1.66992700 -0.00168700
C 2.99026000 -2.09642900 -0.13419500
H 2.87421300 -0.14566500 0.49973000
C 0.51872200 0.69058100 0.12481900
C-1.38384400 -1.37073300 -0.04132800
H-1.37076000 5.41225200 -0.39487100
H-2.94757800 6.00891200 -0.97191400
H-2.57325100 6.05950100 0.75151300
C-3.10273000 -1.10223800 0.01833900
N-3.27990900 -2.50376800 0.05918600
C-4.44540400 -3.23049500 0.05623100
H-2.42313700 -3.03861300 0.09888100
O-5.56512100 -2.72396900 0.00678700
C-4.26317600 -4.73696000 0.12477800
H-3.21945900 -5.06274500 0.12539300
H-4.74998900 -5.10191000 1.03378400
H-4.77866800 -5.18572100 -0.72863500
C 2.38583200 -4.54371400 -0.52227700
H 1.50651000 -4.56007500 -1.17558800
H 3.13461100 -5.23714100 -0.90742700
H 2.07745400 -4.86127100 0.47640600

Figure S12. Aaa$_E$-Box Gas

N-3.04557700 -3.21001200 0.41560900
H-3.88820900 -3.01807000 0.93496200
C-2.34901300 -2.17166500 -0.11560900
C-3.02565000 -0.83292900 -0.12751000
O-1.19931400 -2.35156100 -0.55968400
C-2.26569000 0.37128400 -0.10827200
C-2.94833600 1.59455600 -0.04620500
C-4.34236800 1.64627200 -0.03676300
C-5.09171600 0.46621300 -0.10235600
C-4.42349700 -0.75942300 -0.14685400
N-0.86609400 0.27978800 -0.17323200
H-0.55758300 -0.63662800 -0.48844800
C 0.07632100 1.20901200 0.17583100
O-0.23116500 2.36481100 0.51663500
C 1.49974000 0.74108200 0.10586700
C 1.78952100 -0.63264900 0.08178000
C 4.15209000 -0.20089900 -0.08998900
C 3.89225800 1.16429500 -0.04206500
C 2.58255800 1.66667400 0.06675400
O-6.45676300 0.40087900 -0.13257600
N 2.31202900 3.03953700 0.14108600
H 1.33361900 3.23694000 0.34877700
C 3.16838100 4.10525700 -0.01465300
O 4.36639500 4.01024900 -0.26477500
H-2.37455700 2.50889800 -0.00570500
H-4.82427700 2.61557000 0.00406100
H-5.02992100 -1.65466100 -0.23376200
H 0.98601100 -1.35778600 0.16488000
H 5.16948600 -0.55750200 -0.16574900
H 4.71151900 1.86697600 -0.09095500

H 0.29365600 -2.70159500 -0.04456600
O 2.44333400 -3.11478700 -0.56839700
C-0.85169700 0.97413900 0.10651900
C 1.51075400 1.81519400 0.19441000
C-1.81619100 -0.03758000 0.02381300
H-2.10896500 -2.16864100 -0.10570700
H-1.17911500 2.00649800 0.18922500
N 1.16720600 2.97678100 -0.40536000
O 2.60649400 1.69377200 0.77940700
H 0.35766700 3.00047800 -1.00775500
C-4.30246900 -0.42099800 0.02326700
N-3.17228700 0.35624100 0.01159900
H-3.32672200 1.35527300 0.00830800
O-4.28140300 -1.65398700 0.04125300
C 4.48884200 -2.04936600 0.09758500
H 4.99215300 -2.36454000 -0.82029700
H 4.85909900 -1.06489700 0.39285800
H 4.74148100 -2.77275200 0.87898900
C 2.05606400 4.13303400 -0.39455900
H 2.34676800 4.37169700 0.63117300
H 2.96458900 3.93915400 -0.97455800
H 1.52563300 4.98212500 -0.82658100
C-5.61181600 0.34474900 0.00978000
H-6.19180900 0.05260000 0.88965700
H-5.49355700 1.43099700 0.00499900
H-6.17890300 0.04407100 -0.87582600

Figure S12. Aaa$_E$ Tol

C 0.95426000 -0.65724500 0.07970100
N 2.32913100 -0.93377300 0.15405500
C-0.01857400 -1.66496300 -0.02668200
C 2.98981600 -2.10868200 -0.10715000
H 2.88195600 -0.13021800 0.44757900
C 0.52194500 0.69551700 0.12463600
C-1.37870300 -1.36835900 -0.04562400
H 0.30259400 -2.69443800 -0.09096500
O 2.44029600 -3.14566100 -0.47901000
C-0.85073300 0.97490700 0.12674100
C 1.51147500 1.82124900 0.17825600
C-1.81350700 -0.03717000 0.04770400
H-2.10372200 -2.16619900 -0.11547000
H-1.17822500 2.00618200 0.22862900
N 1.13596500 2.99826900 -0.38442800
O 2.63021400 1.70052300 0.71107000
H 0.32632800 3.01418000 -0.98628800
C-4.30106100 -0.43124700 0.00273100
N-3.17203400 0.35036100 0.07703000
H-3.33133500 1.34338500 0.17301800
C-7.19267600 1.62061700 -0.11971600
H-8.24386400 1.33338600 -0.16107100
H-7.00703400 2.19007800 0.80006300
H-6.94995300 2.24229700 -0.99102000
C 2.50751900 5.46516900 0.15195400
H 1.43385300 5.41594300 0.35266500
H 3.00367600 5.99086600 0.97300200
H 2.67900700 6.04489000 -0.75943300
C 3.09273400 -1.11940700 -0.02348400
N 3.25558000 -2.52349400 -0.06456300
C 4.41809500 -3.25987800 -0.05115100
H 2.39359500 -3.04820400 -0.11641200
O 5.53878300 -2.76636300 0.00751000
C 4.22257900 -4.76703800 -0.11951500
H 3.17631600 -5.08625300 -0.14171600
H 4.72719000 -5.13880100 -1.01583600
H 4.71643200 -5.21669900 0.74619700
C-2.46707000 -4.54494200 0.48949000
H-1.64799000 -4.58702700 1.21666500
H-3.24709000 -5.25016800 0.78208300
H-2.06995800 -4.82849600 -0.48763500

Figure S12. Aaa$_E$ MeCN

C 0.94735900 -0.66434900 0.09908700
N 2.32409600 -0.94359900 0.18559900
C-0.02480000 -1.67160200 0.00526600
C 2.99117000 -2.09316100 -0.13875500
H 2.87314800 -0.14719700 0.50513300
C 0.51766300 0.68906800 0.12406400
C-1.38494800 -1.37209600 -0.03773600
H 0.29199500 -2.70371100 -0.03205200
O 2.44425200 -3.11045400 -0.57929700
C-0.85211000 0.97330400 0.10198900
C 1.50988400 1.81398300 0.19711500
C-1.81696900 -0.03866700 0.02036600
H-2.10963700 -2.17047600 -0.10016200
H-1.17966100 2.00580400 0.17900800
N 1.17546600 2.97039700 -0.41271600
O 2.59962700 1.69245100 0.79587800
H 0.36191100 2.99881000 -1.00962200
C-4.30244000 -0.41775000 0.02656200
N-3.17226100 0.35660200 0.00056300
H-3.32492500 1.35591200 -0.02246100
O-4.28203600 -1.65175700 0.07030900
C 4.48974100 -2.04632400 0.08973100
H 4.99162100 -2.34911700 -0.83315700
H 4.85866700 -1.06461900 0.39513100
H 4.74603700 -2.77788000 0.86228200

O-4.27684600 -1.65460200 -0.11391400
C 4.49265100 -2.04709100 0.10298200
H 4.98400800 -2.36927300 -0.81919500
H 4.86085500 -1.05661800 0.38171600
H 4.76140700 -2.76203300 0.88636000
C 2.03584100 4.14552100 -0.40483000
H 2.40727400 4.34153700 0.60321100
H 2.89702100 3.96666900 -1.05810700
H 1.48372000 5.01586300 -0.76280300
C-5.61346300 0.32924400 0.07930200
H-6.16294500 -0.01877700 0.95875600
H-5.50011300 1.41519000 0.13759000
H-6.20925100 0.07839000 -0.80240100

Figure S12. Aaa$_Z$-Box MeCN

N-2.89735800 2.84670800 0.26623500
H-3.65637200 2.73991300 -0.39101800
C-2.26664800 1.75050200 0.72852500
C-2.83506600 0.41511600 0.34099400
O-1.24373600 1.84058300 1.44010800
C-1.97319400 -0.68010200 0.06268300
C-2.54027700 -1.90751800 -0.29849800
C-3.92667100 -2.06924000 -0.37306400
C-4.77545200 -0.99656700 -0.07942800
C-4.21806500 0.23863500 0.27512600
N-0.58137200 -0.47016900 0.16308100
H-0.34692300 0.36342700 0.70067600
C 0.43771700 -1.18973500 -0.37584300
O 0.27990100 -2.28452300 -0.94970500
C 1.79644700 -0.56581900 -0.22710000
C 1.91376600 0.83243700 -0.27914800
C 4.29409800 0.65354300 0.04372100
C 4.19617700 -0.73179200 0.08024500
C 2.95350800 -1.37029400 -0.07109400
O-6.13940700 -1.04826000 -0.10232400
N 2.82141400 -2.77134100 -0.08817900
H 1.93006600 -3.08532400 -0.46609500
C 3.69329900 -3.72367300 0.36521600
O 4.78793700 -3.47025800 0.88014100
H-1.89240500 -2.74271000 -0.52153100
H-4.32159100 -3.03902300 -0.64890700
H-4.89203500 1.05200100 0.52308000
H 1.04192100 1.44789200 -0.44475600
H 5.27268600 1.11397400 0.15009800
H 5.08418400 -1.33040300 0.22066400
C-6.76773600 -2.28761600 -0.44796200
H-7.83999000 -2.09822800 -0.40106900
H-6.49463300 -2.59926000 -1.46271100
C 2.05828900 4.13115600 -0.39019500
H 2.30550000 4.39640600 0.64077100
H 2.98994200 3.92744900 -0.92818300
H 1.54284400 4.96678100 -0.86390200
C-5.61135400 0.34685300 -0.00668200
H-6.20296500 0.05707500 0.86614500
H-5.49322500 1.43281600 -0.01352700
H-6.16600900 0.04373400 -0.89952100

Figure S12. Aaa$_Z$-Box DCM

N-2.84758200 2.80632800 0.09911500
H-3.56319400 2.66320100 -0.59868000
C-2.26992400 1.73420400 0.67528700
C-2.83942000 0.38495500 0.34057900
O-1.29141300 1.85329400 1.44017200
C-1.97360600 -0.72244900 0.13730000
C-2.53231400 -1.96527700 -0.17817700
C-3.91726000 -2.12861500 -0.27858900
C-4.77064800 -1.04190200 -0.05898600
C-4.22024600 0.20959100 0.24760400
N-0.58445200 -0.50495800 0.25839800
H-0.36156900 0.33896300 0.78331800
C 0.44275000 -1.20936000 -0.28662900
O 0.29556200 -2.31256200 -0.84640400
C 1.78971000 -0.55471300 -0.16477400
C 1.87058800 0.84692900 -0.19552400
C 4.26127700 0.72579500 0.06328600
C 4.20125600 -0.66215000 0.07895100
C 2.97232500 -1.33088700 -0.05600500
O-6.13405800 -1.09353400 -0.11340500
N 2.87547700 -2.73305800 -0.10158000
H 1.97245900 -3.05905700 -0.44098900
C 3.80326000 -3.67511000 0.25409600
O 4.92383200 -3.41015000 0.70052000
H-1.87964400 -2.80973300 -0.34690300
H-4.30794600 -3.11009000 -0.51667800
H-4.89666700 1.03698700 0.43579600
H 0.98049800 1.44445200 -0.32626000
H 5.22940700 1.21090100 0.15402800
H 5.10699000 -1.24028000 0.18814500
C-6.75584200 -2.34875600 -0.40471700
H-7.82879400 -2.15752900 -0.39049800
H-6.46236800 -2.71519000 -1.39542900
H-6.50712500 -3.09878200 0.35515700
C 3.35761100 -5.11198800 0.05795600
H 2.33657300 -5.21015200 -0.31790400
H 4.04443600 -5.59510600 -0.64327700
H 3.44022200 -5.63459000 1.01511500

H-6.50303300 -3.07675300 0.26515800
C 3.22194700 -5.15445800 0.19063500
H 2.22423000 -5.23991600 -0.24575700
H 3.93728500 -5.68011200 -0.44883200
H 3.22938900 -5.64398700 1.16856800
C 3.15853400 1.45613500 -0.14849000
N 3.34160900 2.85523300 -0.19390100
C 2.43753400 3.84067900 -0.49333400
H 4.28489600 3.16579300 -0.00426700
O 1.25678500 3.61810300 -0.77916300
C 2.98228200 5.25473100 -0.44829800
H 4.04570200 5.31503900 -0.20574600
H 2.81006700 5.72335300 -1.42123500
H 2.41485000 5.81891800 0.29757700
C-2.41141000 4.19014400 0.56648700
H-1.38030400 4.31079600 0.22034400
H-3.05389900 4.91064300 0.06034600
H-2.44072900 4.37513500 1.64404200

Figure S12. Aaa$_Z$ Gas

C-1.18503000 -0.66103500 -0.05739900
N-2.56677300 -0.43709700 -0.08286500
C-0.63190100 -1.95407900 0.02302800
C-3.59305600 -1.33438900 0.10141600
H-2.80200900 0.53658000 -0.28291200
C-0.30106500 0.45059700 -0.11988000
C 0.74324600 -2.13910900 0.01903100
H-1.29819700 -2.80169400 0.09332800
O-3.43911000 -2.52975400 0.33907200
C 1.08538900 0.23505600 -0.15761800
C-0.82557600 1.85707700 -0.14259000
C 1.62226700 -1.05043700 -0.07925600
H 1.13580000 -3.15146400 0.08525900
H 1.76994000 1.06183300 -0.26983900
N 0.02505200 2.84707900 0.23211000
O-1.99175800 2.13409900 -0.48086300
H 0.92035300 2.61338200 0.63262900
C 4.05316800 -0.42176100 0.02473100
N 3.01239900 -1.31081800 -0.11084700
H 3.26350700 -2.28572700 -0.19085500
O 3.89988700 0.78890700 0.16551000
C-4.98214300 -0.72817800 -0.01458200
H-5.52591400 -0.93420100 0.91165000
H-4.98164400 0.34870900 -0.20229700
H-5.51279900 -1.23525000 -0.82577400
C-0.40559300 4.23830800 0.24185200
H-0.82625100 4.50651400 -0.73047300
H-1.17532300 4.41084400 1.00221900
C 3.10133000 1.50054100 -0.08845300
N 3.24230500 2.90593700 -0.11165000
C 2.31981700 3.86252300 -0.45068600
H 4.16819500 3.24386700 0.11359700
O 1.16428800 3.60245000 -0.79646400
C 2.81210300 5.29460700 -0.36923500
H 3.85958200 5.39063000 -0.07384300
H 2.67054400 5.76564600 -1.34587100
H 2.18916500 5.83165100 0.35193200
C-2.32840000 4.15500600 0.31277700
H-1.27788600 4.21130800 0.01049400
H-2.92103400 4.85066800 -0.28186600
H-2.40398700 4.42884600 1.36891400

Figure S12. Aaa$_Z$-Box Tol

N 2.88235000 -3.28637800 -0.05574700
H 3.71552200 -3.20006300 -0.61875700
C 2.27561400 -2.17387900 0.40273300
C 2.99392100 -0.86862300 0.20810800
O 1.15858500 -2.23526800 0.96289600
C 2.26951800 0.34403500 0.04200100
C 2.98121300 1.53191800 -0.16580500
C 4.37752100 1.54508000 -0.19134200
C 5.09269000 0.35812300 0.00347200
C 4.39120900 -0.83659200 0.20188600
N 0.86202200 0.29586200 0.11605100
H 0.53018200 -0.56038800 0.56035000
C-0.05235600 1.18133900 -0.35998100
O 0.25756600 2.27758700 -0.86580000
C-1.48637300 0.75179200 -0.22755000
C-1.81163300 -0.61020500 -0.26177000
C-4.15125900 -0.11321900 0.02503600
C-3.84724300 1.24601900 0.04304400
C-2.52934300 1.70872700 -0.09315600
O 6.45380400 0.25861900 0.02444700
N-2.20830200 3.07814800 -0.11538500
H-1.26643100 3.26006400 -0.45800400
C-2.95845600 4.14839600 0.28951800
O-4.09738400 4.05898600 0.76154400
H 2.43661800 2.45448900 -0.30446400
H 4.88512600 2.48878100 -0.34767300
H 4.96852600 -1.73762100 0.37920600
H-1.02437100 -1.34376100 -0.40784200
H-5.17713600 -0.43661600 0.12233200
H-4.64395400 1.96561900 0.16213500
C 7.22620800 1.45103500 -0.15503700
H 8.26877200 1.13877900 -0.09752600
H 7.03262800 1.90319600 -1.13451000

H 0.45978900 4.86993100 0.45009400
C 5.44079000 -1.03977500 -0.01592700
H 5.96120400 -0.77698400 0.90919200
H 5.44687500 -2.12730000 -0.13348100
H 5.99479600 -0.58899000 -0.84411100

Figure S12. Aaa$_Z$-Box Gas

N-2.92997900 2.72627900 0.05829400
H-3.61379600 2.54140400 -0.65995300
C-2.30348900 1.67713600 0.64471400
C-2.84327900 0.30712600 0.34611600
O-1.31643400 1.84839400 1.37790100
C-1.95120400 -0.78786800 0.18724700
C-2.47784500 -2.04669900 -0.12223100
C-3.85655000 -2.23669600 -0.25222500
C-4.73633800 -1.16516500 -0.06840900
C-4.21825600 0.10105100 0.22729100
N-0.57415000 -0.53684300 0.33144400
H-0.38495000 0.33628700 0.82177600
C 0.48300400 -1.24422600 -0.15881200
O 0.37400400 -2.39185000 -0.62525800
C 1.80083800 -0.52558900 -0.09275300
C 1.81379900 0.87816900 -0.13495400
C 4.21248600 0.87429600 0.05750600
C 4.22431000 -0.51431100 0.08201100
C 3.02505200 -1.24280100 -0.02075200
O-6.09937400 -1.24375100 -0.15066000
N 2.99508100 -2.64372400 -0.06297600
H 2.08124400 -3.01077600 -0.32667500
C 4.01638200 -3.53725700 0.15772700
O 5.16495600 -3.22138800 0.45772800
H-1.80064100 -2.87669800 -0.26576900
H-4.22254800 -3.22917500 -0.48547000
H-4.91783900 0.91442900 0.39061500
H 0.89582700 1.43660400 -0.24391600
H 5.15919300 1.40655200 0.12010500
H 5.15692100 -1.05248300 0.17140800
C-6.68733100 -2.51005000 -0.42874900
H-7.76486100 -2.34170200 -0.43950500
H-6.36910400 -2.89316000 -1.40707500
H-6.44103400 -3.24449300 0.34901700
C 3.61725600 -4.99618600 0.00322000
H 2.56869300 -5.14287300 -0.26916900
H 4.25574400 -5.44921100 -0.76059600
H 3.81999300 -5.51018200 0.94721300
C 3.01264100 1.58896900 -0.06734300
N 3.07546100 3.00291000 -0.10048300
C 2.09691500 3.90323600 -0.44970500
H 7.01705900 2.17946500 0.63677500
C-2.28686300 5.49734700 0.11613800
H-1.27248400 5.43705500 -0.28459300
H-2.89987500 6.10634300 -0.55491100
H-2.25995300 5.99950300 1.08734700
C-3.13043500 -1.06186600 -0.13717500
N-3.34332600 -2.45673100 -0.18658600
C-4.51915200 -3.15821300 -0.14053000
H-2.50326400 -3.01249600 -0.27600100
O-5.62811000 -2.62402900 -0.03920800
C-4.37655500 -4.66553100 -0.22294800
H-3.34396300 -5.01171800 -0.30768700
H-4.82732300 -5.10477800 0.67152500
H-4.94363100 -5.01928600 -1.08870000
C 2.27810000 -4.60556000 0.09184200
H 1.34901000 -4.67814000 -0.48292000
H 2.98562000 -5.35214400 -0.26913200
H 2.05047400 -4.80046600 1.14279400

Figure S12. Aaa$_Z$ DCM

C-1.16774500 -0.67856900 -0.09035500
N-2.55722400 -0.47464200 -0.14607400
C-0.59612300 -1.96111700 -0.01253800
C-3.57210800 -1.35009300 0.13236400
H-2.80539900 0.47625500 -0.41901100
C-0.30239200 0.44577400 -0.12893400
C 0.78239500 -2.12634600 0.00455300
H-1.24396300 -2.82394900 0.03419000
O-3.40153500 -2.51709100 0.49981200
C 1.08866900 0.25807700 -0.13528700
C-0.85271700 1.84261300 -0.17791700
C 1.64596300 -1.02208400 -0.06385800
H 1.19023600 -3.13227900 0.05877100
H 1.75386600 1.10419800 -0.22076000
N-0.09258600 2.82560300 0.35086800
O-1.96721500 2.09941200 -0.68177800
H 0.74252900 2.58742600 0.86477800
C 4.07510300 -0.38228000 0.00887900
N 3.03584300 -1.27188200 -0.07498100
H 3.29233200 -2.24875300 -0.11920500
O 3.91886000 0.83945300 0.09407600
C-4.96734300 -0.78216600 -0.04850300
H-5.52108200 -0.91800000 0.88463500
H-4.98102500 0.27467300 -0.32485500
H-5.47898200 -1.36022900 -0.82394400
C-0.52761000 4.21704900 0.33959600
H-0.76020000 4.53173300 -0.68094500
H-1.42275500 4.35476700 0.95499400

H 3.97647400 3.39398400 0.13395000
O 0.96561000 3.58229000 -0.80362400
C 2.50479600 5.36483500 -0.35418300
H 3.56590900 5.52417800 -0.14104100
H 2.24974000 5.85583800 -1.29664500
H 1.91257400 5.83608700 0.43627600
C-2.39880700 4.08018300 0.20424500
H-1.36901100 4.13173700 -0.16397000
H-3.03421700 4.76426600 -0.36135100
H-2.40558300 4.37054400 1.25836000

Figure S12. Aaa$_{Z}$ Tol

C-1.17717300 -0.66872600 -0.06460800
N-2.56274500 -0.45359800 -0.10463500
C-0.61525800 -1.95650700 0.01824100
C-3.58534700 -1.34249000 0.11053600
H-2.80276100 0.51105800 -0.33728000
C-0.30180400 0.44900100 -0.11855700
C 0.76153300 -2.13256100 0.02215000
H-1.27282100 -2.81104900 0.08161000
O-3.42664700 -2.52999000 0.39861500
C 1.08697200 0.24603900 -0.14298900
C-0.83813100 1.85139900 -0.15566100
C 1.63348700 -1.03701600 -0.06698000
H 1.16101000 -3.14180900 0.08474600
H 1.76265600 1.08154100 -0.24594100
N-0.02667400 2.83990700 0.28820000
O-1.98478700 2.11765700 -0.56980700
H 0.84688000 2.60752900 0.73551700
C 4.06462600 -0.40551800 0.01555000
N 3.02316900 -1.29345700 -0.09420800
H 3.27604100 -2.26936900 -0.16283300
O 3.91109400 0.81040600 0.13817900
C-4.97584800 -0.75035600 -0.03731300
H-5.53015200 -0.93489100 0.88710800
H-4.97951000 0.32091700 -0.25293100
H-5.49340400 -1.27989600 -0.84276000
C-0.45814700 4.23182200 0.28004100
H-0.76961700 4.52428900 -0.72601200
H-1.30470400 4.38612000 0.95738100
H 0.37851200 4.85620800 0.59613100
C 5.44971700 -1.02428000 -0.02990300
H 5.98823200 -0.73143200 0.87553200
H 5.45051700 -2.11456700 -0.11029400
H 5.98783100 -0.60404200 -0.88442300

Figure S12. Box-Aaa$_{E}$ Gas

C 1.00649600 -0.72319900 -0.26006900

H 0.27992200 4.83509200 0.73283200
C 5.45987000 -0.99953000 -0.01161100
H 5.98691000 -0.69601100 0.89731100
H 5.46059200 -2.09013200 -0.07761400
H 6.00813800 -0.59051000 -0.86508600

Figure S12. Aaa$_{Z}$ MeCN

C-1.16342300 -0.68275300 -0.09792100
N-2.55425100 -0.48341300 -0.16147900
C-0.58775400 -1.96306000 -0.01996300
C-3.56642600 -1.35371500 0.13699000
H-2.80548700 0.46193500 -0.44896000
C-0.30224000 0.44422700 -0.13029900
C 0.79143200 -2.12358400 0.00332100
H-1.23157800 -2.82921800 0.02066500
O-3.39154100 -2.51229700 0.53173500
C 1.08959500 0.26299400 -0.12842400
C-0.85833500 1.83860300 -0.18508400
C 1.65140600 -1.01582800 -0.05799700
H 1.20279600 -3.12800500 0.05615700
H 1.75038400 1.11339100 -0.20651200
N-0.12195200 2.81917800 0.37759000
O-1.95700900 2.09181500 -0.72578500
H 0.69551100 2.57858600 0.91848200
C 4.07975200 -0.37166900 0.00126000
N 3.04140200 -1.26260500 -0.06171200
H 3.29947600 -2.23987700 -0.08824800
O 3.92267200 0.85270900 0.05799400
C-4.96334600 -0.79565300 -0.05699900
H-5.51813700 -0.91475500 0.87778200
H-4.98076700 0.25536500 -0.35412000
H-5.47139200 -1.39041800 -0.82215500
C-0.56091200 4.20963700 0.36595500
H-0.75520500 4.53609000 -0.65892900
H-1.47868300 4.33728900 0.94926200
H 0.22902100 4.82501500 0.79685100
C 5.46451500 -0.98803900 -0.00231000
H 5.98228300 -0.67965600 0.91048000
H 5.46583300 -2.07877400 -0.06157400
H 6.02098600 -0.58386700 -0.85269500

Figure S12. Box-Aaa$_{E}$ DCM

C 1.00522100 -0.72665100 -0.37906700
N 0.01891800 0.09812900 0.07142000
H 0.28312600 0.98591400 0.49932800
C-1.37637400 -0.07632300 0.02024100
C-2.20635200 1.06102400 0.20945400
C-3.59550900 0.89722200 0.22719500

N 0.00317100 0.13448000 0.10565700
H 0.25471900 1.05025700 0.47411300
C-1.38545300 -0.05748300 0.04582300
C-2.23135500 1.07951800 0.15581100
C-3.62039100 0.89870400 0.17955400
C-3.36554800 -1.47910900 -0.10310000
C-1.98297000 -1.32042200 -0.10421800
C-1.65491400 2.45953900 0.24672200
O-0.53867900 2.68687800 0.74119000
N-2.41320500 3.48048800 -0.24221900
H-3.18605100 3.25735800 -0.85072600
O 0.78123000 -1.88515800 -0.63693000
C 2.39771700 -0.16779300 -0.16300600
C 3.52635600 -1.03523000 -0.05977600
C 4.80183400 -0.46445100 0.06684000
C 4.98284500 0.91815100 0.05769700
C 3.88262500 1.77025600 -0.08168700
C 2.60513200 1.21914900 -0.18726200
N 3.33061800 -2.42570100 -0.09055100
H 2.38018700 -2.68473600 -0.34999900
C 4.23163600 -3.43269600 0.15553600
O 5.40872900 -3.25925900 0.46394700
O 3.94784500 3.13722200 -0.13078800
H-4.26009900 1.76412000 0.33492700
H-3.79703200 -2.46419000 -0.20782500
H-1.34763800 -2.18619800 -0.21738400
H 5.65410300 -1.12038600 0.17175400
H 5.98936200 1.30835200 0.14943100
H 1.78198900 1.91554400 -0.30383300
C 5.22424500 3.75394300 -0.01648200
H 5.69976500 3.51761900 0.94459900
H 5.89058900 3.45367800 -0.83604700
H 5.04222400 4.82794800 -0.07419100
C-1.90713700 4.84826000 -0.23777000
H-1.07625200 4.97256800 -0.94161000
H-2.71993000 5.52480400 -0.50901200
H-1.54381000 5.09861600 0.76078700
C-4.20531200 -0.36590900 0.05906200
N-5.61460200 -0.44439200 0.12112100
C-6.42787500 -1.55249200 0.00457500
H-6.08538600 0.43438900 0.28158000
O-6.00853400 -2.68671500 -0.18925100
C-7.91647200 -1.27152400 0.13649400
H-8.41550600 -1.63305700 -0.76667700
H-8.30185300 -1.85195000 0.97947300
H-8.16723800 -0.21711500 0.28606300
C 3.65712800 -4.83408500 0.01602500
H 3.79860300 -5.35994700 0.96457700
C-3.37118300 -1.46473500 -0.19352700
C-1.98540900 -1.32212000 -0.19143200
C-1.61082600 2.42600400 0.40241600
O-0.49524300 2.58893800 0.93989900
N-2.33016500 3.48231600 -0.03331100
H-3.15052800 3.32342100 -0.59902600
O 0.78359600 -1.85759600 -0.85012900
C 2.39912400 -0.17897200 -0.26111400
C 3.51610000 -1.04812000 -0.10286400
C 4.79281000 -0.48261900 0.01660500
C 4.98658900 0.89871100 -0.04165200
C 3.89362700 1.75196300 -0.22702300
C 2.61269800 1.20313900 -0.33379400
N 3.30350900 -2.44115100 -0.08173700
H 2.38007300 -2.70765000 -0.41713200
C 4.13753900 -3.43506400 0.34900200
O 5.27208700 -3.24449100 0.80108300
O 3.96868700 3.11343800 -0.32202200
H-4.22769600 1.75686500 0.42933900
H-3.81107400 -2.43867100 -0.34960100
H-1.36269500 -2.18979100 -0.35121400
H 5.64255300 -1.13486500 0.15609900
H 5.99417000 1.28577100 0.04675600
H 1.78774400 1.88891000 -0.49375000
C 5.25593100 3.72927600 -0.22800300
H 5.72298000 3.52528500 0.74277400
H 5.91724200 3.38991800 -1.03411900
H 5.07969600 4.80028400 -0.32845300
C-1.83778300 4.84831300 0.10687300
H-0.92421300 4.99845700 -0.47748300
H-2.60992600 5.53247100 -0.24577300
H-1.61500400 5.06223500 1.15504800
C-4.19720100 -0.35223900 0.02854200
N-5.60566100 -0.40951100 0.07100600
C-6.43964700 -1.49276500 -0.07080600
H-6.06568300 0.47735500 0.23218700
O-6.03549800 -2.63946500 -0.26954000
C-7.91564900 -1.15498800 0.03895200
H-8.49464500 -2.06816900 -0.09546100
H-8.14388200 -0.72453800 1.01982300
H-8.20761700 -0.42657900 -0.72504500
C 3.56843300 -4.83719100 0.23188300
H 3.59696800 -5.30631800 1.21942200
H 2.54545000 -4.86788600 -0.15055500
H 4.21487900 -5.42068200 -0.43039400

Figure S12. Box-Aaa$_E$ MeCN

C 1.00391600 -0.72914000 -0.40569500

H 2.59775200 -4.85528600 -0.25357600
H 4.23338700 -5.36902300 -0.74444100

Figure S12. Box-Aaa$_E$ Tol

C 1.00462600 -0.72112300 -0.29710700
N 0.00963500 0.11705200 0.11787300
H 0.26508500 1.02162000 0.51184700
C-1.38261100 -0.06971200 0.06009300
C-2.22287600 1.06901500 0.18145200
C-3.61174000 0.89551600 0.20066600
C-3.36669800 -1.48221900 -0.09754400
C-1.98264700 -1.32913200 -0.09456300
C-1.63921500 2.44639000 0.29430400
O-0.53292600 2.65668500 0.82556400
N-2.37068400 3.46971600 -0.21251700
H-3.15401700 3.25909700 -0.81259100
O 0.78032000 -1.86705900 -0.72558800
C 2.39751800 -0.16895900 -0.19192200
C 3.52292300 -1.03720000 -0.07852200
C 4.79859900 -0.46833600 0.04508100
C 4.98373300 0.91442500 0.02670100
C 3.88503800 1.76742300 -0.12010200
C 2.60597600 1.21660700 -0.22502700
N 3.32101100 -2.42947000 -0.10044700
H 2.37913400 -2.68728700 -0.39012000
C 4.19776200 -3.43680600 0.20435200
O 5.36286400 -3.26293000 0.56837900
O 3.95296100 3.13253100 -0.17775700
H-4.24824200 1.76231100 0.35739500
H-3.80002700 -2.46552400 -0.20806900
H-1.35211300 -2.19820600 -0.20955900
H 5.65090300 -1.12287200 0.15556500
H 5.99064400 1.30350000 0.11636400
H 1.78035000 1.90889700 -0.34935000
C 5.23442700 3.74961900 -0.06903300
H 5.70766200 3.51859900 0.89347500
H 5.89683800 3.44015300 -0.88721700
H 5.05369500 4.82290800 -0.13509500
C-1.87536400 4.84130800 -0.17397700
H-0.99791200 4.96616900 -0.81770300
H-2.67049800 5.50874100 -0.50966400
H-1.58906000 5.10235100 0.84702100
C-4.20253400 -0.36656400 0.06594300
N-5.61127600 -0.43724800 0.11354500
C-6.43539200 -1.53311200 -0.02235800
H-6.07978400 0.44566800 0.26764600
O-6.02241400 -2.67344000 -0.21458400
C-7.91605300 -1.20640600 0.08474000
N 0.02155500 0.09041700 0.06006200
H 0.29003100 0.97185600 0.49877700
C-1.37476900 -0.07706700 0.00906200
C-2.19817300 1.06246300 0.21361900
C-3.58759000 0.90638300 0.22993300
C-3.37644200 -1.45247900 -0.21796600
C-1.98954300 -1.31726100 -0.21595100
C-1.59482100 2.42127700 0.42826900
O-0.48161700 2.56692900 0.97881800
N-2.30130500 3.48650100 0.00003100
H-3.12526800 3.34171400 -0.56466100
O 0.78133400 -1.85246800 -0.89477300
C 2.39861000 -0.18481000 -0.28196100
C 3.51187800 -1.05586600 -0.11515400
C 4.78985800 -0.49360900 0.00349600
C 4.98805100 0.88709300 -0.05948700
C 3.89745300 1.74250400 -0.25010100
C 2.61533700 1.19625900 -0.35987700
N 3.29552100 -2.44901200 -0.08768900
H 2.38028700 -2.71834100 -0.44201400
C 4.11070600 -3.43695500 0.38800100
O 5.22777300 -3.23847400 0.88114100
O 3.97575000 3.10321400 -0.34756200
H-4.21575000 1.76659800 0.44077000
H-3.82161500 -2.42219700 -0.38448100
H-1.37153700 -2.18649500 -0.38568800
H 5.63850300 -1.14669900 0.14602800
H 5.99647700 1.27182600 0.02873300
H 1.79153900 1.88161700 -0.52759700
C 5.26564900 3.71707100 -0.25701500
H 5.73302600 3.51437000 0.71359800
H 5.92424700 3.37358700 -1.06320800
H 5.09124500 4.78800100 -0.36028000
C-1.80819100 4.84893400 0.16938200
H-0.88209200 5.00298100 -0.39365500
H-2.57038000 5.53945200 -0.19168200
H-1.60857900 5.04971600 1.22499400
C-4.19560500 -0.33793500 0.01692000
N-5.60516400 -0.38458600 0.05751000
C-6.44235000 -1.46210600 -0.07636700
H-6.05682700 0.50654300 0.21452200
O-6.04275400 -2.61452000 -0.26717900
C-7.92199700 -1.15048900 0.03307600
H-8.42426200 -1.54475900 -0.85435400
H-8.32230800 -1.67849900 0.90375700
H-8.14853800 -0.08626000 0.12918400
C 3.54846700 -4.84074700 0.26679400
H 3.55483700 -5.30399400 1.25739100

H-8.48342400 -2.13020200 -0.02372900
H-8.14861200 -0.75293300 1.05446500
H-8.21941800 -0.50414100 -0.69967000
C 3.62485300 -4.83645900 0.05865100
H 3.73195100 -5.35507400 1.01560800
H 2.57532300 -4.85589600 -0.24578200
H 4.22231300 -5.38019000 -0.67907700

Figure S12. Box-Aaa$_Z$ Gas

C 1.17000100 -0.81683400 -0.27179800
N 0.04729800 -0.10436200 0.05254500
H 0.14974500 0.85542800 0.38936900
C-1.29562200 -0.50055500 0.00662900
C-2.30399400 0.48820300 0.17404100
C-3.65431200 0.10963600 0.19490200
C-3.03901000 -2.18171100 -0.19973500
C-1.69471200 -1.83675600 -0.18849200
C-1.94648000 1.93890600 0.32282700
O-0.81810200 2.31503700 0.69279600
N-2.90398400 2.85363700 0.02901100
H-3.76963800 2.55550700 -0.39346600
O 1.12615600 -2.01638200 -0.59714600
C 2.46178500 -0.05626000 -0.19537300
C 3.70752000 -0.73932800 -0.05685400
C 4.88181600 0.02075300 0.04941000
C 4.85324000 1.41352500 -0.01341300
C 3.63838400 2.08445200 -0.18640600
C 2.45865200 1.34412200 -0.27196600
N 3.72332700 -2.14399700 -0.03383400
H 2.82321800 -2.55174400 -0.28155700
C 4.76383200 -2.99413300 0.24815300
O 5.90093700 -2.63603500 0.54758600
O 3.49785200 3.44247100 -0.28725700
H-4.42871800 0.83756300 0.38279600
H-3.31084000 -3.22317300 -0.35666600
H-0.93704800 -2.59182300 -0.33443700
H 5.82215600 -0.49512500 0.18062900
H 5.78900500 1.95393600 0.06436000
H 1.54083800 1.90389000 -0.41347600
C 4.66584400 4.24844400 -0.19512200
H 5.16507400 4.12257200 0.77474500
H 5.37535400 4.02178900 -1.00206300
H 4.32434700 5.27973000 -0.29375100
C-2.63227900 4.28068400 0.14297000
H-1.88004200 4.59974700 -0.58653600
H-3.56138600 4.82701700 -0.02790100
H-2.25235000 4.51066700 1.14147100
C-4.03829900 -1.21829700 -0.00003400
H 2.53505800 -4.87631300 -0.13955400
H 4.21112800 -5.42651700 -0.37740100

Figure S12. Box-Aaa$_Z$ DCM

C 1.16024000 -0.80845300 -0.38931100
N 0.06042400 -0.13008700 0.04224000
H 0.18537300 0.79446100 0.45855800
C-1.29382400 -0.50293300 -0.01742600
C-2.28192300 0.49835400 0.17179000
C-3.63994200 0.14704700 0.17938000
C-3.06170100 -2.15167600 -0.25142400
C-1.71173100 -1.82685800 -0.23703100
C-1.89243400 1.93495400 0.37705100
O-0.79424400 2.25728700 0.88074900
N-2.77576300 2.87858400 -0.00912800
H-3.59979000 2.60867900 -0.52521700
O 1.10861700 -1.96474900 -0.84926500
C 2.45943400 -0.06487700 -0.26410600
C 3.69025200 -0.76277900 -0.10078600
C 4.87045400 -0.01815400 0.02848600
C 4.86205400 1.37677100 -0.02461200
C 3.65771100 2.06266200 -0.21540800
C 2.47100200 1.33395700 -0.33268900
N 3.68397200 -2.17194900 -0.08672600
H 2.81024700 -2.56826600 -0.42758400
C 4.65023800 -3.03494100 0.34963400
O 5.73868700 -2.68292500 0.81794800
O 3.53431300 3.42074500 -0.30612100
H-4.39672600 0.89119900 0.37722300
H-3.35153500 -3.18559600 -0.41842100
H-0.96993200 -2.59543000 -0.39423000
H 5.80510800 -0.54074300 0.17109300
H 5.80222400 1.90561600 0.07218500
H 1.55661400 1.89392900 -0.49576500
C 4.71698800 4.21724300 -0.19748300
H 5.19931100 4.07923200 0.77744800
H 5.42848300 3.98136400 -0.99778700
H 4.38743100 5.25152500 -0.29665700
C-2.49709100 4.29979000 0.16126600
H-1.64229500 4.60818900 -0.44938200
H-3.38027500 4.86358200 -0.13990900
H-2.26811800 4.51651800 1.20774400
C-4.04546400 -1.17406300 -0.03652100
N-5.39466600 -1.58753000 -0.03300400
C-6.53305400 -0.82397300 0.00909500
H-5.53401000 -2.58635900 -0.10513700
O-6.52184400 0.40859200 0.07196000
C-7.83492300 -1.59972100 -0.02860800

N-5.38700000 -1.64174500 0.00999200
C-6.52638700 -0.87104400 -0.04098700
H-5.52220400 -2.64254300 0.00027800
O-6.51505500 0.35648500 -0.07168500
C-7.83206000 -1.64773000 -0.05074000
H-8.41392000 -1.35478200 0.82781300
H-8.40144700 -1.35119500 -0.93579100
H-7.71024200 -2.73475900 -0.05043800
C 4.40606400 -4.47038500 0.16305700
H 4.61755600 -4.93211300 1.13180400
H 3.36360100 -4.65992400 -0.10696700
H 5.06124600 -4.94214800 -0.57497600

Figure S12. Box-Aaa$_Z$ Tol

C 1.16622500 -0.81593100 -0.32365000
N 0.05325600 -0.11689900 0.04543800
H 0.16473300 0.82800300 0.41978500
C-1.29524900 -0.50221600 -0.00762100
C-2.29412000 0.49206000 0.17438200
C-3.64811500 0.12546700 0.18675500
C-3.04999600 -2.16762400 -0.23411300
C-1.70315500 -1.83183300 -0.21982700
C-1.92187400 1.93604700 0.35745900
O-0.80186000 2.28540200 0.78376700
N-2.85009900 2.86558500 0.03578400
H-3.70167900 2.58389900 -0.42548100
O 1.12146100 -1.99994800 -0.70573000
C 2.46078400 -0.06082300 -0.22714600
C 3.70091100 -0.74755400 -0.07101500
C 4.87589400 0.00846200 0.04575800
C 4.85435900 1.40195900 -0.02145700
C 3.64377300 2.07635900 -0.21131000
C 2.46228600 1.33824700 -0.30912900
N 3.70812800 -2.15445800 -0.04494500
H 2.81801300 -2.55806800 -0.33192000
C 4.71994800 -3.01025000 0.30096500
O 5.84295600 -2.65518000 0.66574500
O 3.50938900 3.43321300 -0.31809400
H-4.41471300 0.85962000 0.38301600
H-3.33053800 -3.20487100 -0.39933900
H-0.95298500 -2.59255300 -0.37431600
H 5.81390100 -0.50773100 0.18944700
H 5.79079700 1.93943800 0.06514500
H 1.54491100 1.89498100 -0.46513200
C 4.68349300 4.23735800 -0.22154000
H 5.17195100 4.11401200 0.75312500
H 5.39599100 4.00050800 -1.02171800
H 4.34656900 5.26872100 -0.32960600

H-8.41993100 -1.33644600 0.85698700
H-8.40214200 -1.28058200 -0.90772300
H-7.70611000 -2.68411500 -0.06176400
C 4.29564200 -4.50478200 0.21783800
H 4.36153300 -4.96772700 1.20656300
H 3.30038600 -4.68343800 -0.19594400
H 5.04057600 -4.98570400 -0.42290800

Figure S12. Box-Aaa$_Z$ MeCN

C 1.15806600 -0.80815900 -0.41280400
N 0.06315600 -0.13853000 0.04145900
H 0.19362400 0.77811600 0.47249800
C-1.29330500 -0.50545700 -0.02129100
C-2.27673800 0.49847900 0.17428500
C-3.63644400 0.15375900 0.17703100
C-3.06715700 -2.14568900 -0.26620000
C-1.71582500 -1.82610500 -0.24900000
C-1.87994700 1.93090300 0.39411900
O-0.78931200 2.23996000 0.92418600
N-2.74631400 2.88245600 -0.00646700
H-3.56033300 2.62045700 -0.54243900
O 1.10460400 -1.95337300 -0.89961700
C 2.45826200 -0.06765100 -0.27892900
C 3.68607000 -0.76827100 -0.11135600
C 4.86771500 -0.02650900 0.01938000
C 4.86304800 1.36870700 -0.03276700
C 3.66035500 2.05730200 -0.22513800
C 2.47228000 1.33089900 -0.34683800
N 3.67610900 -2.17819800 -0.09663800
H 2.80988300 -2.57451500 -0.45546800
C 4.62513900 -3.04056200 0.37498000
O 5.69814100 -2.68567300 0.87827400
O 3.53973000 3.41550900 -0.31355000
H-4.38953400 0.90114900 0.37705600
H-3.36096100 -3.17743000 -0.43859600
H-0.97752900 -2.59761500 -0.40893600
H 5.80178800 -0.55012000 0.16217200
H 5.80430400 1.89529000 0.06521900
H 1.55856000 1.89070100 -0.51476300
C 4.72534900 4.21035900 -0.20781300
H 5.20847100 4.07208100 0.76641500
H 5.43430800 3.97131800 -1.00904300
H 4.39758400 5.24501900 -0.30785700
C-2.46251500 4.30146400 0.17454500
H-1.59244000 4.60592600 -0.41614700
H-3.33469000 4.87154400 -0.14568500
H-2.25712000 4.51433400 1.22673600
C-4.04715700 -1.16491700 -0.04673900

C-2.57759700 4.28952800 0.18846000
H-1.79246300 4.61726300 -0.50102500
H-3.49553100 4.84213700 -0.01598400
H-2.24531200 4.50030300 1.20783800
C-4.04214400 -1.19844800 -0.02408000
N-5.39054800 -1.61837200 -0.01932700
C-6.53075800 -0.85189300 -0.02762100
H-5.52657700 -2.61898100 -0.05868700
O-6.52092200 0.37926200 -0.01674600
C-7.83271700 -1.63086800 -0.04532900
H-8.39766700 -1.38021900 0.85715100
H-8.42101300 -1.29981500 -0.90539400
H-7.70423800 -2.71531100 -0.09478000
C 4.35943000 -4.48330200 0.20450600
H 4.50006600 -4.93881900 1.18898500
H 3.33634800 -4.66733200 -0.13321300
H 5.05781700 -4.96664800 -0.48476600

Figure S3. Box$_5$ DCM

C -5.05623900 0.22224600 -0.33203900
N -6.29340200 0.04051500 0.17805100
H -6.77675900 0.82691200 0.60883400
C -7.09978700 -1.11230400 0.10726900
C -8.49941700 -0.95522200 0.20435800
C -9.31350800 -2.08161100 0.20753700
C -8.77865000 -3.36244000 0.08168500
C -7.40014600 -3.51189800 -0.04357700
C -6.57373200 -2.39415600 -0.02362100
C -9.10663300 0.41068100 0.31791600
O -8.50769900 1.35908000 0.84680300
O -9.67390100 -4.38145000 0.09832000
N-10.34246800 0.57131000 -0.18531800
H-10.74807400 -0.16123100 -0.74720400
O -4.40468800 -0.69132600 -0.85862600
C -4.51222400 1.61560100 -0.20238000
C -3.11799800 1.84351600 -0.14466200
C -2.65098400 3.14560300 0.00918500
C -3.53108200 4.21871700 0.08532000
C -4.90359300 4.00267800 -0.00347500
C -5.38051500 2.70182700 -0.14714900
N -2.25668300 0.73736500 -0.26493800
H -2.71786000 -0.06485700 -0.68755700
C -0.99640500 0.59393700 0.20384200
O -0.34667500 1.52744700 0.69398100
C -0.43529700 -0.79171600 0.07483500
C -1.30793500 -1.88751500 0.04714400
C -0.82475100 -3.17795000 -0.11155800
C 0.55229100 -3.38030300 -0.22184700
N-5.39722200 -1.57424900 -0.04779600
C-6.53361500 -0.81103600 0.01691600
H-5.53905500 -2.57139000 -0.13842000
O-6.51998200 0.42080400 0.10889900
C-7.83696100 -1.58250100 -0.03639700
H-8.42745200 -1.32548600 0.84732300
H-8.39670600 -1.25441300 -0.91720100
H-7.71066300 -2.66662600 -0.07871400
C 4.27603000 -4.51042100 0.23676300
H 4.32290000 -4.97333500 1.22655000
H 3.28973800 -4.69055500 -0.19691000
H 5.03387700 -4.99019200 -0.38972000

Figure S3. Box$_5$ Tol

C -5.07401700 0.22734900 -0.38219900
N -6.30432400 0.03976600 0.14687000
H -6.76579500 0.81532700 0.62049900
C -7.10937900 -1.11291000 0.09586900
C -8.49739400 -0.96799200 0.31664000
C -9.30570100 -2.09867900 0.34224700
C -8.78356700 -3.36998300 0.11666400
C -7.42148200 -3.50562400 -0.13516200
C -6.59695100 -2.38664500 -0.13804100
C -9.09869700 0.38786900 0.53253900
O -8.45648100 1.33115400 1.01189000
O -9.67391100 -4.39299500 0.16808700
N-10.38864100 0.54791600 0.17678600
H-10.84216800 -0.16611000 -0.37116300
O -4.42991600 -0.68107900 -0.92496200
C -4.53199700 1.62089100 -0.25166500
C -3.13706700 1.85317800 -0.22229100
C -2.67157700 3.15511900 -0.06247100
C -3.55435300 4.22314100 0.04394000
C -4.92741400 4.00362100 -0.01822600
C -5.40296900 2.70316600 -0.16468600
N -2.27447100 0.75257200 -0.37011900
H -2.73989300 -0.04824600 -0.78898100
C -0.99988300 0.61443700 0.06640800
O -0.34309400 1.55357100 0.53315500
C -0.43841400 -0.77006000 -0.06845000
C -1.31136500 -1.86613000 -0.10110700
C -0.83009600 -3.15510200 -0.26863400
C 0.54613700 -3.35786400 -0.38346300
C 1.42341700 -2.29330500 -0.32118600
C 0.94910000 -0.98508400 -0.15626600
N 1.80608200 0.12502100 -0.05605100
H 1.33753700 0.94176500 0.33225500
C 3.09430800 0.23630900 -0.45622700

C 1.42825000 -2.31373800 -0.16549000	C 3.67863100 1.61363300 -0.33297600
C 0.95188600 -1.00573400 -0.01177700	O 3.75409800 -0.71838100 -0.88468600
N 1.80873300 0.10651900 0.08113500	C 5.07308500 1.79725000 -0.17779500
H 1.34594600 0.91944900 0.48329200	C 5.56879800 3.09160000 -0.03965000
C 3.07694900 0.23140000 -0.37131400	C 4.72239900 4.19349900 -0.07505400
C 3.65783700 1.61056200 -0.25470300	C 3.35468300 4.01816600 -0.26037100
O 3.72392700 -0.71398800 -0.84083900	C 2.84724200 2.72860100 -0.38807900
C 5.05483100 1.80119600 -0.14297600	N 5.90202000 0.66174200 -0.18281300
C 5.54843200 3.09722400 -0.01460200	H 5.43896000 -0.15422200 -0.57658900
C 4.69625200 4.19546600 -0.01448600	C 7.15964300 0.53083400 0.30670300
C 3.32355000 4.01338100 -0.15496200	O 7.81827500 1.48622500 0.73274400
C 2.81930500 2.72127800 -0.27557300	C 7.70277700 -0.86817500 0.28542800
N 5.88966500 0.66957000 -0.18299800	C 6.82494100 -1.95019900 0.30659100
H 5.42121400 -0.14417800 -0.57489200	C 7.28498200 -3.26169600 0.27119600
C 7.15228400 0.53456600 0.28870400	C 8.65504300 -3.49574900 0.21274700
O 7.81456600 1.48255800 0.72668300	C 9.54606700 -2.42972700 0.21211300
C 7.69909700 -0.86198000 0.23378300	C 9.09780500 -1.10982400 0.26398300
C 6.82535400 -1.94610200 0.28071400	O 6.32650800 -4.22728100 0.30108200
C 7.28670200 -3.25691100 0.22027700	O 2.44190300 5.02267300 -0.33410600
C 8.65481400 -3.48670800 0.11216600	O -1.61404500 -4.26326900 -0.32654700
C 9.54165000 -2.41700400 0.08475900	N 9.97982500 -0.01826300 0.30152300
C 9.09243800 -1.09850800 0.15774000	H 9.51561700 0.85824300 0.52641800
O 6.33289000 -4.22419800 0.27732800	C 11.32935200 -0.00704200 0.10431300
O 2.40387300 5.01182200 -0.19037800	O 12.00644300 -0.99214800 -0.15735000
O -1.60645400 -4.28651700 -0.16452000	O -5.87473200 4.97406500 0.04990900
N 9.97168500 -0.00267900 0.16577300	H -10.36368100 -2.02505400 0.56769600
H 9.51851300 0.86880400 0.42869300	H -6.98045200 -4.47783200 -0.31607200
C 11.30268400 0.02224200 -0.12471400	H -5.53982800 -2.49728100 -0.32518500
O 11.96267700 -0.95672300 -0.45394500	H -1.60652500 3.32834600 -0.01847500
O -5.84835100 4.97620100 0.03519500	H -3.15178800 5.22135000 0.16107000
H -10.38697600 -1.99471600 0.33351100	H -6.47843700 2.57097900 -0.21212200
H -6.94973900 -4.49154500 -0.14101900	H -2.37374600 -1.70582900 0.01966300
H -5.50452800 -2.51587900 -0.10930600	H 0.91436300 -4.37003000 -0.51041800
H -1.58649100 3.31722200 0.07079400	H 2.48682300 -2.45822000 -0.40607100
H -3.12751800 5.21679000 0.19905000	H 6.63021400 3.23042000 0.09928600
H -6.45493900 2.57165900 -0.22081100	H 5.15132300 5.18213000 0.03023000
H -2.37078900 -1.72603700 0.16382500	H 1.77806600 2.62964900 -0.53918300
H 0.92280100 -4.39273600 -0.34007000	H 5.75178800 -1.80674600 0.36292200
H 2.49200100 -2.47951100 -0.24587100	H 9.05059900 -4.50331500 0.17891400
H 6.61325100 3.24273400 0.08727300	H 10.60919900 -2.61217100 0.16963900
H 5.12309400 5.18575800 0.08171000	C -9.18878000 -5.70769200 -0.03262500
H 1.74665900 2.61616500 -0.39527500	H -8.74787900 -5.82434800 -1.02960800
H 5.75499100 -1.80270500 0.37766000	H -8.44601200 -5.98040800 0.72619600
H 9.05171600 -4.49281600 0.06006700	H -10.05335900 -6.36506800 0.05734300
H 10.60258100 -2.59844800 0.00315800	C -11.03873100 1.83626700 0.32745800
C -9.17798700 -5.70578500 -0.00632800	H -10.90652600 2.19856300 1.34833000
H -8.65081100 -5.86115700 -0.95441100	H -10.61318800 2.57891400 -0.35490700
H -8.50725600 -5.94734600 0.82589400	H -12.10250800 1.71957600 0.11977100

H-10.05030300 -6.35767100 0.03218000
C-11.00044900 1.86380300 -0.13828500
H-11.01160900 2.23876600 0.88687300
H-10.48042700 2.59480000 -0.76517100
H-12.02464400 1.74806600 -0.49166400
C -5.41608200 6.31840700 0.17928700
H -4.87245600 6.46439500 1.11959700
H -4.77999000 6.62555400 -0.65866500
H -6.32029600 6.92655300 0.18763400
C 2.86652600 6.34632100 -0.07445900
H 3.54508900 6.60699700 -0.89470300
H 1.97997500 6.97805900 -0.12549400
H 3.37457700 6.51176200 0.88245900
C 6.75485900 -5.57513100 0.21935200
H 7.27276300 -5.79297900 -0.72185300
H 7.41233600 -5.82599800 1.05981500
H 5.84842700 -6.17739400 0.27901100
C 11.94399400 1.38668600 0.01436600
H 11.22279000 2.20409700 0.08044400
H 12.56450900 1.39285800 0.91546000
H 12.60142100 1.55091200 -0.84150300
C -3.01293600 -4.10989500 -0.12068700
H -3.33334900 -3.68701500 0.83900000
H -3.36015700 -3.46051700 -0.93275100
H -3.44552200 -5.10339300 -0.23742200

Figure S3. Aaa-Box$_3$-Aaa Tol

C 4.71812634 0.65808205 0.44610803
N 5.99489242 0.53440904 0.01174200
H 6.46902248 1.34772210 -0.38223303
C 6.82978050 -0.59276404 0.06210900
C 8.21880357 -0.40514903 -0.08568901
C 9.07290666 -1.50763111 -0.11021101
C 7.20824150 -2.97171721 0.23510102
C 6.34367346 -1.89237513 0.22969802
C 8.79518861 0.97226107 -0.22069502
O 8.13886161 1.93050014 -0.65251205
N 10.07632372 1.14019508 0.15770801
H 10.55789777 0.39540603 0.63632905
O 4.06260029 -0.29578102 0.88575006
C 4.14417230 2.04035715 0.34147602
C 2.74895420 2.23484416 0.21198502
C 2.25761516 3.52997926 0.07373001
C 3.11182923 4.62584533 0.08720401
C 4.48189632 4.44184132 0.25042402
C 4.98487336 3.14967123 0.37450603
N 1.90972614 1.10778808 0.24128702
H 2.36194517 0.29748602 0.65560605

C -5.44438600 6.31249500 0.20606400
H -4.88176200 6.44702900 1.13750700
H -4.82592300 6.63666200 -0.63945300
H -6.34990700 6.91808300 0.24245000
C 2.90810500 6.35166700 -0.20852000
H 3.61733600 6.60601300 -1.00533700
H 2.02838200 6.98977900 -0.29366700
H 3.38381100 6.52042300 0.76499800
C 6.74680200 -5.57614300 0.26539100
H 7.29696000 -5.80215700 -0.65608800
H 7.37497400 -5.82530500 1.12930800
H 5.83787700 -6.17731200 0.29715000
C 11.96590500 1.35955700 0.25811000
H 12.66166300 1.51252500 -0.56916600
H 11.24446600 2.17941900 0.28318900
H 12.54684900 1.37325100 1.18499700
C -3.01751900 -4.08239100 -0.28647900
H -3.34206500 -3.66324600 0.67404800
H -3.36142400 -3.42647800 -1.09525600
H -3.45266200 -5.07441300 -0.40993000

Figure S3. Box$_5$ MeCN

C -5.05007200 0.22569500 -0.35159500
N -6.27652600 0.03923100 0.18034300
H -6.75189700 0.82245900 0.62584000
C -7.08496100 -1.11288900 0.11654800
C -8.48224000 -0.95568400 0.24343300
C -9.29649900 -2.08194600 0.25295800
C -8.76450000 -3.36192000 0.10570700
C -7.38876600 -3.51109800 -0.04777600
C -6.56185800 -2.39355700 -0.03506400
C -9.08609300 0.40949600 0.38320900
O -8.47420200 1.35192600 0.91046200
O -9.65941200 -4.38083700 0.13180900
N-10.33054400 0.57733300 -0.09221000
H-10.75363000 -0.15093300 -0.64689800
O -4.40675400 -0.68153000 -0.89932800
C -4.50502900 1.61857400 -0.21987800
C -3.11071600 1.84633500 -0.17527400
C -2.64212300 3.14802100 -0.02361600
C -3.52117200 4.22118200 0.06554100
C -4.89466300 4.00486900 -0.00799500
C -5.37283700 2.70421300 -0.15148100
N -2.25036300 0.74000500 -0.30623500
H -2.71356800 -0.05945700 -0.73199100
C -0.99312600 0.59067100 0.16722000
O -0.34407900 1.51938600 0.66817600
C -0.43292000 -0.79469300 0.03057300

C 0.67773905 0.95700207 -0.30456902	C -1.30595700 -1.88993300 -0.00057700
O 0.03295600 1.89513314 -0.78670106	C -0.82280500 -3.18035200 -0.16267200
C 0.15235801 -0.44680103 -0.27667302	C 0.55433800 -3.38253700 -0.27360100
C 1.05133508 -1.52284511 -0.26225302	C 1.43041900 -2.31605100 -0.21436200
C 0.58998104 -2.82932020 -0.21860102	C 0.95418600 -1.00866400 -0.05709300
C -0.78589506 -3.06881822 -0.22320902	N 1.81139600 0.10323400 0.03938600
C -1.68492212 -2.02200614 -0.26431002	H 1.34953300 0.91427400 0.44646400
C -1.22842109 -0.69871205 -0.28669102	C 3.07737900 0.23194500 -0.41693500
N -2.11944215 0.38933403 -0.36274603	C 3.65622800 1.61118500 -0.29345900
H -1.69585212 1.22309009 -0.76009306	O 3.72460400 -0.70931300 -0.89515300
C -3.35868224 0.46628503 0.17699601	C 5.05173400 1.80259100 -0.17118500
C -4.03615429 1.79790013 0.03058900	C 5.54393500 3.09872100 -0.03957300
O -3.90809728 -0.48710403 0.73751505	C 4.69132700 4.19674400 -0.04398500
C -5.44693837 1.88299213 -0.02836000	C 3.31940300 4.01365300 -0.19294600
C -6.03704043 3.13346222 -0.19462201	C 2.81689500 2.72113500 -0.31874100
C -5.26474838 4.28684731 -0.27529902	N 5.88815400 0.67147000 -0.20542700
C -3.87885028 4.20873630 -0.18250201	H 5.42788700 -0.13992400 -0.61081500
C -3.27773524 2.96205622 -0.03161600	C 7.13507300 0.53066200 0.30476100
N -6.19030043 0.69552305 0.09718601	O 7.78326300 1.47397700 0.77287300
H -5.63689041 -0.06714501 0.48439203	C 7.68273300 -0.86525800 0.25410300
C -7.48180153 0.46397203 -0.23844302	C 6.80698600 -1.94898200 0.27151600
O -8.24569959 1.35245809 -0.63509504	C 7.26796400 -3.25994800 0.21539800
C -7.93107556 -0.95931107 -0.07101001	C 8.63850500 -3.49033700 0.14252600
C -6.98783151 -1.98870314 -0.14793701	C 9.52644400 -2.42120500 0.14522100
C -8.69708863 -3.60206926 0.31387602	C 9.07742800 -1.10194900 0.21312100
C -9.64821668 -2.60117219 0.37043703	O 6.31206500 -4.22655500 0.24116400
C -9.29025965 -1.26483409 0.16038901	O 2.39932700 5.01096900 -0.23219600
O -3.03291722 5.27210038 -0.22470102	O -1.60401900 -4.28859100 -0.21809600
O 1.39630210 -3.92390228 -0.18406001	N 9.95821700 -0.00871500 0.25134700
N-10.23283572 -0.22918802 0.16833601	H 9.50142100 0.86544300 0.50112900
H -9.85383771 0.65247605 -0.17152801	C 11.29520300 0.02145700 -0.01799100
C-11.54989482 -0.27551002 0.52915004	O 11.96242400 -0.95638900 -0.33474400
O-12.12575090 -1.27284109 0.94014707	O -5.83909700 4.97765600 0.04584900
O 5.40067637 5.44047638 0.30402402	H-10.36740200 -1.99590300 0.39947800
H 10.13114272 -1.38276110 -0.28016002	H -6.94039900 -4.48984300 -0.16168400
H 6.80178750 -3.97104529 0.36776203	H -5.49462300 -2.51533400 -0.14198500
H 5.28326438 -2.04279515 0.36063503	H -1.57705600 3.32019700 0.02574100
H 1.19451509 3.67509126 -0.04886800	H -3.11660900 5.21903400 0.17721300
H 2.68844219 5.61688539 -0.01682900	H -6.44776200 2.57267300 -0.21448900
H 6.05690945 3.04570222 0.50147004	H -2.36881000 -1.72850800 0.11696300
H 2.11474815 -1.33056710 -0.30174402	H 0.92509600 -4.39465000 -0.39396200
H -1.13833208 -4.09472630 -0.21389402	H 2.49419100 -2.48209500 -0.29406900
H -2.74556620 -2.22868016 -0.29120902	H 6.60804700 3.24545900 0.06858600
H -7.11248852 3.19716423 -0.26203302	H 5.11680700 5.18728800 0.05503200
H -5.76462443 5.23947738 -0.39811603	H 1.74530800 2.61424700 -0.44628100
H -2.19590916 2.93641421 0.04721000	H 5.73474400 -1.80389600 0.34243800
H -5.95331544 -1.77707113 -0.37507203	H 9.03592000 -4.49647400 0.09571200
H -9.00753766 -4.63175834 0.47191803	H 10.58876000 -2.60475900 0.09190300

H-10.68119877 -2.83624520 0.57676604
C -3.59554926 6.55976045 -0.38028703
H -2.75728520 7.25658952 -0.39202803
H -4.14944930 6.64527650 -1.32285709
H -4.26249831 6.81062851 0.45338403
C 4.94257836 6.77225750 0.17312801
H 4.45294132 6.93507449 -0.79436106
H 4.24822431 7.03927748 0.97877807
H 5.82870944 7.40363052 0.23811202
C-12.28090090 1.03850207 0.34476702
H-11.61792383 1.90665814 0.36980803
H-12.79617492 1.02506107 -0.62096304
H-13.03561291 1.12949508 1.12698308
C 2.79297820 -3.71123827 -0.10502401
H 3.17745423 -3.21531623 -1.00493907
H 3.05824722 -3.10961422 0.77284506
H 3.24446123 -4.70019334 -0.02058800
C 10.70435974 2.44486117 0.07244901
H 10.23142372 3.15623622 0.75649006
H 11.75932883 2.34258417 0.32683002
H 10.61240075 2.83795720 -0.94195307
C 8.58182064 -2.79866420 0.05808500
N 9.40040768 -3.94226328 0.04434400
C 10.77051076 -4.00734529 0.04945800
H 8.90995562 -4.82513935 0.07799401
O 11.49379785 -3.02403822 0.03749700
C -7.35945554 -3.31465624 0.04217100
N -6.45002047 -4.39249431 -0.00075800
C -5.15016337 -4.38966432 -0.41938903
H -6.82407147 -5.27938038 0.30405002
O -4.58208233 -3.40069924 -0.85888906
C -4.42727432 -5.71123341 -0.27518302
H -5.08934937 -6.56511345 -0.11247901
H -3.83392527 -5.87956744 -1.17552908
H -3.73695627 -5.63807243 0.57061704
C 11.31541479 -5.41952937 0.06749000
H 12.40336691 -5.37066239 0.07437501
H 10.97323677 -5.96078943 0.95515007
H 10.98548680 -5.97498842 -0.81602906

Figure 2. Aaa_Z-Box_3-Aaa_Z Gas

C -4.73572034 0.65477805 -0.42910403
N -6.02750344 0.53624204 -0.03294900
H -6.51338849 1.35003810 0.34867202
C -6.85284151 -0.59409004 -0.07669401
C -8.24027758 -0.41682903 0.10551501
C -9.08671268 -1.52455711 0.13888201
C -8.59455762 -2.80849220 -0.06354200
C -9.16603000 -5.70474000 0.00216400
H -8.65911400 -5.84926000 -0.95840000
H -8.47800000 -5.95520000 0.81724700
H-10.03766100 -6.35671300 0.05189900
C-10.98841100 1.86840400 -0.01406400
H-10.99232600 2.22425300 1.01813800
H-10.47361600 2.61072600 -0.63172700
H-12.01502300 1.75876100 -0.36171300
C -5.40518700 6.32088200 0.18575700
H -4.84992600 6.46568600 1.11920600
H -4.77989800 6.62829000 -0.65990400
H -6.30946000 6.92852600 0.20592100
C 2.86020300 6.34704100 -0.11368100
H 3.54278100 6.60777000 -0.93025700
H 1.97314300 6.97748100 -0.16954600
H 3.36275300 6.51235900 0.84593000
C 6.73493800 -5.57856700 0.19139700
H 7.27832300 -5.79397400 -0.73569600
H 7.36886900 -5.83134900 1.04894900
H 5.82685600 -6.18024400 0.22462700
C 11.89921100 1.40506100 0.10169300
H 11.47000600 2.07532800 -0.64942400
H 11.69967900 1.83648100 1.08705200
H 12.97543000 1.33533800 -0.05253300
C -3.01137500 -4.11374000 -0.16695800
H -3.32699500 -3.69311400 0.79503500
H -3.36262400 -3.46361500 -0.97651700
H -3.44358300 -5.10723600 -0.28364400

Figure S3. Aaa-Box_3-Aaa DCM

C 4.70255634 0.66044205 0.42260803
N 5.97538541 0.53504404 -0.01621600
H 6.45357648 1.34938510 -0.40166803
C 6.81450447 -0.59031804 0.05064600
C 8.20636859 -0.39426203 -0.03717200
C 9.06675867 -1.49253511 -0.04570300
C 7.19337554 -2.97043121 0.19010801
C 6.32626448 -1.89326614 0.17215401
C 8.77801464 0.98858707 -0.13059801
O 8.14167757 1.93978614 -0.61115104
N 10.02495971 1.16734108 0.33714802
H 10.47642374 0.42579103 0.84985606
O 4.04936429 -0.28899902 0.87710606
C 4.12488030 2.04087215 0.30620702
C 2.73032620 2.23048116 0.17035201
C 2.23448616 3.52341525 0.02945000
C 3.08409522 4.62326133 0.04210300
C 4.45473732 4.44367532 0.20820802

C -7.22595751 -2.97200521 -0.27769602	C 4.96141636 3.15292123 0.33880202
C -6.36546048 -1.89024414 -0.27577202	N 1.89482913 1.09912408 0.20035301
C -8.82321866 0.95482107 0.25900002	H 2.34808417 0.29380502 0.62467405
O -8.15356258 1.92763114 0.62850605	C 0.67601305 0.93607007 -0.36562303
N-10.13212975 1.10150108 -0.03383700	O 0.03584100 1.86341014 -0.87681906
H-10.62984878 0.34630002 -0.47596903	C 0.15259401 -0.46873703 -0.32372902
O -4.06879129 -0.30796602 -0.82940906	C 1.05248208 -1.54317511 -0.30135402
C -4.16517530 2.03756215 -0.33175402	C 0.59172704 -2.85064021 -0.24774402
C -2.76928520 2.23520916 -0.20262901	C -0.78444606 -3.09090822 -0.24875002
C -2.28199116 3.53246226 -0.06335000	C -1.68372312 -2.04385615 -0.29724402
C -3.13993123 4.62430733 -0.07838701	C -1.22788109 -0.72113005 -0.32999402
C -4.50902733 4.43700332 -0.24318102	N -2.12020315 0.36683103 -0.41199403
C -5.00910236 3.14506322 -0.36649903	H -1.70049712 1.19484709 -0.82531006
N -1.92964514 1.11191708 -0.22848202	C -3.34428124 0.45805603 0.15661801
H -2.38920917 0.29442502 -0.61951504	C -4.01769529 1.79116313 0.01116500
C -0.67485405 0.97838407 0.27273902	O -3.88530128 -0.48580003 0.74349006
O -0.01649400 1.93160614 0.70257805	C -5.42824238 1.88398913 -0.02588900
C -0.14790901 -0.42378303 0.26310402	C -6.01401741 3.13747322 -0.18457101
C -1.04646308 -1.50092011 0.27192702	C -5.23747537 4.28682431 -0.28270302
C -0.58506404 -2.80618820 0.23939102	C -3.85004528 4.20094430 -0.21440502
C 0.79039606 -3.04537122 0.23438702	C -3.25388323 2.95082721 -0.06819100
C 1.68980912 -1.99951814 0.25328402	N -6.17681943 0.70067505 0.11467201
C 1.23350409 -0.67495205 0.26377002	H -5.62614038 -0.06212800 0.50430504
N 2.12068015 0.41482803 0.31887102	C -7.46627156 0.46758803 -0.22611602
H 1.68784012 1.25778909 0.68614105	O -8.22766057 1.35189510 -0.63740305
C 3.37792724 0.47147503 -0.18149101	C -7.91647857 -0.95346607 -0.04818000
C 4.05784229 1.80371313 -0.05316500	C -6.97703052 -1.98439314 -0.14554301
O 3.94061628 -0.50052704 -0.69362705	C -8.68003663 -3.59675026 0.34117002
C 5.46955742 1.88335913 0.00940800	C -9.62689269 -2.59254219 0.41994903
C 6.06453542 3.13280022 0.16815601	C -9.27113365 -1.25592309 0.20858701
C 5.29580540 4.28887431 0.23368902	O -2.99932922 5.25776938 -0.27612102
C 3.91067528 4.21555330 0.13393301	O 1.39692310 -3.94366629 -0.20628201
C 3.30395224 2.97152921 -0.00699800	N-10.21064372 -0.21635802 0.23741702
N 6.20419446 0.69144205 -0.10140401	H -9.84146671 0.66239505 -0.11910301
H 5.63558440 -0.07336901 -0.46518003	C-11.51154883 -0.25122202 0.64954005
C 7.50491451 0.46564903 0.20031901	O-12.07325789 -1.24398309 1.09542308
O 8.28101460 1.36388710 0.54878304	O 5.36999637 5.44455840 0.25902202
C 7.94930556 -0.96207107 0.05896500	H 10.13081770 -1.35837810 -0.16615901
C 7.00100453 -1.98763814 0.12863101	H 6.78591848 -3.97342529 0.28170602
C 7.37293755 -3.31481424 -0.04351900	H 5.26193838 -2.05184315 0.25288702
C 8.71387365 -3.61015326 -0.28679702	H 1.17102109 3.66615926 -0.09307601
C 9.67022267 -2.61518519 -0.33498402	H 2.65764719 5.61260038 -0.06422800
C 9.31206868 -1.27544109 -0.14348401	H 6.03296243 3.05079722 0.47222803
O 3.06848622 5.28479738 0.16071501	H 2.11585115 -1.34982510 -0.34187402
O -1.39289410 -3.90207728 0.22500602	H -1.13661808 -4.11690230 -0.23001602
N 10.25684873 -0.24544002 -0.14363301	H -2.74464320 -2.25063716 -0.32297402
H 9.86390070 0.64557605 0.15805101	H -7.09013549 3.20913423 -0.23144002
C 11.59107782 -0.31287402 -0.43885703	H -5.73402640 5.24186738 -0.39890303

O 12.18008687 -1.32296309 -0.78846006
O -5.43080037 5.43466638 -0.29876102
H-10.14076174 -1.41244510 0.33889502
H -6.81914350 -3.96670429 -0.44019703
H -5.30817238 -2.02998615 -0.43672303
H -1.21941509 3.67699726 0.06225600
H -2.71974220 5.61629441 0.02655200
H -6.08086144 3.04151522 -0.49282704
H -2.10899215 -1.30896710 0.32029302
H 1.14194908 -4.07106630 0.23555202
H 2.75018420 -2.20573716 0.27143202
H 7.13980851 3.18928523 0.24167302
H 5.79870440 5.24029438 0.35090303
H 2.22256116 2.95221921 -0.08909301
H 5.96266441 -1.77405913 0.33590902
H 9.02312764 -4.64265133 -0.42924003
H 10.70725879 -2.85155121 -0.51895204
C 12.31564488 1.00600707 -0.25847702
H 11.68717186 1.86705613 -0.49680404
H 12.64016294 1.09835808 0.78222706
H 13.19995997 1.00317507 -0.89427106
C 3.63599126 6.56735446 0.30790802
H 2.80359820 7.27110051 0.30817402
H 4.18548330 6.66222647 1.25256909
H 4.31088331 6.81067148 -0.52205204
C -4.97598336 6.76414548 -0.16736201
H -4.48701933 6.93125351 0.80004906
H -4.28030031 7.03598351 -0.97064507
H -5.86132443 7.39619653 -0.23314802
C -2.78653120 -3.68674927 0.15122901
H -3.16916023 -3.18229123 1.04748508
H -3.05808422 -3.08993122 -0.72823105
H -3.24299023 -4.67420834 0.07705101
C 5.14998637 -4.37929132 0.38414103
O 4.57506233 -3.38999424 0.80688106
C 4.42556932 -5.70039443 0.23014802
H 5.08699737 -6.56053548 0.10421501
H 3.79955028 -5.85034643 1.11014908
H 3.76742827 -5.63482238 -0.64038804
C-10.78260675 -4.00815729 -0.07682701
O-11.49966685 -3.02208322 -0.06750600
C-11.36144982 -5.40487137 -0.14522001
H-11.74577586 -5.57446840 -1.15419308
H-10.64686776 -6.19456946 0.09649701
H-12.20568889 -5.45875638 0.54247204
C-10.76597079 2.40083117 0.07036801
H-10.44462177 3.07075822 -0.73325305
H-11.84744288 2.27265117 0.02329000
H -2.17119415 2.91768821 -0.00542300
H -5.94708141 -1.77144013 -0.39049503
H -8.98977863 -4.62605733 0.49982404
H-10.65572978 -2.82882520 0.64416105
C -3.55715525 6.55229947 -0.42110503
H -2.71417919 7.24248250 -0.44842003
H -4.12748730 6.63999848 -1.35288310
H -4.20576830 6.80537050 0.42539703
C 4.90669135 6.77792547 0.13060001
H 4.41660332 6.93939951 -0.83624906
H 4.21230930 7.03925653 0.93724407
H 5.79095042 7.41139053 0.19657301
C-12.24560690 1.06146208 0.47899903
H-11.58020281 1.92771414 0.46930803
H-12.79638590 1.03885407 -0.46674303
H-12.96987592 1.16470609 1.28804509
C 2.79748620 -3.73349327 -0.14121901
H 3.17241023 -3.24455123 -1.04824908
H 3.07017822 -3.12821423 0.73130705
H 3.24758124 -4.72232234 -0.05432100
C 10.65193876 2.47508918 0.29816602
H 10.13017571 3.18215523 0.95039807
H 11.68634582 2.37619317 0.62598005
H 10.63129275 2.86969521 -0.71966305
C 8.57311361 -2.78912520 0.07359301
N 9.39028569 -3.93246029 0.07028101
C 10.75805578 -4.00453429 0.06701800
H 8.89680562 -4.81442235 0.09908901
O 11.48697384 -3.02274522 0.04829000
C -7.34664353 -3.31131624 0.04503600
N -6.44048046 -4.38920831 -0.02233600
C -5.15717337 -4.38886432 -0.48589704
H -6.80577649 -5.27694238 0.29258502
O -4.60446833 -3.39674025 -0.94488807
C -4.43118732 -5.70909442 -0.36453503
H -5.09575337 -6.56725249 -0.24256602
H -3.81650327 -5.84883944 -1.25510109
H -3.76258727 -5.66044340 0.50056704
C 11.29705080 -5.41780441 0.08632201
H 12.38537891 -5.37532439 0.08895201
H 10.95409680 -5.95447545 0.97611607
H 10.95967581 -5.97374745 -0.79378406

Figure 6. Neb Gas

C-1.39909210 0.45239403 0.02994800
N-2.12116516 1.64464412 0.16220201
C-0.00495500 0.45195403 -0.06911100
C-1.67422912 2.94325721 0.10773001

H-10.49559075 2.86307920 1.02032307
N -9.41267369 -3.95435128 -0.04939600
H -8.92407063 -4.83527835 -0.08745301
N 6.45787446 -4.39001831 -0.00977200
H 6.83260350 -5.27793238 -0.30478502

Figure S3. Aaa-Box$_3$-Aaa MeCN

C 4.69823034 0.66727305 0.42886203
N 5.96739145 0.53702504 -0.01689900
H 6.44581747 1.34847310 -0.40749303
C 6.80675949 -0.58834704 0.05987100
C 8.19898757 -0.39268203 -0.01531700
C 9.05949963 -1.49124011 -0.01241100
C 7.18208554 -2.96837721 0.20347201
C 6.31628446 -1.89047114 0.17750201
C 8.77022463 0.99034007 -0.10889201
O 8.14052561 1.93602414 -0.61060804
N 10.00689471 1.17517409 0.38071803
H 10.44873575 0.43900003 0.90952606
O 4.04716229 -0.27644602 0.89858406
C 4.11966629 2.04652715 0.30146002
C 2.72513219 2.23401616 0.16570301
C 2.22759616 3.52560025 0.01927800
C 3.07592422 4.62669633 0.02400000
C 4.44719332 4.44904732 0.18790001
C 4.95516936 3.15932423 0.32604302
N 1.89096714 1.10125808 0.20218801
H 2.34477117 0.29973902 0.63356404
C 0.67765205 0.93073907 -0.37138103
O 0.03898400 1.85198513 -0.89636907
C 0.15609101 -0.47471503 -0.32118102
C 1.05722407 -1.54774311 -0.29267702
C 0.59779504 -2.85595420 -0.23384002
C -0.77827706 -3.09764422 -0.23494502
C -1.67843612 -2.05133615 -0.28903702
C -1.22413009 -0.72845905 -0.32740002
N -2.11766715 0.35860903 -0.41463803
H -1.69845612 1.18476308 -0.83230506
C -3.33878924 0.45406503 0.15884001
C -4.01024529 1.78780213 0.01279600
O -3.87914828 -0.48730704 0.75126106
C -5.42051740 1.88372513 -0.01991200
C -6.00413344 3.13859423 -0.17585801
C -5.22575737 4.28645431 -0.27743802
C -3.83802628 4.19739030 -0.21493502
C -3.24423323 2.94586121 -0.06984800
N -6.17186342 0.70210505 0.12308401
H -5.62414142 -0.06084500 0.51603704
H-3.11352822 1.47537611 0.34368502
C-2.12358215 -0.77453105 0.00921800
C 0.73496405 -0.74388505 -0.17990601
H 0.47743603 1.41322810 -0.04201300
C-2.76927320 3.97178129 0.35578403
O-0.51270004 3.28426224 -0.08891501
C-1.36436010 -1.95624114 -0.03987000
C-3.60732826 -0.80583306 0.04327800
C 0.01482700 -1.96575814 -0.12411101
H-3.76560527 3.61516926 0.08063501
H-2.52913318 4.87401235 -0.20849501
H-2.77962320 4.23202630 1.41989110
H-1.87072313 -2.91446621 0.03051100
N-4.21549930 -1.96798814 -0.35107403
O-4.30789431 0.15567701 0.41026903
C-5.66506841 -2.05195615 -0.41560003
H-3.68213826 -2.61745819 -0.90674806
H-5.95885042 -3.10083422 -0.49874904
H-6.08900442 -1.62910312 0.49633704
H-6.07681144 -1.49387711 -1.26620909
N 2.11323815 -0.71716305 -0.33624802
C 2.78091220 0.59535004 -0.34590402
C 2.88400021 -1.92730414 -0.05547700
H 2.70403319 1.07895508 0.64192705
H 2.21585416 1.22595009 -1.03767608
C 4.24876131 0.61611504 -0.80015606
H 3.88506128 -1.79707913 -0.46488903
H 2.45521418 -2.76086120 -0.62049304
C 2.96443321 -2.28133817 1.43535610
H 4.42745132 1.61485712 -1.21802409
H 4.39287132 -0.07897101 -1.63732012
C 5.30332337 0.38105403 0.29201602
H 3.55817125 -3.18923423 1.58636011
H 3.42966525 -1.46981110 2.00292715
H 1.96637614 -2.44924017 1.84911213
H 5.18055737 -0.60522304 0.75217205
H 5.14061537 1.11017008 1.09663508
H 0.52400004 -2.91961121 -0.14242701
C 6.73228848 0.51297504 -0.24288102
H 7.47231556 0.36378603 0.54963004
H 6.92922348 -0.22702502 -1.02714407
H 6.90159350 1.50509411 -0.67650405

Figure 6. Feb Gas

C-1.67194512 -0.53122504 -0.19006801
N-3.03828722 -0.61103504 0.09396601
C-0.90620406 -1.66398312 -0.51582604
C-3.87328028 -1.70141312 -0.00195900

C -7.45818954 0.46830303 -0.22860902	H-3.41385125 0.26620102 0.45561303
O -8.21542160 1.35086510 -0.65109705	C-1.02441007 0.73088005 -0.15015101
C -7.91029159 -0.95185107 -0.05022800	C 0.44360703 -1.53456511 -0.76796905
C -6.97196852 -1.98368014 -0.14843401	H-1.37552810 -2.63358319 -0.59376004
C -8.67750362 -3.59559126 0.33083102	C-5.29171338 -1.42506610 0.47482503
C -9.62277669 -2.58979219 0.41154903	O-3.53300826 -2.80854220 -0.39991403
C -9.26532365 -1.25285109 0.20490101	C 0.36615003 0.79503806 -0.35332403
O -2.98518221 5.25160138 -0.28123402	C-1.79854613 1.98008314 0.12075701
O 1.40324110 -3.94766028 -0.18702901	C 1.14983108 -0.31760402 -0.67942105
N-10.20338473 -0.21158102 0.23613302	H-5.58162742 -0.37493503 0.38045703
H -9.83550473 0.66653705 -0.12280801	H-5.37614837 -1.71163213 1.52878211
C-11.50000182 -0.24219602 0.66069405	H-5.97602841 -2.05114115 -0.09935901
O-12.05841785 -1.23347409 1.11563708	H 0.86624206 1.74332013 -0.19855601
O 5.36175237 5.45062838 0.22971002	N-1.23892009 3.15252723 -0.30159202
H 10.12482371 -1.35748210 -0.12301701	O-2.89769321 1.98870514 0.69803705
H 6.77273948 -3.97088428 0.28902202	C-1.95058914 4.41046132 -0.14162601
H 5.25112738 -2.04903815 0.24595602	H-0.50170104 3.10686922 -0.98724107
H 1.16364109 3.66719327 -0.10058201	H-1.24972009 5.23739838 -0.27479702
H 2.64840319 5.61508143 -0.08639101	H-2.77224220 4.51428533 -0.86109706
H 6.02676741 3.05797122 0.45989103	H-2.37653417 4.45424032 0.86166206
H 2.12046615 -1.35315110 -0.33272402	N 2.53192518 -0.26593502 -0.91489606
H -1.12949508 -4.12393330 -0.21158902	C 3.38421925 -1.19209009 -0.13984101
H -2.73920320 -2.25884916 -0.31529002	H 4.41914432 -0.87241706 -0.28371302
H -7.08029449 3.21352223 -0.21753902	H 3.30240924 -2.19674916 -0.56245004
H -5.72075140 5.24255538 -0.39121903	C 3.11951122 1.02027307 -1.29088609
H -2.16148315 2.90991921 -0.00985200	H 3.42646525 1.61342211 -0.41293003
H -5.94158644 -1.77047213 -0.39103203	H 2.34137217 1.59411711 -1.80270913
H -8.98881367 -4.62488533 0.48540804	C 4.29968131 0.87468307 -2.25595516
H-10.65203475 -2.82628820 0.63339805	H 4.64266033 1.86496613 -2.57342118
C -3.54045926 6.54916547 -0.42007003	H 5.15340037 0.36509903 -1.80066413
H -2.69550920 7.23664753 -0.45024503	H 3.99774729 0.30690902 -3.14054723
H -4.11543629 6.64019349 -1.34841310	C 3.08216222 -1.24786309 1.36467310
H -4.18343630 6.80124948 0.43068903	H 3.77207827 -1.97301514 1.81778513
C 4.89637935 6.78417849 0.09884501	H 2.07420015 -1.65251912 1.52060711
H 4.40269332 6.94169752 -0.86660606	C 3.20200723 0.09958301 2.08422915
H 4.20483730 7.04657748 0.90726306	H 2.48727918 0.80607706 1.64555212
H 5.78028940 7.41850056 0.15973401	H 4.20204630 0.51980704 1.90800814
C-12.23407787 1.07029908 0.49286604	C 2.94314121 -0.01046800 3.58934326
H-11.56789584 1.93565114 0.47362503	H 3.02842222 0.96203307 4.08485329
H-12.79387295 1.04497308 -0.44749403	H 1.93687114 -0.39762103 3.78476427
H-12.95025191 1.17767708 1.30856710	H 3.65758827 -0.69220905 4.06463829
C 2.80506020 -3.73763227 -0.12651701	F 1.12625508 -2.65438519 -1.09662508
H 3.17683723 -3.25210824 -1.03638708	
H 3.07954222 -3.12982123 0.74350505	**Figure 6. Ceb Gas**
H 3.25528824 -4.72597834 -0.03715700	C-1.68516612 -0.60824605 -0.24608102
C 10.63391477 2.48305418 0.34369802	N-3.03661322 -0.81701106 0.06554300
H 10.09830971 3.19497923 0.97918907	C-0.86031906 -1.62232012 -0.76715705
H 11.66094885 2.38760417 0.69442705	C-3.82298128 -1.90814114 -0.21233602

H 10.63527878 2.86884720 -0.67776705
C 8.56341761 -2.78774520 0.10199301
N 9.37770868 -3.93270728 0.11148601
C 10.74143179 -4.01381429 0.02566600
H 8.88313163 -4.81158235 0.18663401
O 11.47273083 -3.03758122 -0.07291701
C -7.34310053 -3.31097224 0.03760200
N -6.43790045 -4.38915032 -0.03181300
C -5.15763537 -4.38982432 -0.50194604
H -6.80301048 -5.27728438 0.28287802
O -4.60689733 -3.39639824 -0.96339707
C -4.43127332 -5.70964440 -0.38565903
H -5.09541037 -6.56701245 -0.25783502
H -3.82315427 -5.85034744 -1.28061509
H -3.75628427 -5.66057739 0.47445403
C 11.27458083 -5.42840637 0.06849100
H 12.35886289 -5.39624240 -0.03001900
H 11.01187380 -5.91175645 1.01439907
H 10.85426677 -6.02875341 -0.74389705

Figure 6. Box Gas

C-1.46408910 -0.67787205 0.02494100
N-2.83714820 -0.93422707 0.14919901
C-0.51327904 -1.70126912 -0.10944001
C-3.50318625 -2.12831215 0.01109100
H-3.37015124 -0.10440801 0.40691103
C-1.01116107 0.67098205 0.04020300
C 0.84805906 -1.41840510 -0.18795401
H-0.86106306 -2.72323619 -0.15126901
C-4.99831636 -2.03150015 0.28115402
O-2.97560921 -3.19648923 -0.27673002
C 0.36334003 0.93098407 -0.01104900
C-1.98484214 1.80876913 0.10879001
C 1.30037709 -0.09596101 -0.12413801
H 1.54466711 -2.24236616 -0.28392902
H-5.39102836 -1.01326807 0.21639002
H-5.20150138 -2.42008217 1.28459609
H-5.51848139 -2.67251719 -0.43298303
H 0.74294805 1.94333414 0.07456401
N-1.55647111 3.00853222 -0.37678103
O-3.12852622 1.69517912 0.57603604
C-2.44435017 4.15942530 -0.40461903
H-0.73076505 3.02536622 -0.95418007
H-1.85040413 5.06711336 -0.53104304
H-3.17848023 4.09301529 -1.21703409
H-2.99078922 4.21133730 0.53811404
O 2.60932619 0.29318402 -0.16199601
C 3.62089426 -0.71142405 -0.21903602
H-3.44198325 -0.04305200 0.59008304
C-1.10863608 0.66539605 -0.02594100
C 0.47965503 -1.39471510 -1.03013807
H-1.29850409 -2.58938819 -0.96755807
C-5.23932338 -1.80087013 0.33670302
O-3.45170025 -2.90534521 -0.82126506
C 0.26418002 0.86158306 -0.26252402
C-1.94441014 1.80833413 0.46042403
C 1.09356608 -0.14627501 -0.77869306
H-5.53046038 -0.78312806 0.60986504
H-5.32249340 -2.43825317 1.22312109
H-5.92911145 -2.19059116 -0.41452103
H 0.69365405 1.81157013 0.02497700
N-1.48879411 3.06340322 0.17205801
O-3.00227021 1.66011412 1.09200808
C-2.26677717 4.23548331 0.53791704
H-0.78770906 3.15871523 -0.54579804
H-1.62802912 5.12003137 0.48720404
H-3.13288623 4.37817831 -0.12031501
H-2.63741719 4.11365830 1.55667011
N 2.45160318 0.06389200 -1.04976508
C 3.36367624 -1.07968908 -0.93586007
H 4.35957031 -0.74307805 -1.22491309
H 3.08849122 -1.83516613 -1.68118812
C 2.96870022 1.42416911 -0.89970406
H 2.98966222 1.74389012 0.15669801
H 2.26032416 2.08874515 -1.40911010
C 4.34813731 1.66106812 -1.51370111
H 4.55452233 2.73566720 -1.51484811
H 5.15084637 1.17924208 -0.94899307
H 4.38667932 1.30535010 -2.54784118
C 3.43095325 -1.73104512 0.45567603
H 4.15897530 -2.55297018 0.40877103
H 2.46346318 -2.18876716 0.69429105
C 3.81393128 -0.76019405 1.57753511
H 3.04143022 0.01396100 1.65765512
H 4.74706934 -0.24240402 1.31419010
C 3.97455529 -1.45720211 2.93135721
H 4.23360030 -0.74567105 3.72196827
H 3.04574622 -1.95898014 3.22488323
H 4.76296534 -2.21753416 2.89345321
H 1.06153908 -2.21417716 -1.43442511

Figure 5. Dmx Gas

C 0.44643614 0.57796211 0.02184410
N 1.57549927 1.39688711 0.13261411
C-0.84508292 1.11887522 -0.10022391
C 1.66016836 2.76729420 0.06002310

H 3.51386025 -1.30030409 -1.14199108
H 3.51993125 -1.39902210 0.63527704
C 4.98160936 -0.01946800 -0.17879201
H 5.00888236 0.67969205 -1.02565107
C 5.15880537 0.77821406 1.11885008
H 4.35194331 1.50424411 1.24417209
H 5.14995637 0.10820801 1.98746314
H 6.11303346 1.31479509 1.12030708
C 6.09656144 -1.05737808 -0.36108603
H 5.99181541 -1.60557911 -1.30372309
H 7.07816651 -0.57388304 -0.36138103
H 6.08873343 -1.78827913 0.45649303

Figure S15. Aaa-Box-Aaa DMF Explicit

C 0.83434700 1.13374700 1.03658300
N 2.02623100 1.16621700 0.37632000
H 2.26477200 2.00874700 -0.14388100
C 2.97539400 0.13754800 0.24762500
C 4.31478700 0.47161000 -0.06601700
C 5.25848900 -0.54459800 -0.27344400
C 4.90482400 -1.89241500 -0.14638400
C 3.57778800 -2.20904000 0.18473800
C 2.62869100 -1.21603000 0.37648700
C 4.73369200 1.90801400 -0.19317400
O 3.95469200 2.80085100 -0.57310500
N 6.01763900 2.19483100 0.12665400
H 6.57953100 1.49371600 0.58715500
O 0.39096400 0.14347600 1.62658700
C 0.08122900 2.43246500 1.00579100
C -1.31791200 2.44242500 0.81217800
C -1.98526200 3.66807600 0.77512100
C -1.30118800 4.87328200 0.93895300
C 0.08276400 4.86180200 1.14910600
C 0.76177900 3.63763500 1.17925900
N -2.02027400 1.22122800 0.63386100
H -1.82397400 0.47136600 1.30299300
C -2.94407800 0.99971100 -0.33325400
O -3.35654900 1.88978300 -1.10223400
C -3.43995700 -0.41340000 -0.45048800
C -2.52887200 -1.46688500 -0.30618400
C -2.93954300 -2.79367900 -0.47115400
C -4.29101300 -3.04873800 -0.74846100
C -5.20203600 -2.01327200 -0.90451100
C -4.78931900 -0.67606000 -0.78983200
N -5.65687900 0.40718200 -1.01510700
H -5.16586700 1.28548600 -1.16709500
C -7.02627900 0.41551700 -1.06193200
O -7.73371000 -0.57734500 -0.89313200
H 2.42257930 0.86140902 0.33445712
C 0.61912707 -0.83139200 0.03118410
C-1.96262405 0.29159523 -0.17921592
H-0.93449686 2.19301531 -0.13737291
C 3.06250249 3.30483116 0.30483112
O 0.71533333 3.52025631 -0.15074691
C-0.53444306 -1.63725599 -0.00363990
C 1.97738514 -1.44444012 0.07324910
C-1.80997012 -1.11315188 -0.10694391
H 3.84919850 2.58181206 0.07323610
H 3.15524151 3.58947117 1.35854319
H 3.19694255 4.20425121 -0.29788792
H-0.46272812 -2.71643407 0.07777410
N 2.08186007 -2.74362822 -0.33397993
O 2.98997724 -0.83323914 0.45718713
C 3.37776312 -3.40055935 -0.38449793
H 1.32820599 -3.13866321 -0.87370297
H 3.22851505 -4.47997341 -0.45795094
H 3.98017918 -3.06115035 -1.23634599
H 3.93023217 -3.17226336 0.52805713
O-2.87969725 -1.97119988 -0.19130292
C-3.78565631 -1.93327982 0.91686016
H-4.27170429 -0.95707472 1.00486617
H-4.53835241 -2.69955984 0.72034815
H-3.26536728 -2.16857387 1.85489523
O-3.23479012 0.74812134 -0.31549593
C-3.44018405 2.15582645 -0.40668493
H-4.51551812 2.29008852 -0.52894694
H-3.10703999 2.67046747 0.50252513
H-2.91651898 2.58102545 -1.27077599

Figure S15. Aaa-Box-Aaa DMF Implicit

C -0.45722000 0.99912700 -0.33206600
N -1.72434300 1.04837000 0.16021100
H -2.07688300 1.92227100 0.54938400
C -2.72447800 0.06067400 0.05987700
C -4.08053700 0.46418100 0.04089700
C -5.09568500 -0.50191600 0.01307400
C -4.78717500 -1.86634000 -0.02885400
C -3.43659200 -2.25113400 -0.02411900
C -2.42016500 -1.30797100 0.02617300
C -4.44127900 1.92173100 0.07268200
O -3.71810500 2.77567700 0.62409500
N -5.59826900 2.27814500 -0.52579100
H -6.09058400 1.60288900 -1.09257400
O 0.01200900 -0.01190100 -0.88295100
C 0.35301000 2.25048000 -0.14444200
C 1.76916200 2.18277400 -0.02056300

O	0.85485600	5.96962800	1.33244500	C	2.48535700	3.36888300	0.17972900
H	6.27053100	-0.30110700	-0.56114700	C	1.83888000	4.60330700	0.23537400
H	3.26876600	-3.24825800	0.26965000	C	0.44988700	4.67697200	0.08230300
H	1.60913500	-1.49119800	0.59669600	C	-0.27753700	3.49757000	-0.10586300
H	-3.05458700	3.67859600	0.60726100	N	2.38736100	0.92107000	-0.11567100
H	-1.85760500	5.80232600	0.90977800	H	1.77127700	0.22205300	-0.53007000
H	1.83662900	3.64935300	1.32963300	C	3.62584000	0.53221500	0.29625300
H	-1.49460600	-1.25548000	-0.07951900	O	4.47456500	1.32484500	0.74146900
H	-4.63451100	-4.07373400	-0.86624800	C	3.90081100	-0.93904700	0.15166800
H	-6.23644200	-2.22811300	-1.12910900	C	2.83296600	-1.84876600	0.21400000
C	-7.63220100	1.77547600	-1.36759000	C	3.03898800	-3.22064900	0.05029200
H	-8.47369200	1.94274200	-0.69000500	C	4.34423500	-3.67810000	-0.18192200
H	-6.92405600	2.60357000	-1.27648400	C	5.41600000	-2.79809200	-0.22392100
H	-8.02719700	1.76355700	-2.38965700	C	5.22481100	-1.41743400	-0.04008600
C	0.21847300	7.24631700	1.30349700	N	6.29148500	-0.50615300	-0.03615700
H	-0.25931300	7.43288800	0.33366800	H	6.01797700	0.42396400	0.27994200
H	-0.52927000	7.33860000	2.10103400	C	7.60105100	-0.69835900	-0.40201900
H	1.01165500	7.97793100	1.46368400	O	8.06467200	-1.76500800	-0.80159100
C	-0.72174100	-3.92944400	-0.17429600	O	-0.28173000	5.82692100	0.09620400
O	-0.04943300	-2.91983300	0.03906600	H	-6.13429800	-0.20823500	0.04942100
C	-0.08094000	-5.30358000	-0.23617700	H	-3.17321800	-3.30616200	-0.03471300
H	-0.79660900	-6.12976800	-0.20901900	H	-1.39055600	-1.63506300	0.06373500
H	0.50199500	-5.37541800	-1.16100500	H	3.55884300	3.31945900	0.29019500
H	0.61372000	-5.40003200	0.60219300	H	2.43438000	5.49608800	0.38417300
C	7.14925400	-2.91370400	-0.62999300	H	-1.35171300	3.58748600	-0.23462700
O	7.78964200	-1.86996300	-0.74305200	H	1.82857600	-1.50294900	0.40990200
C	7.78822100	-4.27767600	-0.83020200	H	4.52980200	-4.74078400	-0.31692500
H	8.85907400	-4.19250900	-0.63771900	H	6.41575200	-3.16816600	-0.39600700
H	7.36022100	-5.04624500	-0.17912100	C	8.44688400	0.55955200	-0.27828200
H	7.64833300	-4.59614300	-1.87009800	H	8.10462300	1.32197100	-0.98759200
C	6.52585100	3.55694900	0.05918700	H	8.37728400	0.98648600	0.72831300
H	6.06269700	4.19499600	0.82130800	H	9.48614400	0.30958700	-0.49528100
H	7.60553200	3.53547100	0.21542700	C	0.40997900	7.06182900	0.27678400
H	6.31050800	3.98606300	-0.92277000	H	0.93034800	7.09131700	1.24219100
N	5.80530700	-2.95757000	-0.34875400	H	1.13123100	7.23785500	-0.53104900
H	5.39635400	-3.88048200	-0.27527000	H	-0.35628600	7.83789500	0.25437100
N	-2.07331700	-3.90574900	-0.38504900	C	0.70822500	-4.01712600	0.52189800
H	-2.51364200	-4.80014700	-0.55746400	O	0.27602800	-2.96859000	1.00120200
N	-2.18341600	-3.02991900	2.95603400	C	-0.18384000	-5.23281300	0.34800500
C	-1.29528200	-4.17260000	3.09160600	H	0.36211700	-6.16648300	0.18705500
C	-1.70624700	-1.77906600	2.78487000	H	-0.81542200	-5.32948900	1.23444200
H	-1.37884600	-4.61458400	4.09176300	H	-0.84025200	-5.06189400	-0.51312300
H	-0.26229500	-3.85982500	2.92352200	C	-7.12899300	-2.77070500	-0.11685800
H	-1.55529200	-4.93819600	2.35214100	O	-7.72265000	-1.69520400	-0.16489700
H	-0.61223800	-1.73257200	2.70427800	C	-7.86663300	-4.09817400	-0.08062300
N	-0.43642500	0.46099000	-2.73666700	H	-8.84520200	-3.96564900	-0.54530800
C	-0.28855400	1.78443500	-2.48244100	H	-7.32539000	-4.90035800	-0.59155900
C	-1.64484000	-0.05456700	-3.36665000	H	-8.01822900	-4.40047500	0.96243700

C	0.68446400	-0.45933800	-2.59686400
O	0.74857600	2.33654000	-2.11365700
H	-1.23019600	2.34212800	-2.61950800
H	-2.46383600	0.65512800	-3.22992400
H	-1.93131900	-0.99941900	-2.89733600
H	-1.48336300	-0.22917400	-4.43866700
H	1.53449900	0.08531000	-2.18835000
H	0.95676300	-0.88236500	-3.57233800
H	0.41819300	-1.27654400	-1.91695400
O	-2.40460700	-0.76198900	2.70078600
C	-3.61445000	-3.28665600	3.02131400
H	-3.88039700	-3.73080600	3.98770300
H	-3.90562300	-3.97807000	2.22238400
H	-4.14365100	-2.34299400	2.89213700

Figure S15. Aaa-Box-Aaa MeOH Explicit

C	-0.43800600	1.15851700	-0.32486000
N	-1.63850600	1.25771200	0.29420400
H	-1.96308100	2.17043200	0.60852700
C	-2.65722100	0.27639100	0.31029900
C	-4.00015500	0.67364900	0.13521900
C	-5.02033500	-0.28435800	0.19055500
C	-4.72001700	-1.63842600	0.39240600
C	-3.37883700	-2.01428300	0.56947500
C	-2.36219500	-1.07035400	0.54088200
C	-4.33639400	2.12337600	-0.07012100
O	-3.64834600	3.03694400	0.42549100
N	-5.42882200	2.39714200	-0.81388400
H	-5.88573700	1.64907600	-1.31544200
O	-0.04646800	0.10821400	-0.87482500
C	0.42158100	2.38621900	-0.27761700
C	1.83982600	2.27901800	-0.27578600
C	2.60468900	3.44538900	-0.16885000
C	2.00157100	4.70006700	-0.09423100
C	0.60667900	4.81298800	-0.13865300
C	-0.16880300	3.65294300	-0.22881000
N	2.41875500	1.00129500	-0.40038900
H	1.77188800	0.32176800	-0.79295000
C	3.62733400	0.55948600	0.05067500
O	4.48790400	1.31787400	0.52870200
C	3.84006900	-0.92192000	-0.08198800
C	2.72850400	-1.77578500	-0.08244000
C	2.86770900	-3.15609900	-0.22343600
C	4.15336700	-3.68757100	-0.38256900
C	5.27191500	-2.86317400	-0.36978500
C	5.14500800	-1.47377900	-0.19535400
N	6.25398400	-0.61714800	-0.12663500
H	6.00905400	0.32413300	0.17781300
C	-6.03373700	3.66712100	-0.56121800
H	-5.36658100	4.27648100	-1.18187500
H	-7.04390100	3.70580200	-0.97118900
H	-6.03637800	4.08291500	0.44968800
N	-5.76037800	-2.88397400	-0.05718800
H	-5.39240800	-3.82649500	-0.03055600
N	1.99623400	-4.17276300	0.07897200
H	2.25272300	-5.09785300	-0.24071000

Figure S15. Aaa-Box-Aaa MeOH Implicit

C	-0.45741700	0.99910700	-0.33156200
N	-1.72466800	1.04857200	0.16043400
H	-2.07725800	1.92257200	0.54933800
C	-2.72471000	0.06081300	0.06024800
C	-4.08079700	0.46421400	0.04095100
C	-5.09586200	-0.50197400	0.01326900
C	-4.78721300	-1.86634900	-0.02824300
C	-3.43661500	-2.25104000	-0.02319500
C	-2.42025200	-1.30781600	0.02697800
C	-4.44166300	1.92174400	0.07220600
O	-3.71849300	2.77604300	0.62296400
N	-5.59890200	2.27778400	-0.52613700
H	-6.09137000	1.60219900	-1.09237600
O	0.01180100	-0.01205000	-0.88215800
C	0.35284100	2.25045800	-0.14405500
C	1.76900200	2.18270900	-0.02027400
C	2.48522900	3.36881000	0.17994000
C	1.83877000	4.60323800	0.23556800
C	0.44978500	4.67694300	0.08255600
C	-0.27768000	3.49755900	-0.10549900
N	2.38718400	0.92100300	-0.11542000
H	1.77094900	0.22191400	-0.52949000
C	3.62585900	0.53231000	0.29609100
O	4.47468500	1.32511400	0.74079900
C	3.90096200	-0.93895100	0.15164700
C	2.83324100	-1.84880400	0.21406300
C	3.03950600	-3.22064000	0.05035200
C	4.34479800	-3.67792100	-0.18185700
C	5.41645000	-2.79780000	-0.22395400
C	5.22503200	-1.41717700	-0.04017100
N	6.29158600	-0.50578700	-0.03636600
H	6.01792400	0.42429200	0.27974300
C	7.60117900	-0.69791400	-0.40224900
O	8.06495200	-1.76448900	-0.80172800
O	-0.28179800	5.82693200	0.09643200
H	-6.13451600	-0.20843300	0.04952400
H	-3.17316600	-3.30606900	-0.03342900
H	-1.39061400	-1.63479300	0.06473300

C	7.57352100	-0.88383300	-0.39304600
O	8.00281200	-1.97313900	-0.77025300
O	-0.08638200	5.98475200	-0.10377100
H	-6.05572600	0.00949400	0.10195200
H	-3.12227800	-3.05580500	0.74490700
H	-1.34193300	-1.38996100	0.70680900
H	3.68221800	3.36474300	-0.14758700
H	2.63223100	5.57782600	-0.02047900
H	-1.24688800	3.77385200	-0.27683200
H	1.73271600	-1.37959800	0.04492400
H	4.28528400	-4.75977100	-0.50258400
H	6.25906800	-3.28528700	-0.48577000
C	8.50232000	0.29438500	-0.14925400
H	7.99389100	1.26224000	-0.17975000
H	8.96916100	0.18047900	0.83616300
H	9.29582100	0.27530900	-0.89984600
C	0.65263000	7.20278700	-0.01636100
H	1.24736700	7.24387700	0.90451800
H	1.31251700	7.33265100	-0.88307600
H	-0.09095100	8.00070800	-0.00355600
C	0.53078100	-3.81559700	0.33953600
O	0.28678200	-2.84288800	1.05594500
C	-0.52947500	-4.84367500	0.01071800
H	-0.12733300	-5.79610600	-0.34650800
H	-1.14443300	-5.01435700	0.89757700
H	-1.15610700	-4.40203600	-0.77214800
C	-7.05455300	-2.55549500	0.24081100
O	-7.63163700	-1.50470400	-0.02716800
C	-7.78398400	-3.88220600	0.36681000
H	-7.42841800	-4.59361700	-0.38730200
H	-7.61838000	-4.32950900	1.35329800
H	-8.85115500	-3.71015700	0.22302000
C	-5.84060700	3.76881300	-1.07769200
H	-5.10901900	4.28742600	-1.70790700
H	-6.80633000	3.75186300	-1.58452600
H	-5.93398000	4.31816400	-0.13722500
N	-5.69744300	-2.64820500	0.45132100
H	-5.34311900	-3.57489700	0.65408300
N	1.74923600	-4.01945800	-0.25517500
H	1.87992900	-4.89599200	-0.74290900
C	-2.02126600	-1.81122900	-2.86488700
H	-1.73310200	-0.93744400	-3.46943700
H	-2.31701600	-2.61528900	-3.54644100
H	-2.89565200	-1.53508300	-2.25699200
C	1.23615000	0.23432900	2.73323300
H	0.54570400	0.97709500	2.32369300
H	2.26252000	0.58013000	2.53763400
H	1.07643400	-0.71742000	2.20921800
H	3.55870400	3.31932100	0.29043400
H	2.43429200	5.49601200	0.38433800
H	-1.35186900	3.58752800	-0.23412500
H	1.82879900	-1.50317200	0.41006800
H	4.53052200	-4.74059800	-0.31679400
H	6.41626300	-3.16768800	-0.39609700
C	8.44683500	0.56015100	-0.27862300
H	8.10442200	1.32249400	-0.98794900
H	8.37726000	0.98712500	0.72796300
H	9.48610100	0.31028600	-0.49569500
C	0.40991400	7.06174100	0.27718500
H	0.93014900	7.09117300	1.24267900
H	1.13131100	7.23782400	-0.53052600
H	-0.35631200	7.83785000	0.25471300
C	0.70869800	-4.01749900	0.52144500
O	0.27596900	-2.96899100	1.00017000
C	-0.18293000	-5.23358200	0.34766000
H	0.36332100	-6.16719700	0.18725800
H	-0.81476500	-5.33010500	1.23393100
H	-0.83909800	-5.06317100	-0.51375300
C	-7.12898800	-2.77083700	-0.11712800
O	-7.72261900	-1.69544200	-0.16670200
C	-7.86661500	-4.09831500	-0.07996200
H	-8.84480900	-3.96635000	-0.54558500
H	-7.32493700	-4.90106900	-0.58956800
H	-8.01907300	-4.39944200	0.96331500
C	-6.03445800	3.66670900	-0.56200300
H	-5.36761800	4.27584900	-1.18321700
H	-7.04483800	3.70516000	-0.97149300
H	-6.03658700	4.08294000	0.44871800
N	-5.76039100	-2.88404900	-0.05641100
H	-5.39240500	-3.82652100	-0.02879800
N	1.99692100	-4.17297100	0.07904000
H	2.25371900	-5.09805700	-0.24035700

O -0.94534100 -2.29484700 -2.07332200<br>H -0.66274300 -1.55182700 -1.50671300<br>O 0.95487900 0.13695500 4.12886100<br>H 1.53266600 -0.55320700 4.48918400	

SI References

(1) Naserifar, S.; Brooks, D. J.; Goddard, W. A.; Cvicek, V. Polarizable Charge Equilibration Model for Predicting Accurate Electrostatic Interactions in Molecules and Solids. *J. Chem. Phys.* **2017**, *146* (12), 124117.

(2) Naserifar, S.; Goddard, W. A. The Quantum Mechanics-Based Polarizable Force Field for Water Simulations. *J. Chem. Phys.* **2018**, *149* (17), 174502.

(3) Naserifar, S.; Oppenheim, J. J.; Yang, H.; Zhou, T.; Zybin, S.; Rizk, M.; Goddard, W. A. Accurate Non-Bonded Potentials Based on Periodic Quantum Mechanics Calculations for Use in Molecular Simulations of Materials and Systems. *J. Chem. Phys.* **2019**, *151* (15), 154111.

(4) Mayo, S. L.; Olafson, B. D.; Goddard, W. A. DREIDING: A Generic Force Field for Molecular Simulations. *J. Phys. Chem.* **1990**, *94* (26), 8897–8909.

(5) Upadhyayula, S.; Bao, D.; Millare, B.; Sylvia, S. S.; Habib, K. M. M.; Ashraf, K.; Ferreira, A.; Bishop, S.; Bonderer, R.; Baqai, S.; Jing, X.; Penchev, M.; Ozkan, M.; Ozkan, C. S.; Lake, R. K.; Vullev, V. I. Permanent Electric Dipole Moments of Carboxyamides in Condensed Media: What Are the Limitations of Theory and Experiment? *J. Phys. Chem. B* **2011**, *115* (30), 9473–9490.

(6) Onsager, L. Electric Moments of Molecules in Liquids. *J. Am. Chem. Soc.* **1936**, *58*, 8.

(7) Daniels, I. N.; Wang, Z.; Laird, B. B. Dielectric Properties of Organic Solvents in an Electric Field. *J. Phys. Chem. C* **2017**, *121* (2), 1025–1031.

(8) Krzeszewski, M.; Espinoza, E. M.; Červinka, C.; Derr, J. B.; Clark, J. A.; Borchardt, D.; Beran, G. J. O.; Gryko, D. T.; Vullev, V. I. Dipole Effects on Electron Transfer Are Enormous. *Angew. Chem. Int. Ed.* **2018**, *57* (38), 12365–12369.

(9) Emamian S.; Lu, T.; Kruse, H.; Emamian, H. Exploring Nature and Predicting Strength of Hydrogen Bonds: A Correlation Analysis Between Atoms-in-Molecules Descriptors, Binding Energies, and Energy Components of Symmetry-Adapted Perturbation Theory. *J. Comput. Chem.* **2013**, *40* (32), 2868–2881.

(10) Frisch, M. J. et al, in Gaussian 09, Revision D.01, Vol. Gaussian 09, Revision D.01, Gaussian 09, Revision D.01 ed., Gaussian, Inc., Wallingford, **2013**.

(11) Becke, A. D. Density-Functional Thermochemistry. III. The Role of Exact Exchange. *J. Chem. Phys.* **1993**, *98*, 5648–5652.

(12) Lee, C.; Yang, W.; Parr, R. G. Development of the Colle-Salvetti correlation-energy formula into a functional of the electron density. *Phys. Rev. B* **1988**, *37*, 785.

(13) Cossi, M.; Barone, V.; Cammi, R.; Tomasi, J. Ab Initio Study of Solvated Molecules: A New Implementation of the Polarizable Continuum Model, *Chem. Phys. Lett.* **1996**, *255*, 327-335.

(14) NBO Version 3.1, E. D. Glendening, A. E. Reed, J. E. Carpenter, and F. Weinhold.

(15) Lu, T.; Chen, Q. A Simple Method of Identifying π Orbitals for Non-Planar Systems and a Protocol of Studying π Electronic Structure. *Theor. Chem. Acc.* **2020**, *139*, 25.

(16) Lopes, T. O.; Machado, D. F. S.; Risko, C.; Brédas, J.-L.; de Oliveira, H. C. B. Bond Ellipticity Alternation: An Accurate Descriptor of the Nonlinear Optical Properties of π-Conjugated Chromophores. *J. Phys. Chem. Lett.* **2018**, *9*, 1377–1383.
